# Supplementary material for: Biocompatible sulfonium-based covalent probes for endogenous tubulin fluorescence nanoscopy in live and fixed cells
Source: Nat Commun. 2025 Dec 11;16:11107. doi: 10.1038/s41467-025-67319-x (PMC12701065; doi:10.1038/s41467-025-67319-x)
Supplement: Supplementary file 1 — Supplementary information [file 41467_2025_67319_MOESM1_ESM.pdf]

Supplementary information contents

Supplementary Figures **3**

Supplementary Figure 1. Detailed structures of the covalent probes and non-covalent tubulin probe SiR-CTX[1] 3

Supplementary Figure 2. Absorption and emission spectra of covalent tubulin probes. 4

Supplementary Figure 3. Absorbance and fluorescence emission of covalent tubulin probes in different conditions. 5

Supplementary Figure 4. Behavior of Tubulin Probes in 1,4-Dioxane–Water Mixtures. 6

Supplementary Figure 5. Stability of covalent tubulin probes in PBS pH=7.4 at 37°C. 7

Supplementary Figure 6. Full representative *in-gel* fluorescence and Coomassie staining for kinetic (Figure 2b). 8

Supplementary Figure 7. Stability of the covalent bond between the dye and tubulin in PBS pH=7.4 at 37°C. .8

Supplementary Figure 8. Identified labeled peptides after *in-gel* analysis of SiR-labeled tubulin. 9

Supplementary Figure 9. MS spectra of identified labeled peptides. 10

Supplementary Figure 10. Structural models of tubulin taxane binding sites and labeled Cys residues. 11

Supplementary Figure 11. Sequence alignment of β-tubulin isotypes from human (Homo sapiens) and pig (Sus scrofa). 13

Supplementary Figure 12. Sequence alignment of α-tubulin isotypes from human (Homo sapiens) and pig (Sus scrofa). 14

Supplementary Figure 13. Cell cycle perturbation induced by covalent tubulin probes. 15

Supplementary Figure 14. Live U-2 OS cells stained with probes (1 µM in OptiMEM), verapamil (10 µM) and Hoechst 33342 (1 µg/mL) for 4h. 16

Supplementary Figure 15. Live HeLa CCL cells stained with probes (1 µM in OptiMEM) and Hoechst 33342 (1 µg/mL) for 4h. 17

Supplementary Figure 16. Live U-2 OS, HeLa CCL cells and human fibroblasts stained with 1µM 6-SiR-o-C_9-CTX in OptiMEM for 4h after extensive washing procedure. 18

Supplementary Figure 17. Statistical analysis associated with the cell cycle analysis of washing experiment. 19

Supplementary Figure 18. Representative images used to measure apparent microtubule FWHM. 20

Supplementary Tables **21**

Supplementary Table 1. Photophysical properties of the probes (1-6). 21

Supplementary Table 2. Identified labeled peptides in the sample. 21

Supplementary Table 3. Cytotoxicity threshold of the probes (1-6). The indicated probe concentration represents the lowest concentration tested, at which a cytotoxicity effect was observed. 22

Supplementary Table 4. Acquisition parameters for microscopy images 23

Supplementary Methods **24**

Determination of absolute quantum yields 24
Determination of fluorescence lifetimes... 24
Measurements of absorbance spectra in 1,4-dioxane–water mixtures 24
Maintenance and preparation of the cells 25
Chemical Synthesis of the building blocks... 25
Characterization of the final probes.. 34
Supplementary NMR spectra..**46**
Supplementary references **90**

Supplementary Figures

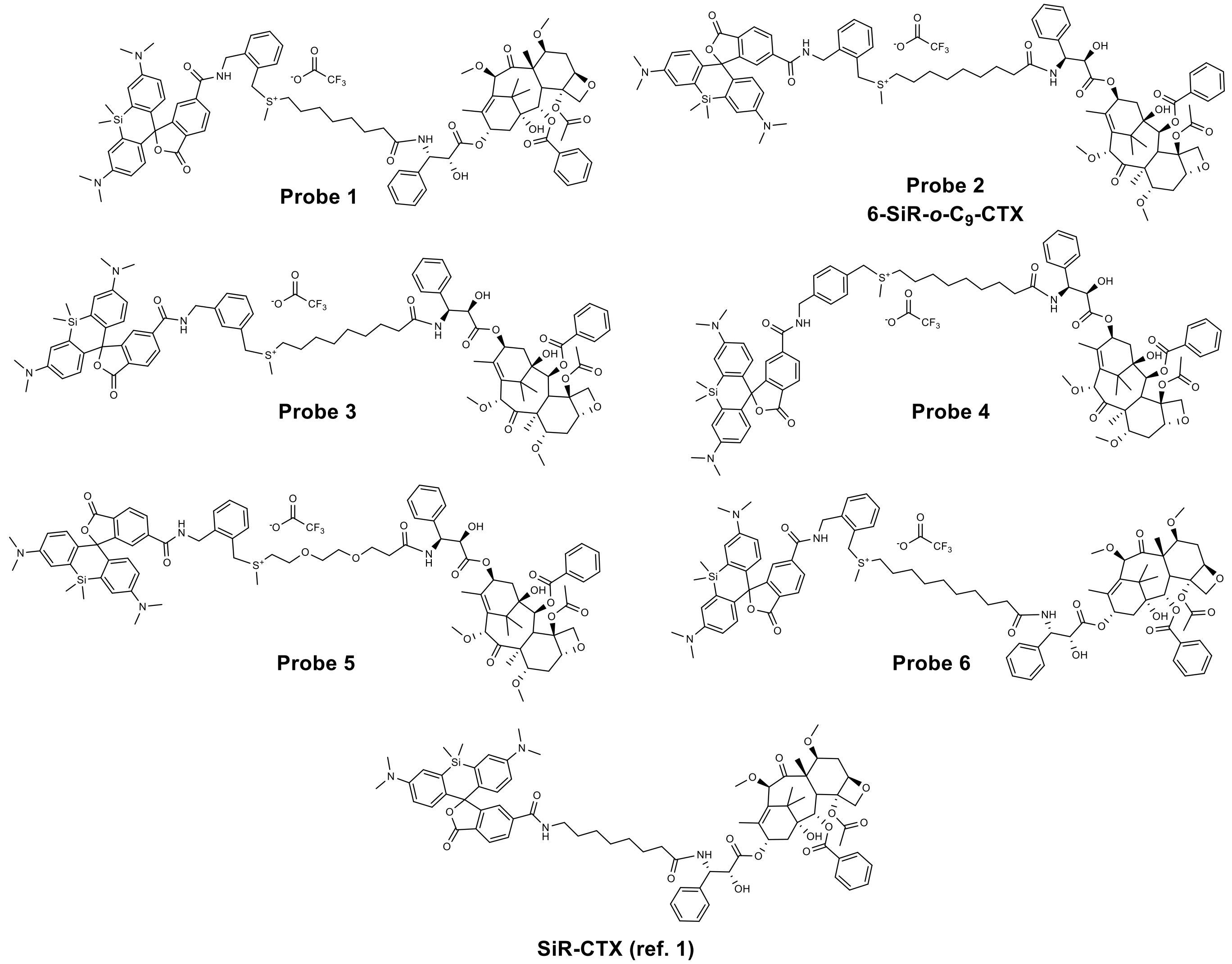

Supplementary Figure 1. Detailed structures of the covalent probes and non-covalent tubulin probe SiR-CTX[1]

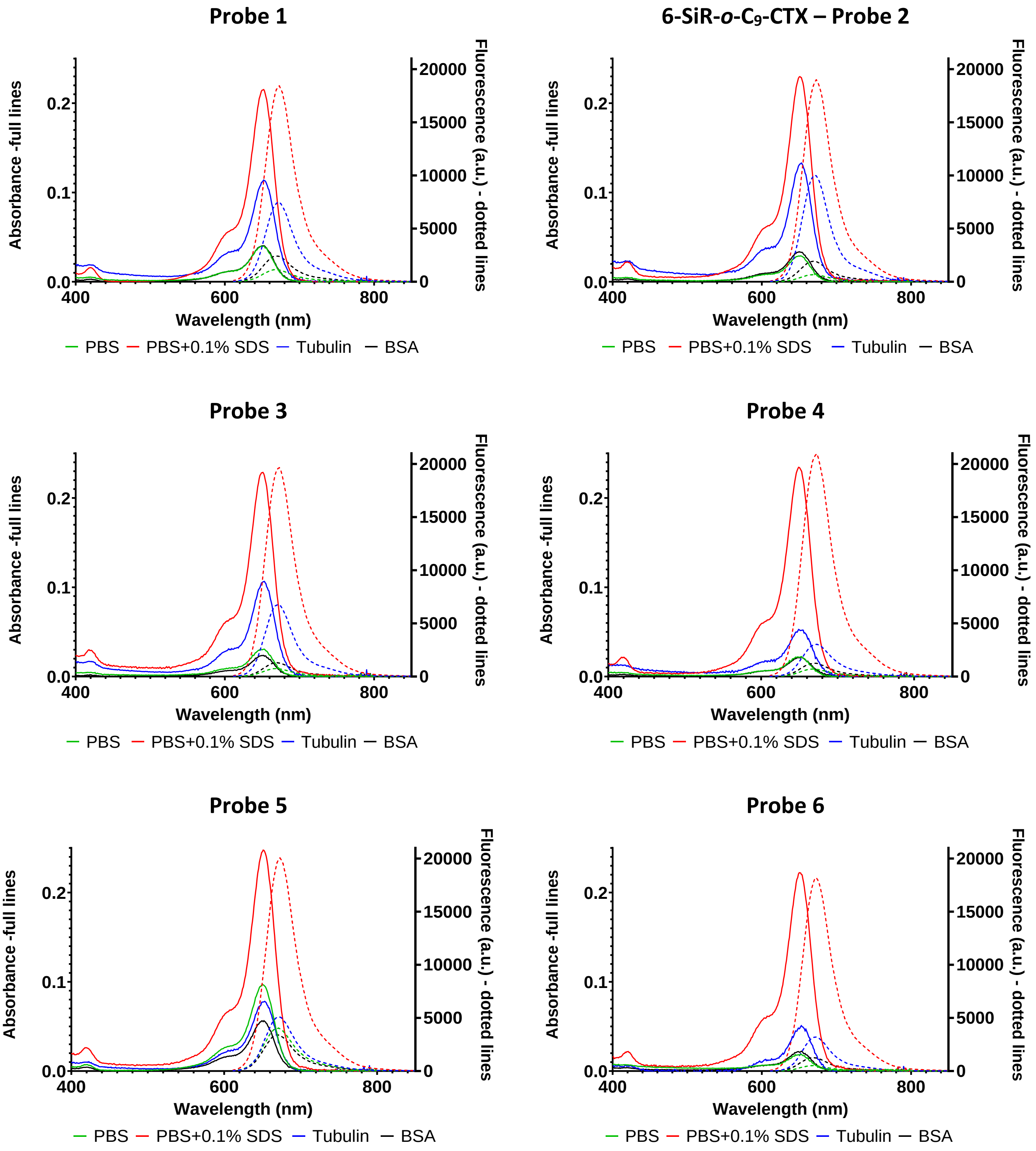

Supplementary Figure 2. Absorption and emission spectra of covalent tubulin probes. Spectra were recorded after incubating 5 µM probes with 0.5 mg/mL tubulin in General Tubulin Buffer (Blue), 0.5 mg/mL BSA in PBS (Black), in PBS (Green), or in PBS+0.1% SDS (Red) at 37°C for 4h. Spectra are represented as averages of three independently repeated experiments (N=3).

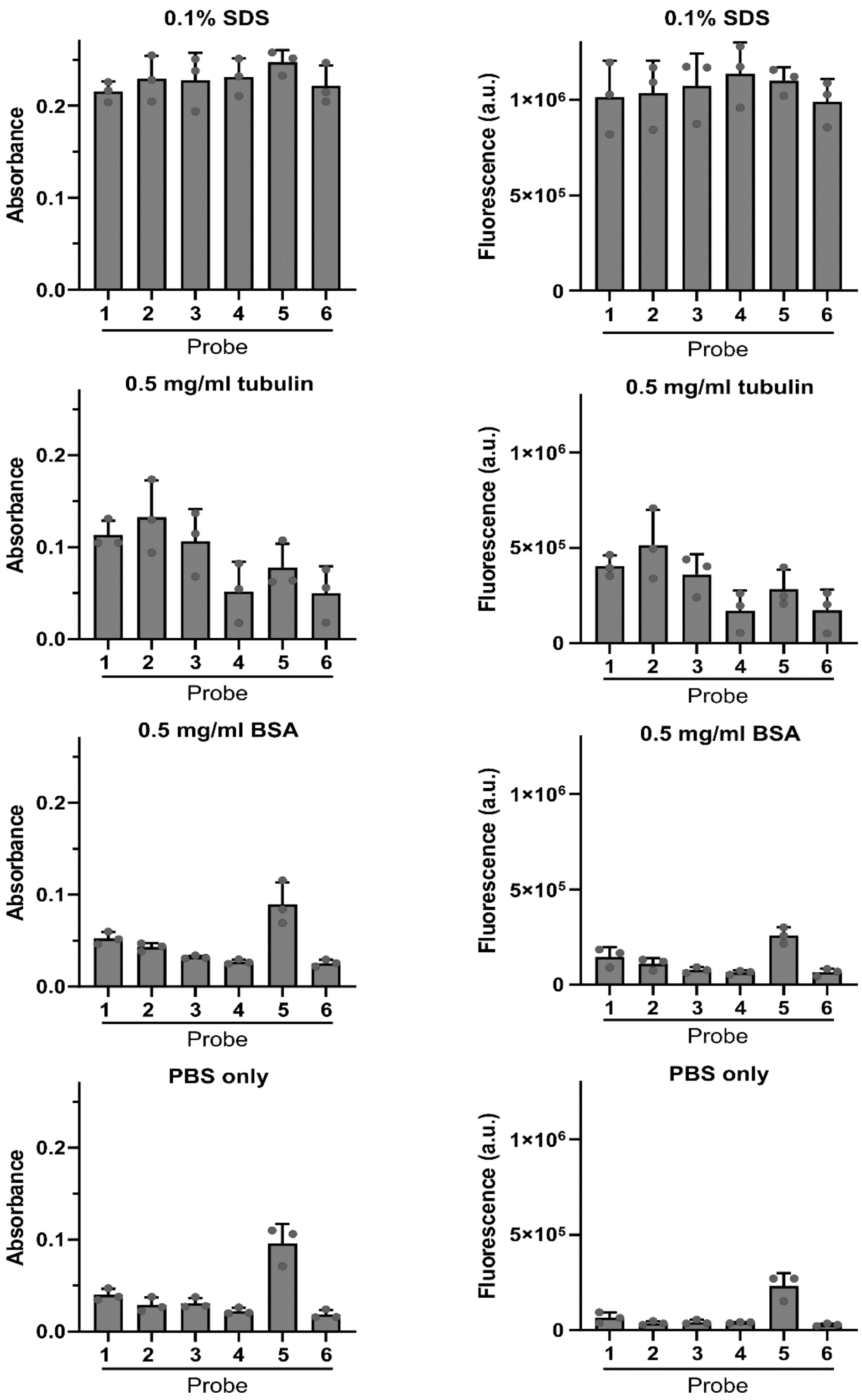

Supplementary Figure 3. Absorbance and fluorescence emission of covalent tubulin probes in different conditions. Spectra were recorded after incubating 5 µM probes with 0.5 mg/mL tubulin in General Tubulin Buffer, 0.5 mg/mL BSA in PBS and in 0.1% SDS in PBS, only in PBS at 37°C for 4h. Absorbance at the maximum (652 nm) and integration of fluorescence spectra (between 610 and 850 nm) are represented as averages of three independently repeated experiments (N=3) with standard deviations.

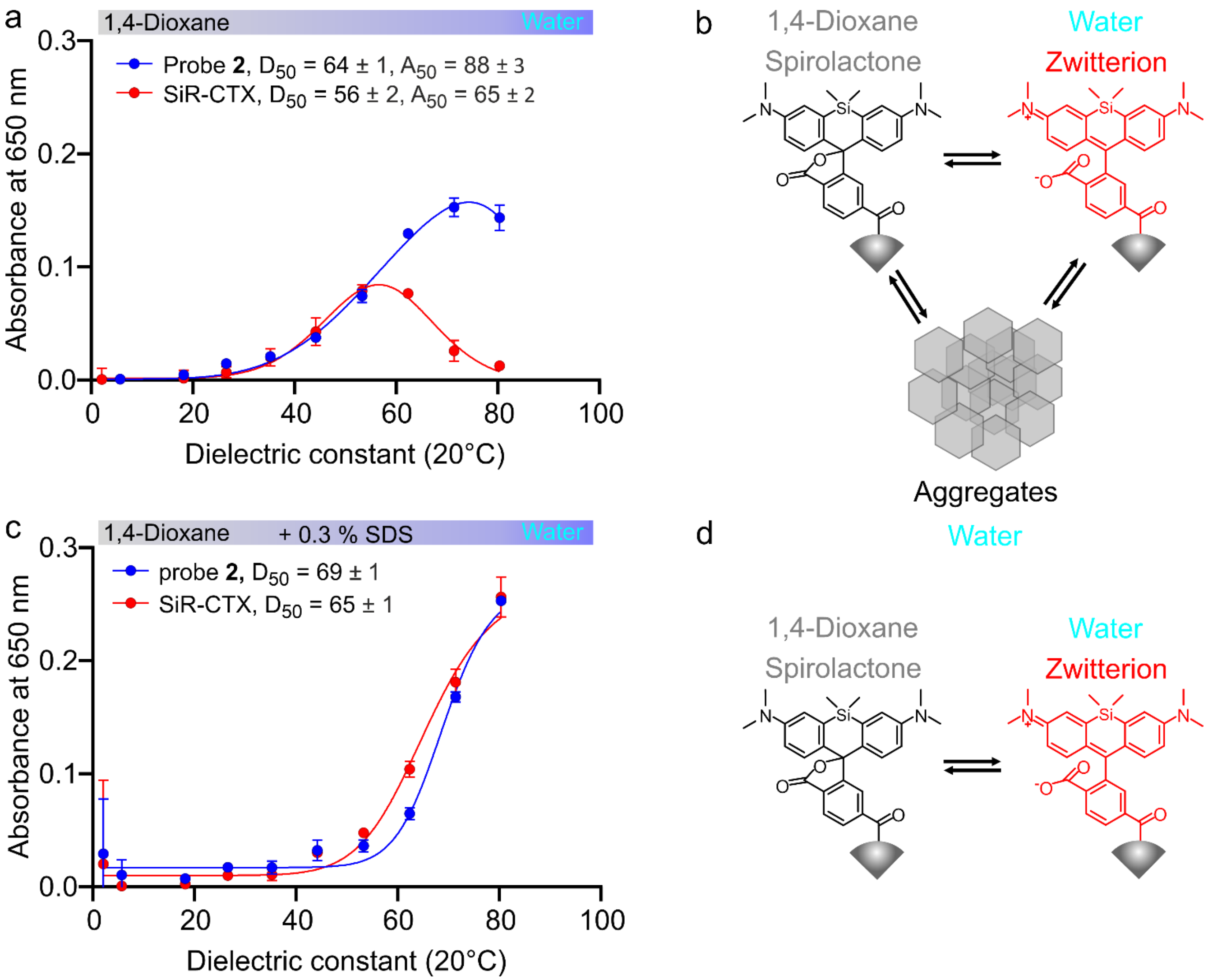

Supplementary Figure 4. Behavior of Tubulin Probes in 1,4-Dioxane–Water Mixtures. (**a**) Absorbance of probe 2 and SiR-CTX at 650 nm plotted against the dielectric constant of 1,4-dioxane–water mixtures. The data were fitted to a bell-shaped dose–response curve, representing two processes: spirolactonization and aggregation, as illustrated in panel (**b**). (**c**) Absorbance of probe 2 and SiR-CTX at 650 nm versus dielectric constant in the presence of 0.3% sodium dodecyl sulfate (SDS). The detergent prevents aggregation, allowing observation of the equilibrium between the spirolactone and zwitterionic forms, as shown in panel (**d**). D_{50} - corresponds to dielectric constant value that provokes half of the absorbance amplitude in the ascending dose-response curve part, A_{50} - corresponds to dielectric constant value that provokes half of the absorbance amplitude in the declining dose-response curve part All data points are presented as mean ± s.d. from three technical replicates (N = 3). Fitted values are represented as mean ± s.e.m.

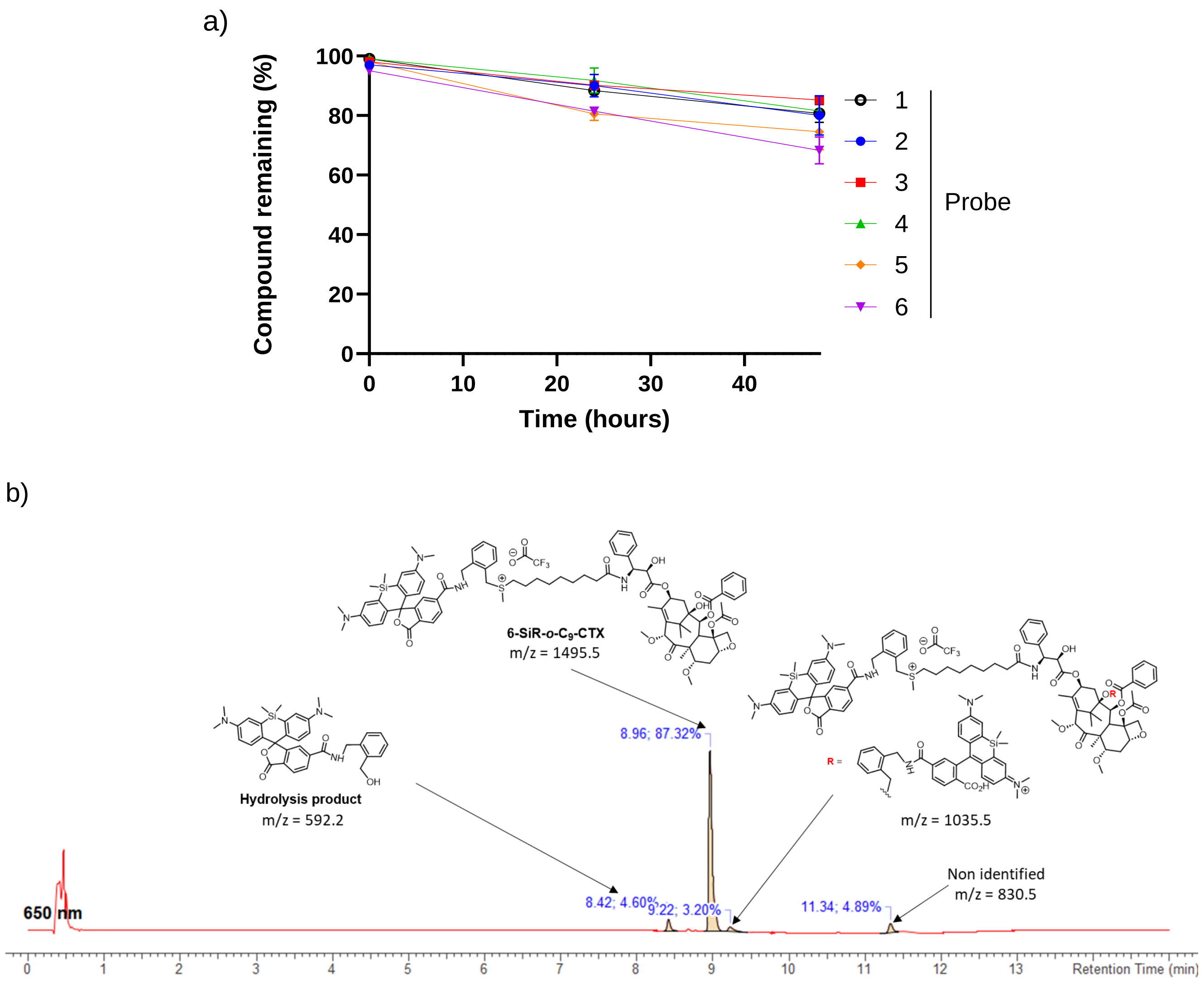

Supplementary Figure 5. Stability of covalent tubulin probes in PBS pH=7.4 at 37°C. Solution of covalent probes (20 µM in PBS) were heated at 37°C in the dark. Solutions were analyzed after 0, 24h and 48h by LC/MS. (**a**) The percentage of compound remaining over time according is plotted. (**b**) Examples of degradation products observed after 48h.

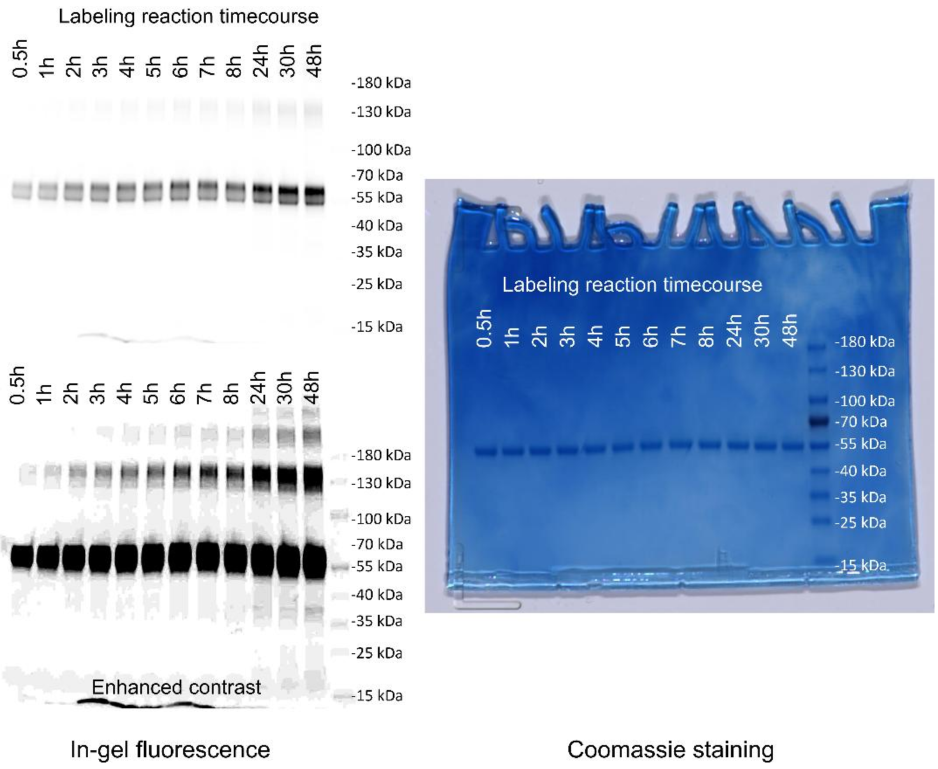

Supplementary Figure 6. Full representative *in-gel* fluorescence and Coomassie staining for kinetic (Figure 2b).

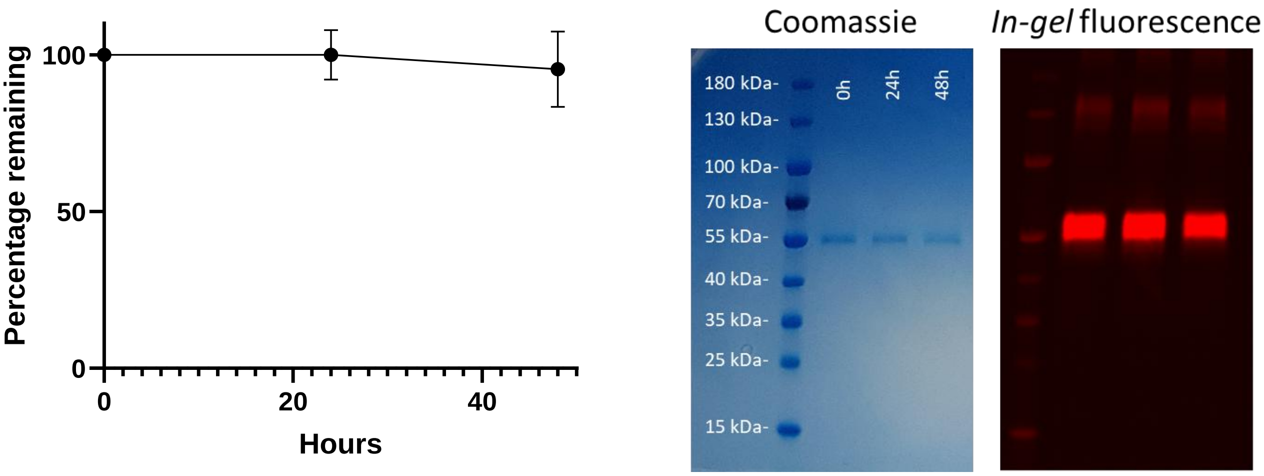

Supplementary Figure 7. Stability of the covalent bond between the dye and tubulin in PBS pH=7.4 at 37°C. The experiment was performed in triplicate (N=3) to obtain mean and standard deviation values (shown as error bars).

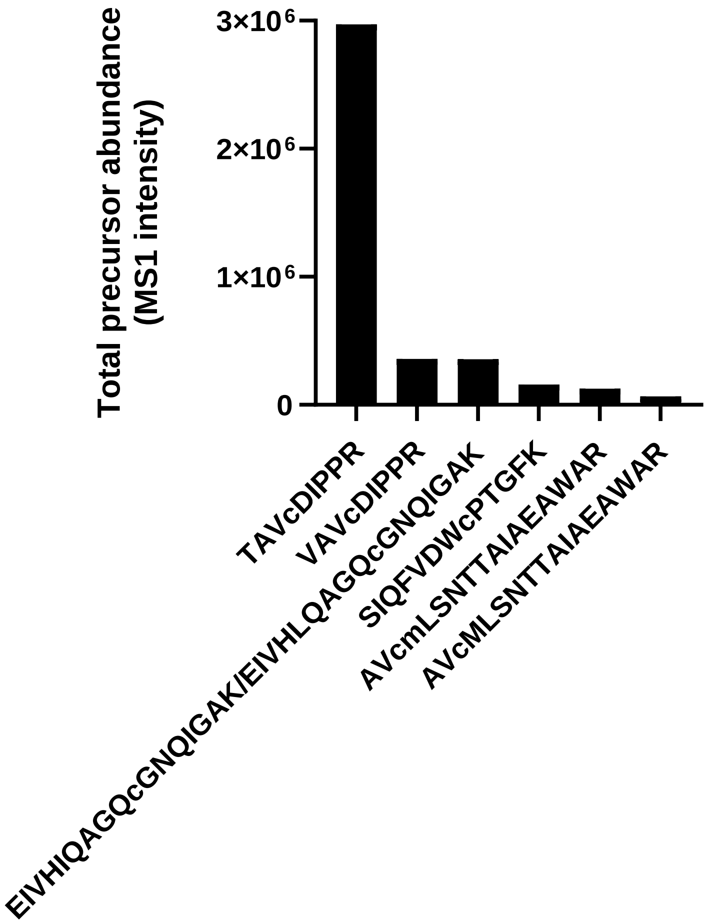

Supplementary Figure 8. Identified labeled peptides after *in-gel* analysis of SiR-labeled tubulin. c is the modified cysteine. m is an oxidized methionine.

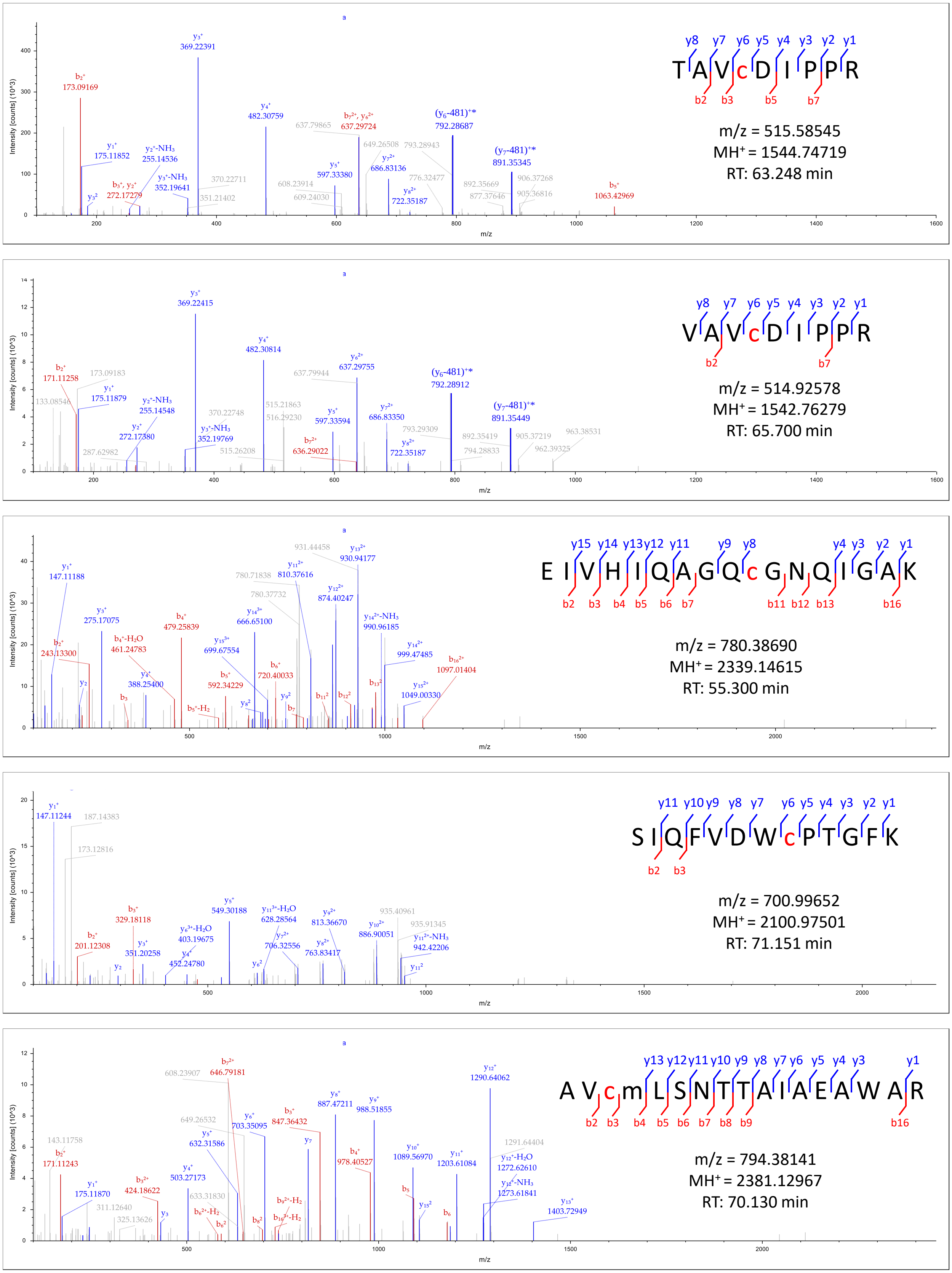

Supplementary Figure 9. MS spectra of identified labeled peptides. c is the modified cysteine. m is an oxidized methionine. Ions marked with a star (*) correspond to fragment ions with a mass loss of 481 Da, which may be caused by fragmentation of the label.

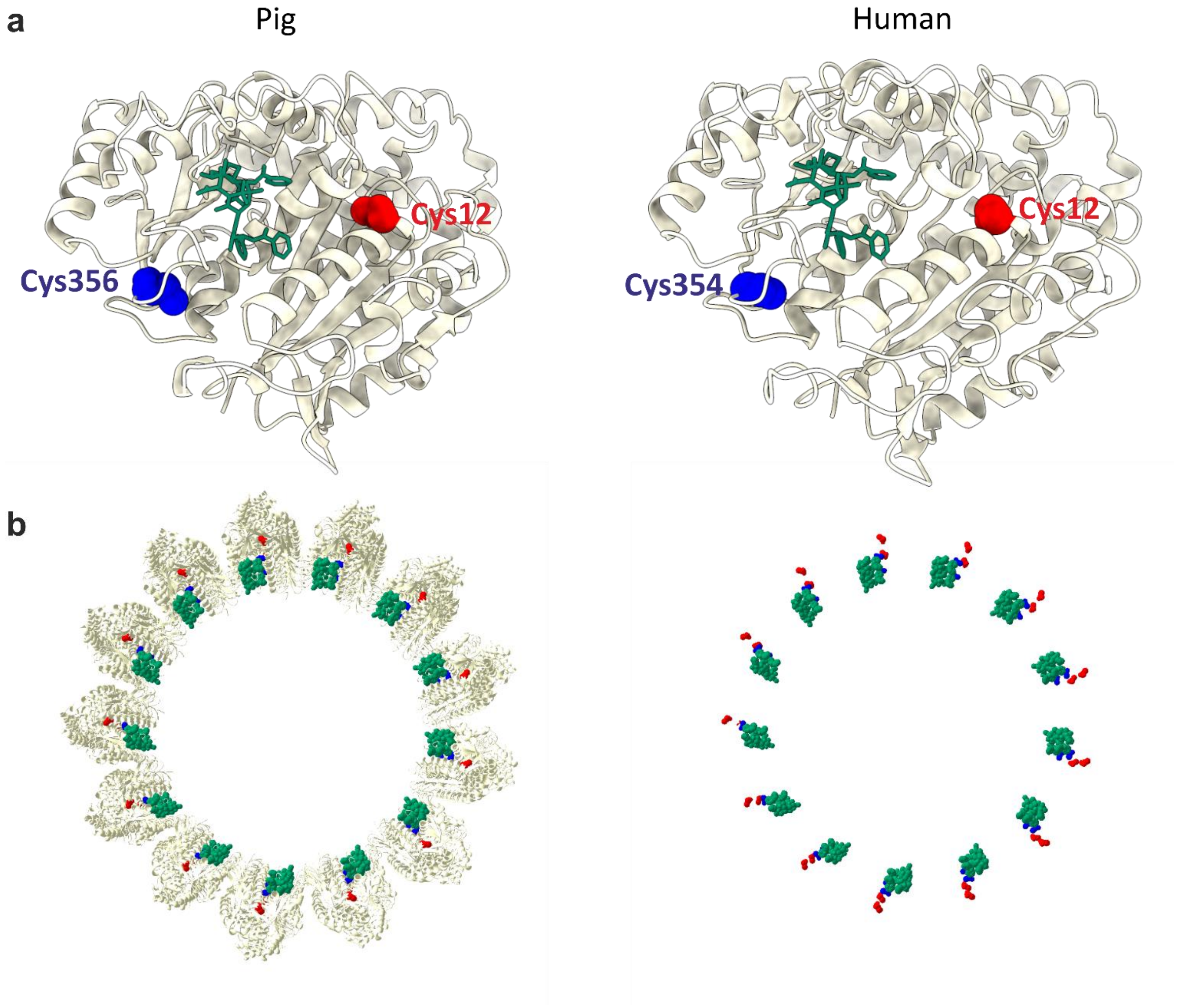

Supplementary Figure 10. Structural models of tubulin taxane binding sites and labeled Cys residues. (**a**) Comparison of cryo-EM β-tubulin complex with taxol from pig (PDB: 5SYF) and model of human β-tubulin complex with taxol (model is based on cryo-EM structure PDB: 6E7C). (**b**) Microtubule-taxol complex model (PDB: 5SYF). Left panel shows top view of microtubule composed of tubulin dimers, taxol and the main labeling site (Cys356). Right panel shows same view as on left, but protein molecules are omitted. Protein molecule is shown in white, Taxol is green, the main labeling site (Cys356 or Cys354) is highlighted in blue, and the minor labeling site (Cys12) in red. GTP, GDP and Mg^{2+} molecules are omitted for clarity.

```
                                        10        20        30        40        50        60        70        80        90
                                ....|....|....|....|....|....|....|....|....|....|....|....|....|....|....|....|....|....|
  TUBB2A NP_001060        1     ...................................................MREIVHIQAGQCGNQIGAK....................
H TUBB4A NP_001276052     1     MRRGAADRETERLPGAQGPCSAVSAASSTLAAAAAPRARATAAASTLSATAMREIVHLQAGQCGNQIGAK....................
U TUBB   NP_001280141     1     ......................................MGDVGPRWANWDPSRSGVRDHPGQQSETLPHATFL.................
M TUBB3  NP_006077        1     ...................................................MREIVHIQAGQCGNQIGAK....................
A TUBB1  NP_110400        1     ...................................................MREIVHIQIGQCGNQIGAK....................
N TUBB6  NP_115914        1     ...................................................MREIVHIQAGQCGNQIGTK....................
  TUBB8  NP_817124        1     ...................................................MREIVLTQIGQCGNQIGAK....................
  --------------------------------------------------------------------------------------------------------------------------
  TUBB   Q767L7           1     ...................................................MREIVHIQAGQCG....NQIG..................
P TUBB2A A0A287AHL4       1     ...................................................MREIVHIQAGQCGNQIGAK....................
I TUBB2B A0A287AEI4       1     ...................................................MREIVHIQAGQCGNQIGAK....................
G TUBB3  A0A5G2R693       1     ...................................................MREIVHIQAGQCGNQIGAK....................
  TUBB4A A0A480UV25       1     ...................................................MREIVHLQAGQCGNQIGAK....................
  TUBB4B A0A287A217       1     ................................YRPSAAAAVASAPAVAAAAMREIVHLQAGQCGNQIGAKVSWGIGSGADPPGGAGGRQE
                                                                                 Detected labeled peptide (minor)

                                       100       110       120       130       140       150       160       170       180
                                ....|....|....|....|....|....|....|....|....|....|....|....|....|....|....|....|....|....|
  TUBB2A NP_001060        19    ............................................................................FWEVISDEHGIDPT
H TUBB4A NP_001276052     70    ............................................................................FWEVISDEHGIDPT
U TUBB   NP_001280141     35    ..................................................................QK......IKFWEVISDEHGIDPT
M TUBB3  NP_006077        19    ............................................................................FWEVISDEHGIDPS
A TUBB1  NP_110400        19    ............................................................................FWEMIGEEHGIDLA
N TUBB6  NP_115914        19    ............................................................................FWEVISDEHGIDPA
  TUBB8  NP_817124        19    ............................................................................FWEVISDEHAIDSA
  --------------------------------------------------------------------------------------------------------------------------
  TUBB   Q767L7           17    ..........................................................................AKFWEVISDEHGIDPT
P TUBB2A A0A287AHL4       19    ............................................................................FWEVISDEHGIDPT
I TUBB2B A0A287AEI4       19    ............................................................................FWEVISDEHGIDPT
G TUBB3  A0A5G2R693       19    ............................................................................FWEVISDEHGIDPS
  TUBB4A A0A480UV25       19    ............................................................................FWEVISDEHGIDPT
  TUBB4B A0A287A217       59    RWQRADGAPALRPRAALGPETRRGPLRGRRGHAGGGGGGGGGGGGGRGVEGRAVPGGAGSGLLRRDSAPVRPRLPQFWEVISDEHGIDPT

                                       190       200       210       220       230       240       250       260       270
                                ....|....|....|....|....|....|....|....|....|....|....|....|....|....|....|....|....|....|
  TUBB2A NP_001060        34    GSYHGDSDLQLERINVYYNEAAGNKYVPRAILVDLEPGTMDSVRSGPFGQIFRPDNFVFGQSGAGNNWAKGHYTEGAELVDSVLDVVRKE
H TUBB4A NP_001276052     85    GTYHGDSDLQLERINVYYNEATGGNYVPRAVLVDLEPGTMDSVRSGPFGQIFRPDNFVFGQSGAGNNWAKGHYTEGAELVDAVLDVVRKE
U TUBB   NP_001280141     54    GTYHGDSDLQLDRISVYYNEATGGKYVPRAILVDLEPGTMDSVRSGPFGQIFRPDNFVFGQSGAGNNWAKGHYTEGAELVDSVLDVVRKE
M TUBB3  NP_006077        34    GNYVGDSDLQLERISVYYNEASSHKYVPRAILVDLEPGTMDSVRSGAFGHLFRPDNFIFGQSGAGNNWAKGHYTEGAELVDSVLDVVRKE
A TUBB1  NP_110400        34    GSDRGASALQLERISVYYNEAYGRKYVPRAVLVDLEPGTMDSIRSSKLGALFQPDSFVHGNSGAGNNWAKGHYTEGAELIENVLEVVRHE
N TUBB6  NP_115914        34    GGYVGDSALQLERINVYYNESSSQKYVPRAALVDLEPGTMDSVRSGPFGQLFRPDNFIFGQTGAGNNWAKGHYTEGAELVDAVLDVVRKE
  TUBB8  NP_817124        34    GTYHGDSHLQLERINVYYNEASGGRYVPRAVLVDLEPGTMDSVRSGPFGQVFRPDNFIFGQCGAGNNWAKGHYTEGAELMESVMDVVRKE
  --------------------------------------------------------------------------------------------------------------------------
  TUBB   Q767L7           34    GTYHGDSDLQLDRISVYYNEATGGKYVPRAILVDLEPGTMDSVRSGPFGQIFRPDNFVFGQSGAGNNWAKGHYTEGAELVDSVLDVVRKE
P TUBB2A A0A287AHL4       34    GSYHGDSDLQLERINVYYNEAAGNKYVPRAILVDLEPGTMDSVR.GPFGQIFRPDNFVFGQSGAGNNWAKGHYTEGAELVDSVLDVVRKE
I TUBB2B A0A287AEI4       34    GSYHGDSDLQLERINVYYNEATGNKYVPRAILVDLEPGTMDSVRSGPFGQIFRPDNFVFGQSGAGNNWAKGHYTEGAELVDSVLDVVRKE
G TUBB3  A0A5G2R693       34    GNYVGDSDLQLERISVYYNEASSHKYVPRAILVDLEPGTMDSVRSGAFGHLFRPDNFIFGQSGAGNNWAKGHYTEGAELVDSVLDVVRKE
  TUBB4A A0A480UV25       34    GTYHGDSDLQLERINVYYNEATGGNYVPRAVLVDLEPGTMDSVRSGPFGQIFRPDNFVFGQSGAGNNWAKGHYTEGAELVDAVLDVVRKE
  TUBB4B A0A287A217       149   GTYHGDSDLQLERINVYYNEATGGKYVPRAVLVDLEPGTMDSVRSGPFGQIFRPDNFVFGQSGAGNNWAKGHYTEGAELVDSVLDVVRKE

                                       280       290       300       310       320       330       340       350       360
                                ....|....|....|....|....|....|....|....|....|....|....|....|....|....|....|....|....|....|
  TUBB2A NP_001060        124   SESCDCLQGFQLTHSLGGGTGSGMGTLLISKIREEYPDRIMNTFSVMPSPKVSDTVVEPYNATLSVHQLVENTDETYSIDNEALYDICFR
H TUBB4A NP_001276052     175   AESCDCLQGFQLTHSLGGGTGSGMGTLLISKIREEFPDRIMNTFSVVPSPKVSDTVVEPYNATLSVHQLVENTDETYCIDNEALYDICFR
U TUBB   NP_001280141     144   AESCDCLQGFQLTHSLGGGTGSGMGTLLISKIREEYPDRIMNTFSVVPSPKVSDTVVEPYNATLSVHQLVENTDETYCIDNEALYDICFR
M TUBB3  NP_006077        124   CENCDCLQGFQLTHSLGGGTGSGMGTLLISKVREEYPDRIMNTFSVVPSPKVSDTVVEPYNATLSIHQLVENTDETYCIDNEALYDICFR
A TUBB1  NP_110400        124   SESCDCLQGFQIVHSLGGGTGSGMGTLLMNKIREEYPDRIMNSFSVMPSPKVSDTVVEPYNAVLSIHQLIENADACFCIDNEALYDICFR
N TUBB6  NP_115914        124   CEHCDCLQGFQLTHSLGGGTGSGMGTLLISKIREEFPDRIMNTFSVMPSPKVSDTVVEPYNATLSVHQLVENTDETYCIDNEALYDICFR
  TUBB8  NP_817124        124   AESCDCLQGFQLTHSLGGGTGSGMGTLLLSKIREEYPDRIINTFSILPSPKVSDTVVEPYNATLSVHQLIENADETFCIDNEALYDICSK
  --------------------------------------------------------------------------------------------------------------------------
  TUBB   Q767L7           124   AESCDCLQGFQLTHSLGGGTGSGMGTLLISKIREEYPDRIMNTFSVVPSPKVSDTVVEPYNATLSVHQLVENTDETYCIDNEALYDICFR
P TUBB2A A0A287AHL4       123   SESCDCLQGFQLTHSLGGGTGSGMGTLLISKIREEYPDRIMNTFSVMPSPKVSDTVVEPYNATLSVHQLVENTDETYSIDNEALYDICFR
I TUBB2B A0A287AEI4       124   SESCDCLQGFQLTHSLGGGTGSGMGTLLISKIREEYPDRIMNTFSVMPSPKVSDTVVEPYNATLSVHQLVENTDETYCIDNEALYDICFR
G TUBB3  A0A5G2R693       124   CENCDCLQGFQLTHSLGGGTGSGMGTLLISKVREEYPDRIMNTFSVVPSPKVSDTVVEPYNATLSIHQLVENTDETYCIDNEALYDICFR
  TUBB4A A0A480UV25       124   AESCDCLQGFQLTHSLGGGTGSGMGTLLISKIREEFPDRIMNTFSVVPSPKVSDTVVEPYNATLSVHQLVENTDETYCIDNEALYDICFR
  TUBB4B A0A287A217       239   AESCDCLQGFQLTHSLGGGTGSGMGTLLISKIREEYPDRIMNTFSVVPSPKVSDTVVEPYNATLSVHQLVENTDETYCIDNEALYDICFR

                                       370       380       390       400       410       420       430       440       450
                                ....|....|....|....|....|....|....|....|....|....|....|....|....|....|....|....|....|....|
  TUBB2A NP_001060        214   TLKLTTPTYGDLNHLVSATMSGVTTCLRFPGQLNADLRKLAVNMVPFPRLHFFMPGFAPLTSRGSQQYRALTVPELTQQMFDSKNMMAAC
H TUBB4A NP_001276052     265   TLKLTTPTYGDLNHLVSATMSGVTTCLRFPGQLNADLRKLAVNMVPFPRLHFFMPGFAPLTSRGSQQYRALTVPELTQQMFDAKNMMAAC
U TUBB   NP_001280141     234   TLKLTTPTYGDLNHLVSATMSGVTTCLRFPGQLNADLRKLAVNMVPFPRLHFFMPGFAPLTSRGSQQYRALTVPELTQQVFDAKNMMAAC
M TUBB3  NP_006077        214   TLKLATPTYGDLNHLVSATMSGVTTSLRFPGQLNADLRKLAVNMVPFPRLHFFMPGFAPLTARGSQQYRALTVPELTQQMFDAKNMMAAC
A TUBB1  NP_110400        214   TLKLTTPTYGDLNHLVSLTMSGITTSLRFPGQLNADLRKLAVNMVPFPRLHFFMPGFAPLTAQGSQQYRALSVAELTQQMFDARNTMAAC
N TUBB6  NP_115914        214   TLKLTTPTYGDLNHLVSATMSGVTTSLRFPGQLNADLRKLAVNMVPFPRLHFFMPGFAPLTSRGSQQYRALTVPELTQQMFDARNMMAAC
  TUBB8  NP_817124        214   TLKLPTPTYGDLNHLVSATMSGVTTCLRFPGQLNADLRKLAVNMVPFPRLHFFMPGFAPLTSRGSQQYRALTVAELTQQMFDAKNMMAAC
  --------------------------------------------------------------------------------------------------------------------------
  TUBB   Q767L7           214   TLKLTTPTYGDLNHLVSATMSGVTTCLRFPGQLNADLRKLAVNMVPFPRLHFFMPGFAPLTSRGSQQYRALTVPELTQQVFDAKNMMAAC
P TUBB2A A0A287AHL4       213   TLKLTTPTYGDLNHLVSATMSGVTTCLRFPGQLNADLRKLAVNMVPFPRLHFFMPGFAPLTSRGSQQYRALTVPELTQQMFDSKNMMAAC
I TUBB2B A0A287AEI4       214   TLKLTTPTYGDLNHLVSATMSGVTTCLRFPGQLNADLRKLAVNMVPFPRLHFFMPGFAPLTSRGSQQYRALTVPELTQQMFDSKNMMAAC
G TUBB3  A0A5G2R693       214   TLKLATPTYGDLNHLVSATMSGVTTSLRFPGQLNADLRKLAVNMVPFPRLHFFMPGFAPLTARGSQQYRALTVPELTQQMFDAKNMMAAC
  TUBB4A A0A480UV25       214   TLKLTTPTYGDLNHLVSATMSGVTTCLRFPGQLNADLRKLAVNMVPFPRLHFFMPGFAPLTSRGSQQYRALTVPELTQQMFDAKNMMAAC
  TUBB4B A0A287A217       329   TLKLTTPTYGDLNHLVSATMSGVTTCLRFPGQLNADLRKLAVNMVPFPRLHFFMPGFAPLTSRGSQQYRALTVPELTQQMFDAKNMMAAC
```

```
                                  460       470       480       490       500       510       520       530       540
                           ....|....|....|....|....|....|....|....|....|....|....|....|....|....|....|....|....|....|
  TUBB2A NP_001060      304 DPRHGRYLTVAAIFRGRMSMKEVDEQMLNVQNKNSSYFVEWIPNNVKTAVCDIPPRGLKMSATFIGNSTAIQELFKRISEQFTAMFRRKA
H TUBB4A NP_001276052  355 DPRHGRYLTVAAVFRGRMSMKEVDEQMLSVQSKNSSYFVEWIPNNVKTAVCDIPPRGLKMAATFIGNSTAIQELFKRISEQFTAMFRRKA
U TUBB   NP_001280141  324 DPRHGRYLTVAAVFRGRMSMKEVDEQMLNVQNKNSSYFVEWIPNNVKTAVCDIPPRGLKMAVTFIGNSTAIQELFKRISEQFTAMFRRKA
M TUBB3  NP_006077      304 DPRHGRYLTVATVFRGRMSMKEVDEQMLAIQSKNSSYFVEWIPNNVKVAVCDIPPRGLKMSSTFIGNSTAIQELFKRISEQFTAMFRRKA
A TUBB1  NP_110400      304 DLRRGRYLTVACIFRGKMSTKEVDQQLLSVQTRNSSCFVEWIPNNVKVAVCDIPPRGLSMAATFIGNNTAIQEIFNRVSEHFSAMFKRKA
N TUBB6  NP_115914      304 DPRHGRYLTVATVFRGPMSMKEVDEQMLAIQSKNSSYFVEWIPNNVKVAVCDIPPRGLKMASTFIGNSTAIQELFKRISEQFSAMFRRKA
  TUBB8  NP_817124      304 DPRHGRYLTAAAIFRGRMPMREVDEQMFNIQDKNSSYFADWLPNNVKTAVCDIPPRGLKMSATFIGNNTAIQELFKRVSEQFTAMFRRKA
  TUBB   Q767L7         304 DPRHGRYLTVAAVFRGRMSMKEVDEQMLNVQNKNSSYFVEWIPNNVKTAVCDIPPRGLKMAVTFIGNSTAIQELFKRISEQFTAMFRRKA
P TUBB2A A0A287AHL4     303 DPRHGRYLTVAAIFRGRMSMKEVDEQMLNVQNKNSSYFVEWIPNNVKTAVCDIPPRGLKMSATFIGNSTAIQELFKRISEQFTAMFRRKA
I TUBB2B A0A287AEI4     304 DPRHGRYLTVAAIFRGRMSMKEVDEQMLNVQNKNSSYFVEWIPNNVKTAVCDIPPRGLKMSATFIGNSTAIQELFKRISEQFTAMFRRKA
G TUBB3  A0A5G2R693     304 DPRHGRYLTVATVFRGRMSMKEVDEQMLAIQSKNSSYFVEWIPNNVKVAVCDIPPRGLPALQHERPGVRVPAVPGRHGRRGGRDVRGRRG
  TUBB4A A0A480UV25     304 DPRHGRYLTVAAVFRGRMSMKEVDEQMLSVQSKNSSYFVEWIPNNVKTAVCDIPPRGLKMAATFIGNSTAIQELFKRISEQFTAMFRRKA
  TUBB4B A0A287A217     419 DPRHGRYLTVAAVFRGRMSMKEVDEQMLNVQNKNSSYFVEWIPNNVKTAVCDIPPRGLKMSATFIGNSTAIQELFKRISEQFTAMFRRKA
                                                                Detected labeled peptide (major)
```

```
                                  550       560       570       580       590       600       610       620       630
                           ....|....|....|....|....|....|....|....|....|....|....|....|....|....|....|....|....|....|
  TUBB2A NP_001060      394 FLHWYT........GEGMDEMEFTEAESNMNDLVSEYQQYQDATADEQGEFEEEEGEDEA..............................
H TUBB4A NP_001276052  445 FLHWYT........GEGMDEMEFTEAESNMNDLVSEYQQYQDATAEE.GEFEEEAEEEVA..............................
U TUBB   NP_001280141  414 FLHWYT........GEGMDEMEFTEAESNMNDLVSEYQQYQDATAEEEEDFGEEAEEEA...............................
M TUBB3  NP_006077      394 FLHWYT........GEGMDEMEFTEAESNMNDLVSEYQQYQDATAEEEGEMYEDDEEESEAQGPK.........................
A TUBB1  NP_110400      394 FVHWYT........SEGMDINEFGEAENNIHDLVSEYQQFQDAKAVLEEDEEVTEEAEMEPEDKGH........................
N TUBB6  NP_115914      394 FLHWFT........GEGMDEMEFTEAESNMNDLVSEYQQYQDATANDGEEAFEDEEEEIDG.............................
  TUBB8  NP_817124      394 FLHWYT........GEGMDEMEFTEAESNMNDLVSEYQQYQDATAEEEEDEEYAEEEVA...............................
  TUBB   Q767L7         394 FLHWYT........GEGMDEMEFTEAESNMNDLVSEYQQYQDATAEEEEDFGEEAEEEA...............................
P TUBB2A A0A287AHL4     393 FLHWYT........GEGMDEMEFTEAESNMNDLVSEYQQYQDATADEQGEFEEEEGEDEA..............................
I TUBB2B A0A287AEI4     394 FLHWYT........GEGMDEMEFTEAESNMNDLVSEEWKASSCVPSSYIFLPP..GQPF...............................
G TUBB3  A0A5G2R693     394 GVRGPGPQVRRPGGGRGTARTQRSAPSRVAPDTARPRPTRPERVAPGPPSVRPAVFAASLPRPSPPSHPPPSVISVVCGPLCLLYCPLQA
  TUBB4A A0A480UV25     394 FLHWYT........GEGMDEMEFTEAESNMNDLVSEYQQYQDATAEE.GEFEEEAEEEVA..............................
  TUBB4B A0A287A217     509 FLHWYT........GEGMDEMEFTEAESNMNDLVSEYQQYQDATAEEEGEFEEEAEEEVA..............................
```

Supplementary Figure 11. Sequence alignment of β-tubulin isotypes from human (Homo sapiens) and pig (Sus scrofa). Labeled peptides detected in the tubulin sample are marked green.

```
                                     10        20        30        40        50        60        70        80        90
                             ....|....|....|....|....|....|....|....|....|....|....|....|....|....|....|....|....|....|
H TUBA2  ABD72607          1 ......................................................................MRECISIHVGQAGVQIGNAC
U TUBA1A NP_001257328      1 ......................................................................MRECISIHVGQAGVQIGNAC
M TUBA1C NP_001290043      1 MTAWFNGVEELGRDECFVCLELDPSNGSFLNAKLYISNWILIYFTRTLQLPAPFNTCTSSKSRGEPGCDSKRECISIHVGQAGVQIGNAC
A TUBA4A NP_005991         1 ......................................................................MRECISVHVGQAGVQMGNAC
N TUBA1B NP_006073         1 ......................................................................MRECISIHVGQAGVQIGNAC
  TUBA8  NP_061816         1 ......................................................................MRECISVHVGQAGVQIGNAC
  TUBA1A P02550            1 ......................................................................MRECISIHVGQAGVQIGNAC
P TUBA3C F1RK98            1 ...........................................................LFWASHICSGPQRECISIHVGQAGVQIGNAC
I TUBA4A A0A286ZNY1        1 ......................................................................MRECISVHVGQAGVQMGNAC
G TUBA8A A0A287BMB7        1 ......................................................................MRECISVHVGQAGVQIGNAC

                                    100       110       120       130       140       150       160       170       180
                             ....|....|....|....|....|....|....|....|....|....|....|....|....|....|....|....|....|....|
H TUBA2  ABD72607         21 WELYCLEHGIQPDGQMPSDKTIGGGDDSFNTFFSETGAGKHVPRAVFVDLEPTVVDEVRTGTYRQLFHPEQLITGKEDAANNYARGHYTI
U TUBA1A NP_001257328     21 WELYCLEHGIQPDGQMPSDKTIGGGDDSFNTFFSETGAGKHVPRAVFVDLEPTVIDEVRTGTYRQLFHPEQLITGKEDAANNYARGHYTI
M TUBA1C NP_001290043     91 WELYCLEHGIQPDGQMPSDKTIGGGDDSFNTFFSETGAGKHVPRAVFVDLEPTVIDEVRTGTYRQLFHPEQLITGKEDAANNYARGHYTI
A TUBA4A NP_005991        21 WELYCLEHGIQPDGQMPSDKTIGGGDDSFTTFFCETGAGKHVPRAVFVDLEPTVIDEIRNGPYRQLFHPEQLITGKEDAANNYARGHYTI
N TUBA1B NP_006073        21 WELYCLEHGIQPDGQMPSDKTIGGGDDSFNTFFSETGAGKHVPRAVFVDLEPTVIDEVRTGTYRQLFHPEQLITGKEDAANNYARGHYTI
  TUBA8  NP_061816        21 WELFCLEHGIQADGTFDAQASKINDDDSFTTFFSETGNGKHVPRAVMIDLEPTVVDEVRAGTYRQLFHPEQLITGKEDAANNYARGHYTV
  TUBA1A P02550           21 WELYCLEHGIQPDGQMPSDKTIGGGDDSFNTFFSETGAGKHVPRAVFVDLEPTVIDEVRTGTYRQLFHPEQLITGKEDAANNYARGHYTI
P TUBA3C F1RK98           32 WELYCLEHGIQPDGQMPSDKTIGGGDDSFNTFFSETGAGKHVPRAVFVDLEPTVVDEVRTGTYRQLFHPEQLITGKEDAANNYARGHYTI
I TUBA4A A0A286ZNY1       21 WELYCLEHGIQPDGQMPSDKTIGGGDDSFTTFFCETGAGKHVPRAVFVDLEPTVIDEIRNGPYRQLFHPEQLITGKEDAANNYARGHYTI
G TUBA8A A0A287BMB7       21 WELFCLEHGIQADGTFGAQASKLHDDDSFTTFFSETGNGKHVPRAVMVDLEPTVVDEVRAGTYRQLFHPEQLITGKEDAANNYARGHYTV

                                    190       200       210       220       230       240       250       260       270
                             ....|....|....|....|....|....|....|....|....|....|....|....|....|....|....|....|....|....|
H TUBA2  ABD72607        111 GKEIVDLVLDRIRKLADLCTGLQGFLIFHSFGGGTGSGFASLLMERLSVDYGKKSKLEFAIYPAPQVSTAVVEPYNSILTTHTTLEHSDC
U TUBA1A NP_001257328   111 GKEIIDLVLDRIRKLADQCTGLQGFLVFHSFGGGTGSGFTSLLMERLSVDYGKKSKLEFSIYPAPQVSTAVVEPYNSILTTHTTLEHSDC
M TUBA1C NP_001290043   181 GKEIIDLVLDRIRKLADQCTGLQGFLVFHSFGGGTGSGFTSLLMERLSVDYGKKSKLEFSIYPAPQVSTAVVEPYNSILTTHTTLEHSDC
A TUBA4A NP_005991       111 GKEIIDPVLDRIRKLSDQCTGLQGFLVFHSFGGGTGSGFTSLLMERLSVDYGKKSKLEFSIYPAPQVSTAVVEPYNSILTTHTTLEHSDC
N TUBA1B NP_006073       111 GKEIIDLVLDRIRKLADQCTGLQGFLVFHSFGGGTGSGFTSLLMERLSVDYGKKSKLEFSIYPAPQVSTAVVEPYNSILTTHTTLEHSDC
  TUBA8  NP_061816       111 GKESIDLVLDRIRKLTDACSGLQGFLIFHSFGGGTGSGFTSLLMERLSLDYGKKSKLEFAIYPAPQVSTAVVEPYNSILTTHTTLEHSDC
  TUBA1A P02550          111 GKEIIDLVLDRIRKLADQCTGLQGFSVFHSFGGGTGSGFTSLLMERLSVDYGKKSKLEFSIYPAPQVSTAVVEPYNSILTTHTTLEHSDC
P TUBA3C F1RK98          122 GKEIVDLVLDRIRKLADLCTGLQGFLIFHSFGGGTGSGFASLLMERLSVDYGKKSKLEFAIYPAPQVSTAVVEPYNSILTTHTTLEHSDC
I TUBA4A A0A286ZNY1      111 GKEIIDPVLDRIRKLSDQCTGLQGFLVFHSFGGGTGSGFTSLLMERLSVDYGKKSKLEFSIYPAPQVSTAVVEPYNSILTTHTTLEHSDC
G TUBA8A A0A287BMB7      111 GKESIDLVLDRIRKLTDACSGLQGFLIFHSFGGGTGSGFTSLLMERLSLDYGKKSKLEFAIYPAPQVSTAVVEPYNSILTTHTTLEHSDC

                                    280       290       300       310       320       330       340       350       360
                             ....|....|....|....|....|....|....|....|....|....|....|....|....|....|....|....|....|....|
H TUBA2  ABD72607        201 AFMVDNEAIYDICRRNLDIERPTYTNLNRLIGQIVSSITASLRFDGALNVDLTEFQTNLVPYPRIHFPLATYAPVISAEKAYHEQLSVAE
U TUBA1A NP_001257328   201 AFMVDNEAIYDICRRNLDIERPTYTNLNRLIGQIVSSITASLRFDGALNVDLTEFQTNLVPYPRIHFPLATYAPVISAEKAYHEQLSVAE
M TUBA1C NP_001290043   271 AFMVDNEAIYDICRRNLDIERPTYTNLNRLISQIVSSITASLRFDGALNVDLTEFQTNLVPYPRIHFPLATYAPVISAEKAYHEQLTVAE
A TUBA4A NP_005991       201 AFMVDNEAIYDICRRNLDIERPTYTNLNRLISQIVSSITASLRFDGALNVDLTEFQTNLVPYPRIHFPLATYAPVISAEKAYHEQLSVAE
N TUBA1B NP_006073       201 AFMVDNEAIYDICRRNLDIERPTYTNLNRLISQIVSSITASLRFDGALNVDLTEFQTNLVPYPRIHFPLATYAPVISAEKAYHEQLSVAE
  TUBA8  NP_061816       201 AFMVDNEAIYDICRRNLDIERPTYTNLNRLISQIVSSITASLRFDGALNVDLTEFQTNLVPYPRIHFPLVTYAPIISAEKAYHEQLSVAE
  TUBA1A P02550          201 AFMVDNEAIYDICRRNLDIERPTYTNLNRLIGQIVSSITASLRFDGALNVDLTEFQTNLVPYPRAHFPLATYAPVISAEKAYHEQLSVAE
P TUBA3C F1RK98          212 AFMVDNEAIYDICRRNLDIERPTYTNLNRLIGQIVSSITASLRFDGALNVDLTEFQTNLVPYPRIHFPLATYAPVISAEKAYHEQLSVAE
I TUBA4A A0A286ZNY1      201 AFMVDNEAIYDICRRNLDIERPTYTNLNRLISQIVSSITASLRFDGALNVDLTEFQTNLVPYPRIHFPLATYAPVISAEKAYHEQLSVAE
G TUBA8A A0A287BMB7      201 AFMVDNEAIYDICRRNLDIERPTYTNLNRLISQIVSSITASLRFDGALNVDLTEFQTNLVPYPRIHFPLVTYAPIISAEKAYHEQLSVAE

                                    370       380       390       400       410       420       430       440       450
                             ....|....|....|....|....|....|....|....|....|....|....|....|....|....|....|....|....|....|
H TUBA2  ABD72607        291 ITNACFEPANQMVKCDPRHGKYMACCMLYRGDVVPKDVNAAIATIKTKRTIQFVDWCPTGFKVGINYQPPTVVPGGDLAKVQRAVCMLSN
U TUBA1A NP_001257328   291 ITNACFEPANQMVKCDPRHGKYMACCLLYRGDVVPKDVNAAIATIKTKRTIQFVDWCPTGFKVGINYQPPTVVPGGDLAKVQRAVCMLSN
M TUBA1C NP_001290043   361 ITNACFEPANQMVKCDPRHGKYMACCLLYRGDVVPKDVNAAIATIKTKRTIQFVDWCPTGFKVGINYQPPTVVPGGDLAKVQRAVCMLSN
A TUBA4A NP_005991       291 ITNACFEPANQMVKCDPRHGKYMACCLLYRGDVVPKDVNAAIAAIKTKRSIQFVDWCPTGFKVGINYQPPTVVPGGDLAKVQRAVCMLSN
N TUBA1B NP_006073       291 ITNACFEPANQMVKCDPRHGKYMACCLLYRGDVVPKDVNAAIATIKTKRSIQFVDWCPTGFKVGINYQPPTVVPGGDLAKVQRAVCMLSN
  TUBA8  NP_061816       291 ITSSCFEPNSQMVKCDPRHGKYMACCMLYRGDVVPKDVNVAIAAIKTKRTIQFVDWCPTGFKVGINYQPPTVVPGGDLAKVQRAVCMLSN
  TUBA1A P02550          291 ITNACFEPANQMVKCDPRHGKYMACCLLYRGDVVPKDVNAAIATIKTKRTIQFVDWCPTGFKVGINYEPPTVVPGGDLAKVQRAVCMLSN
P TUBA3C F1RK98          302 ITNACFEPANQMVKCDPRHGKYMACCMLYRGDVVPKDVNAAIATIKTKRTIQFVDWCPTGFKVGINYQPPTVVPGGDLAKVQRAVCMLSN
I TUBA4A A0A286ZNY1      291 ITNACFEPANQMVKCDPRHGKYMACCLLYRGDVVPKDVNAAIAAIKTKRSIQFVDWCPTGFKVGINYQPPTVVPGGDLAKVQRAVCMLSN
G TUBA8A A0A287BMB7      291 ITSSCFEPNSQMVKCDPRHGKYMACCMLYRGDVVPKDVNVAIAAIKTKRTIQFVDWCPTGFKVGINYQPPTVVPGGDLAKVQRAVCMLSN
                                                                                        Detected labeled peptides (minor)

                                    460       470       480       490       500       510       520
                             ....|....|....|....|....|....|....|....|....|....|....|....|....|....|.
H TUBA2  ABD72607        381 TTAIAEAWARLDHKFDLMYAKRAFVHWYVGEGMEEGEFSEAREDLAALEKDYEEVGVDSVEAEAE.EGEEY
U TUBA1A NP_001257328   381 TTAIAEAWARLDHKFDLMYAKRAFVHWYVGEGMEEGEFSEAREDMAALEKDYEEVGVDSVEGEGEEEGEEY
M TUBA1C NP_001290043   451 TTAVAEAWARLDHKFDLMYAKRAFVHWYVGEGMEEGEFSEAREDMAALEKDYEEVGADSADGEDE..GEEY
A TUBA4A NP_005991       381 TTAIAEAWARLDHKFDLMYAKRAFVHWYVGEGMEEGEFSEAREDMAALEKDYEEVGIDSYEDEDE..GEE.
N TUBA1B NP_006073       381 TTAIAEAWARLDHKFDLMYAKRAFVHWYVGEGMEEGEFSEAREDMAALEKDYEEVGVDSVEGEGEEEGEEY
  TUBA8  NP_061816       381 TTAIAEAWARLDHKFDLMYAKRAFVHWYVGEGMEEGEFSEAREDLAALEKDYEEVGTDSFEEENE..GEEF
  TUBA1A P02550          381 TTAIAEAWARLDHKFDLMYAKRAFVHWYVGEGMEEGEFSEAREDMAALEKDYEEVGVDSVEGEGEEEGEEY
P TUBA3C F1RK98          392 TTAIAEAWARLDHKFDLMYAKRAFVHWYVGEGMEEGEFSEAREDLAALEKDYEEVGVDSVEAEAE.EGEEY
I TUBA4A A0A286ZNY1      381 TTAIAEAWARLDHKFDLMYAKRAFVHWYVGEGMEEGEFSEAREDMAALEKDYEEVGIDSYEDEDE..GEE.
G TUBA8A A0A287BMB7      381 TTAIAEAWARLDHKFDLMYAKRAFVHWYVGEGMEEGEFSEAREDLAALEKDYEEVGTDSFEEENE..GEEF
```

Supplementary Figure 12. Sequence alignment of α-tubulin isotypes from human (Homo sapiens) and pig (Sus scrofa). Labeled peptides detected in the tubulin sample are marked green.

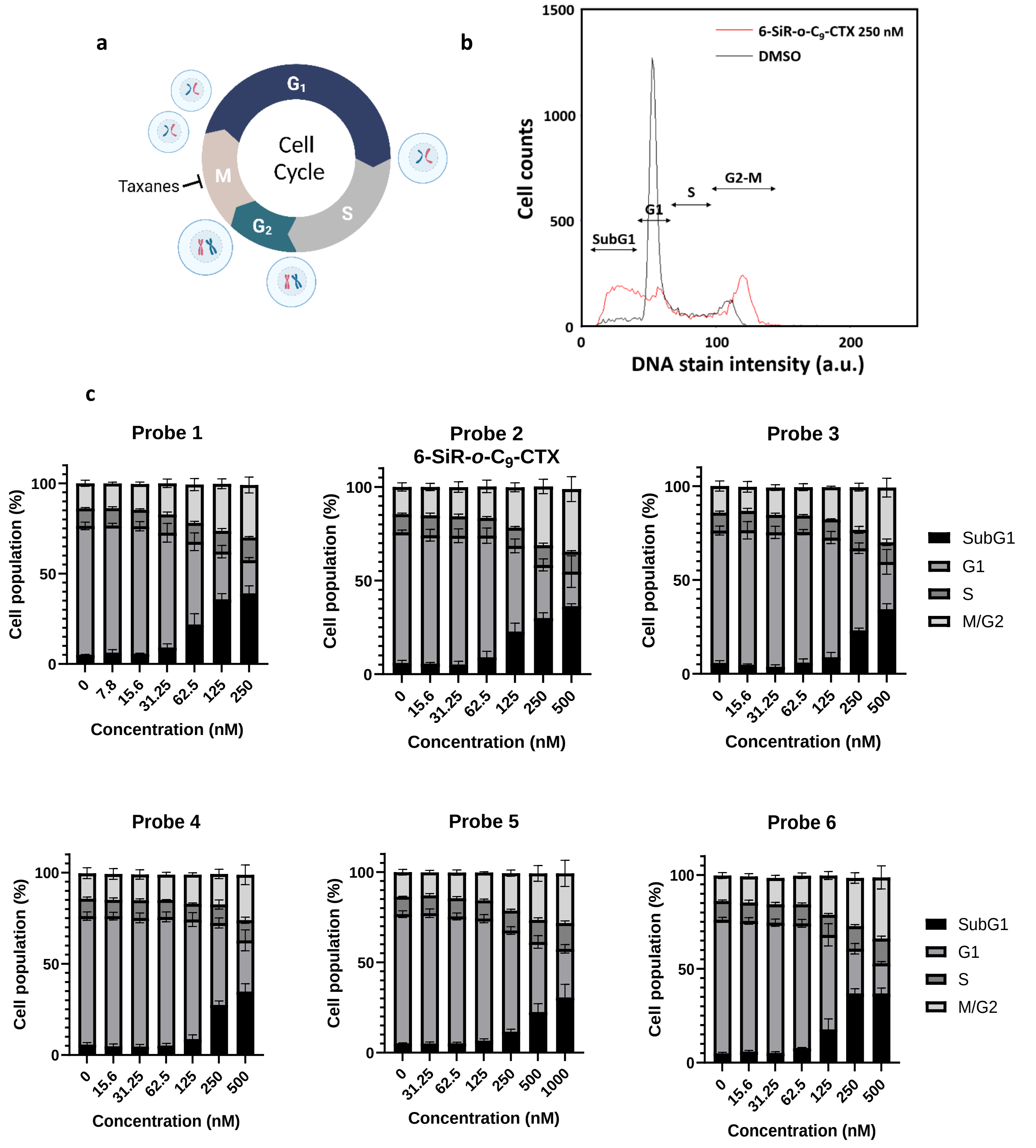

Supplementary Figure 13. Cell cycle perturbation induced by covalent tubulin probes. (**a**) Cytotoxicity of taxanes results from the inhibition of the cycle at the stage of mitosis. Created with BioRender.com. (**b**) Representative histogram of DNA content distribution in HeLa cells treated with DMSO or 250 nM of **6-SiR-*o*-C$_9$-CTX** for 24h. The cell cycle phases are identified by the amount of DNA per cell. (**c**) Cytotoxicity measurements of tubulin probes. Experimental data are averages of three independent experiments (N=3) and presented as means with standard deviations. Cell cycle diagram created with BioRender.com.

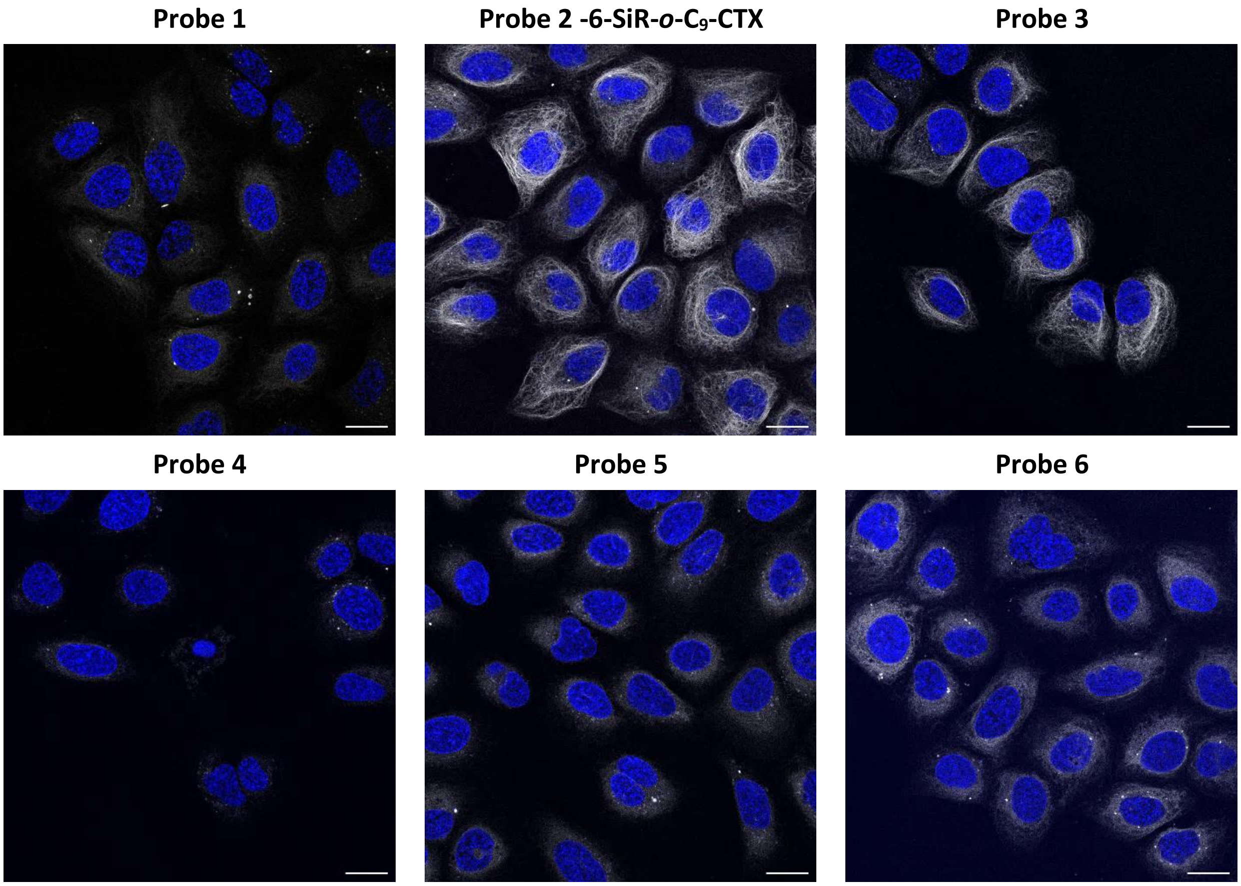

Supplementary Figure 14. Live U-2 OS cells stained with probes (1 µM in OptiMEM), verapamil (10 µM) and Hoechst 33342 (1 µg/mL) for 4h. Confocal microscopy images were acquired using LEICA SP8. Gray channel (λ_{ex}= 633 nm, λ_{em}= 650-710 nm) corresponds to probe staining and blue channel (λ_{ex}= 405 nm, λ_{em}= 415-480 nm) corresponds to Hoechst 33342 staining. Scale bar = 20 µm.

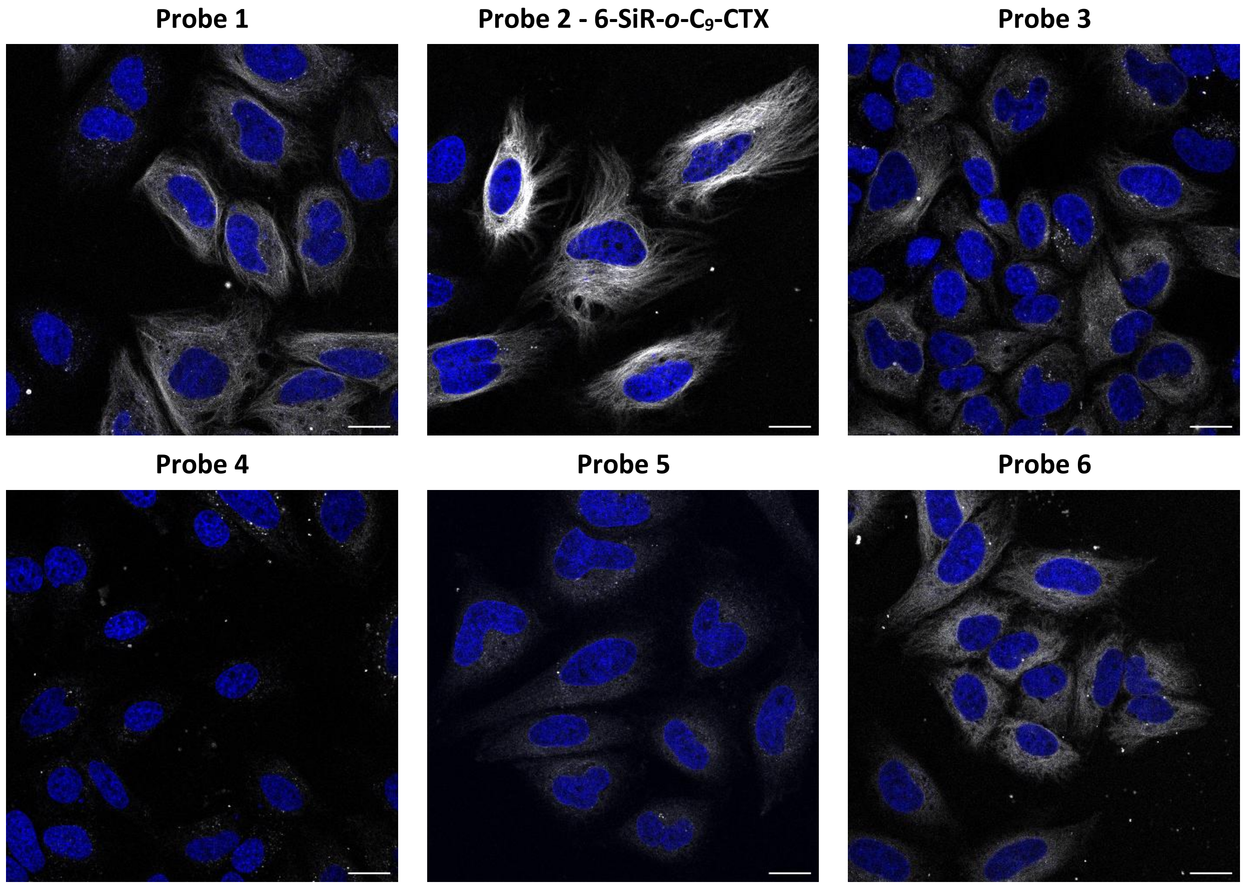

Supplementary Figure 15. Live HeLa CCL cells stained with probes (1 µM in OptiMEM) and Hoechst 33342 (1 µg/mL) for 4h. Confocal microscopy images were acquired using LEICA SP8. Gray channel (λ_{ex}= 633 nm, λ_{em}= 650-710 nm) corresponds to probe staining and blue channel (λ_{ex}= 405 nm, λ_{em}= 415-480 nm) corresponds to Hoechst 33342 staining. Scale bar = 20 µm.

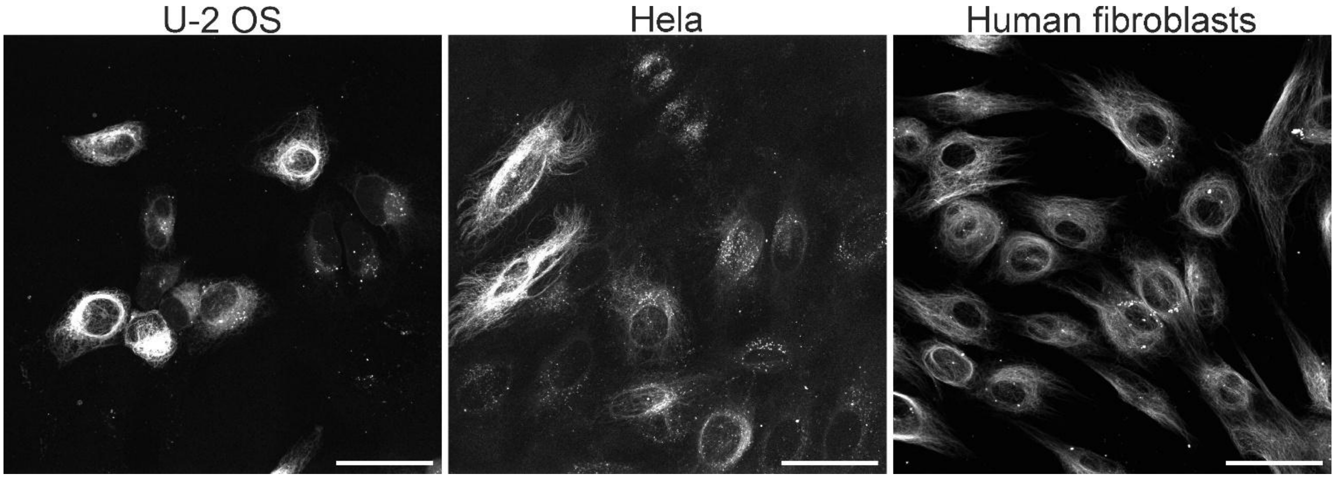

Supplementary Figure 16. Live U-2 OS, HeLa CCL cells and human fibroblasts stained with 1µM 6-SiR-o-C_9-CTX in OptiMEM for 4h after extensive washing procedure. Note off-target staining visible as dotted structures. Images presented as maximum intensity projections of the acquired Z-stacks. Confocal microscopy images were acquired using Visitron spinning disk confocal microscope. Scale bar = 50 µm.

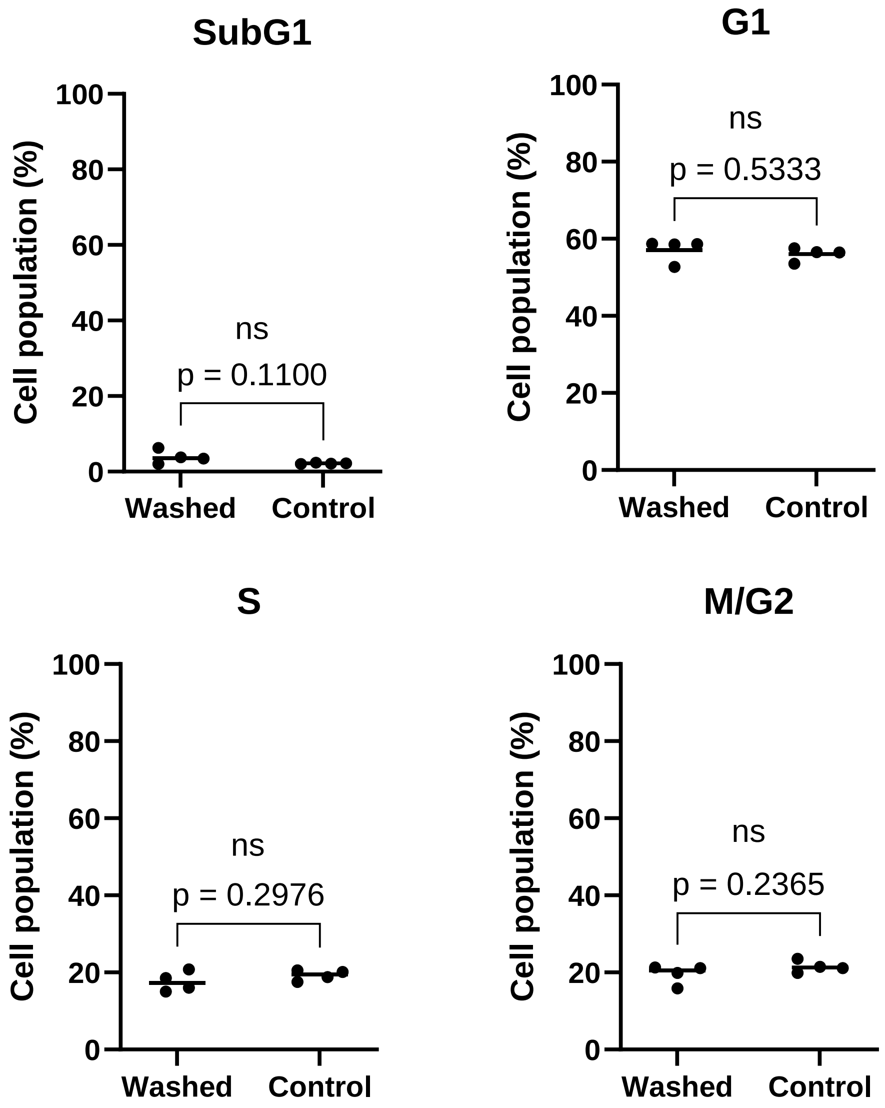

Supplementary Figure 17. Statistical analysis associated with the cell cycle analysis of washing experiment. Data from four different experiments (N=4). The line corresponds to the mean and each dot corresponds to the result of one single experiment. Unpaired t test was performed between the 2 conditions.

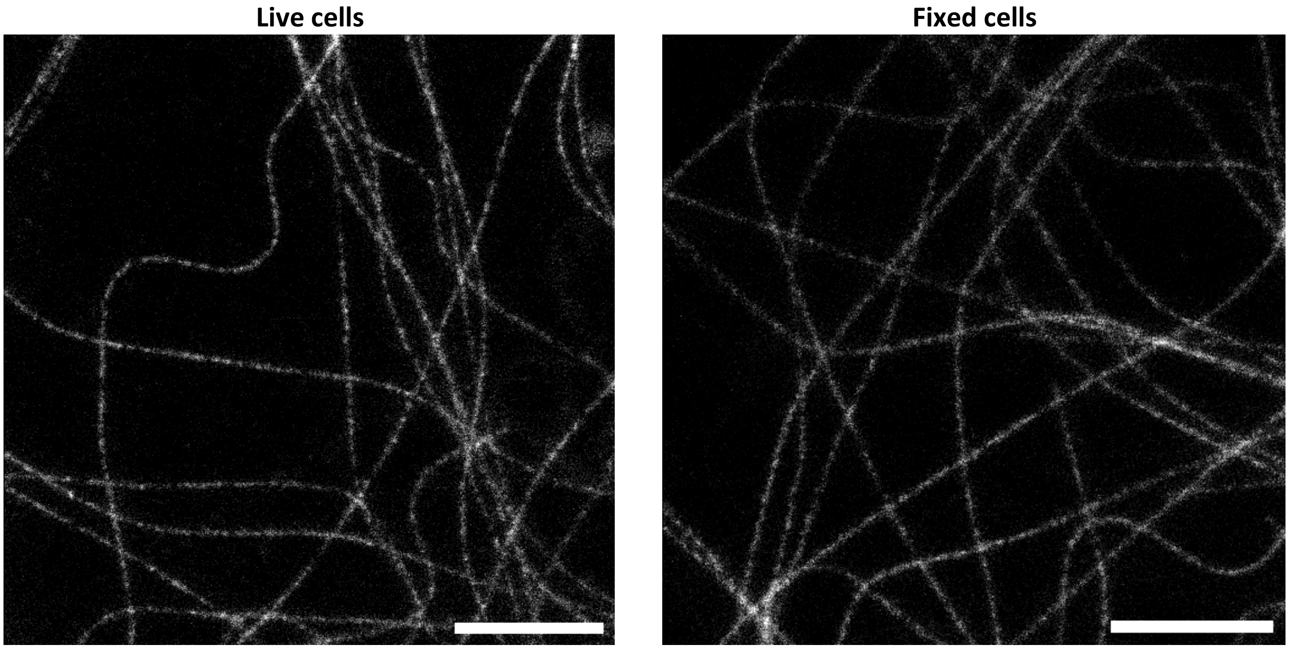

Supplementary Figure 18. Representative images used to measure apparent microtubule FWHM. Human dermal fibroblasts cells incubated with **6-SiR-*o*-C_9-CTX** (1 µM in OptiMEM) for 4h, imaged live or after fixation with glutaraldehyde. Images acquired with Abberior Expert Line. Scale bar = 2 µm.

Supplementary Tables

	$\lambda_{abs,max}$ (nm)	$\lambda_{em,max}$ (nm)	QY PBS+0.1 % SDS (%)	Fluorescence lifetime PBS+0.1 % SDS (ns)	Percentage dye open Tubulin/PBS+0.1% SDS (%)	Fl increase Tubulin/PBS
**Probe 1**	652	672	59.1 ± 0.5	3.94 ± 0.01	40.0 ± 2.6	7.0 ± 2.2
**Probe 2 – 6-SiR-*o*-C_9-CTX**			59.3 ± 1.4	3.93 ± 0.01	49.0 ± 10.8	13.7 ± 1.3
**Probe 3**			60.3 ± 3.2	3.94 ± 0.00	33.3 ± 5.0	8.8 ± 2.8
**Probe 4**			59.4 ± 1.3	3.95 ± 0.01	14.3 ± 8.0	4.1 ± 2.4
**Probe 5**			59.4 ± 1.1	3.93 ± 0.02	25.7 ± 9.0	1.3 ± 0.5
**Probe 6**			58.9 ± 1.2	3.90 ± 0.01	17.0 ± 10.1	5.6 ± 2.9

Supplementary Table 1. Photophysical properties of the probes (1-6). Experimental data are averages of three independent experiments (N=3) and presented as means with standard deviations.

Labeled peptide	Tubulin isotypes and isoforms	Total precursor abundance (MS1 intensity)
TAVcDIPPR	TUBB2A, TUBB2B, TUBB4A, TUBB4B, TUBB	$2{,}97.10^{6}$
VAVcDIPPR	TUBB3	$3{,}59.10^{5}$
EIVHIQAGQcGNQIGAK	TUBB2A, TUBB2B, TUBB, TUBB3	$3{,}57.10^{5}$
EIVHLQAGQcGNQIGAK	TUBB4A, TUBB4B	
SIQFVDWcPTGFK	LOC100158003, LOC100127131, TUBA4A	$1{,}59.10^{5}$
AVcmLSNTTAIAEAWAR	LOC100158003, LOC100127131, TUBA3C, TUBA1A, TUBA4A, TUBA8	$1{,}25.10^{5}$
AVcMLSNTTAIAEAWAR	LOC100158003, LOC100127131, TUBA3C, TUBA1A, TUBA4A, TUBA8	$6{,}63.10^{4}$

Supplementary Table 2. Identified labeled peptides in the sample. Reacted Cys residue is marked in red. m is an oxidized methionine.

Probe name	Cytotoxicity threshold (nM)
**Probe 1**	31.25
**Probe 2 – 6-SiR-*o*-C_9-CTX**	125
**Probe 3**	250
**Probe 4**	250
**Probe 5**	250
**Probe 6**	125

Supplementary Table 3. Cytotoxicity threshold of the probes (1-6). The indicated probe concentration represents the lowest concentration tested, at which a cytotoxicity effect was observed.

Figure	Probe	Cell line	Microscope	Objective	Excitation (%)/ STED (%)	Pixel dwell time (µs)	Pixel size (nm)	Emission (nm)	Comment
Fig. 3a	1,2,3,4,5,6	H. fibro	Leica TCS SP8	63x 1.40	633 (1)	0.6	90	650-710	
Supplementary Fig. 13	1,2,3,4,5,6	U-2 OS							
Supplementary Fig. 14	1,2,3,4,5,6	HeLa CCL							
Fig. 3b	2	U-2 OS<br>HeLa CCL							
Fig. 4b	2, SiR-CTX	U-2 OS							
Fig. 5a	2	HeLa CCL (live)	Abberior Expert Line	Olympus 100x 1.40	640 (10)/775(25)	10	20	650-720	
		HeLa CCL (fixed)			640 (5)/775(60)	4	20	650-720	Line accum.: 2
		U-2 OS (live)			640 (10)/775(25)	10	20	650-720	
		U-2 OS (fixed)			640 (10)/775(70)	4	20	650-720	Line accum.: 2
		H. fibro (live)			640 (17)/ 775(40)	3	20	650-720	
Fig. 5b	2	H. fibro (fixed)	Abberior Expert Line	Olympus 100x 1.40	640 (10)/775(70)	4	20	650-720	Line accum.: 2
Fig. 5d	2	H. fibro (fixed)	Abberior Expert Line	Olympus 100x 1.40	640 (10)/775(75)	1	15	650-720	Line accum.: 3
Supplementary 16	2	HeLa CCL (live)<br>U-2 OS (live)<br>H. fibro (fixed)	Visitron spinning disk confocal	Nikon CFI Plan Apo 60x 1.40	640 (20)	200 ms exposure	110	665 long pass filter	Z-stack 71 planes × 200nm
Supplementary 17	2	H. fibro (live)	Abberior Expert Line	Olympus 100x 1.40	640 (15)/775(75)	1	15	650-720	Line accum.: 3
Movie 2	2	H. fibro (live)	Abberior Facility Line	Olympus 60x 1.40	640 (5)/775(10)	5	30	660-750	One frame every 25 sec

Supplementary Table 4. Acquisition parameters for microscopy images

Supplementary Methods

Determination of absolute quantum yields

All reported absolute fluorescence quantum yield values (Φ) were measured using a Quantaurus-QY spectrometer (model C11374-01, Hamamatsu Photonics). This instrument uses an integrating sphere to determine photons absorbed and emitted by a sample. Measurements were carried out using dilute samples in air saturated solvents at 25°C at concentrations ranging from 10^{-6} to 10^{-7} M (A < 0.1 as indicated in the user's manual) and by using 3 mL quartz cuvettes (Hamamatsu Photonics Art. No. A10095-02) provided by the instrument supplier. The fluorescence quantum yields were measured in PBS buffer containing 0.1% SDS and estimated using Quantaurus QY software (version 3.8.1). Reported values are averages (N = 3) with standard deviation.

Determination of fluorescence lifetimes

The fluorescence decay characteristics of the solution samples in PBS containing 0.1% SDS at concentrations ranging from 10^{-6} to 10^{-7} M were recorded using a fluorescence lifetime measurement system (Quantaurus-Tau, Hamamatsu Photonics) in 3 mL high performance quartz glass cuvettes (Hellma Analytics Art. No. 101-10-K-40). The decay profile was registered for 53 ns interval after excitation and the experiment was continued until 10 000 peak count was reached. The instrument response function was obtained by using diluted LUDOX® TM-50 colloidal silica (Sigma Aldrich, # 420778). The analysis of the obtained fluorescence decay profile was performed using the instrument software (Hamamatsu U11487 version 3.0.0.80). Reported values are averages (N = 3) with standard deviation.

Measurements of absorbance spectra in 1,4-dioxane–water mixtures

Measurements of the absorbance changes in 1,4-dioxane–water mixtures were performed by pipetting 2 µL stock solutions of probes (0.5 mM in DMSO) into a 96-well plate made from propylene (Corning 3364). To the wells going from right to left 250 µL of 1,4-dioxane–water mixtures containing 100%, 90%, 80%, 70%, 60%, 50%, 40%, 30%, 20%, 10% or 0% 1,4-dioxane was added (alternatively, mixtures with 0.3% SDS are used). After incubation for 1 hour at room temperature, absorption spectra of solutions in each well was recorded from 320 nm to 850 nm with wavelength step size of 1 nm on a multiwell plate reader Spark® 20 M (Tecan) using TECAN Spark 1.2.20 software. The background absorption of the glass bottom plate was measured in wells containing only the 1,4-dioxane–water mixture (or mix containing 0.3% SDS) with a same amount of DMSO and subtracted from the spectra of the samples.

Data points from the titration without addition of SDS were fitted to bell-shaped dose response curve described by following equation (1):

$$A = A_0 + \frac{A_{max} - A_0}{1+\left(\frac{D_{50}}{d}\right)^{Hill_1}} - \frac{A_{max} - A_0}{1+\left(\frac{A_{50}}{d}\right)^{Hill_2}} \qquad (1)$$

where A_0 – absorbance at λmax at d = 0, A_{max} – the highest reached absorbance at λ_{max} during the titration experiment. d – dielectric constant of 1,4-dioxane-water mixture at a given point, $Hill_1$ - Hill slope coefficient determining the steepness of the ascending dose-response curve part, $Hill_2$ - Hill slope coefficient determining the steepness of the declining dose-response curve part, D_{50} - corresponds to d value that provokes half of the absorbance amplitude (A_{max}-A_0) in the ascending dose-response curve part, A_{50} - corresponds to d value that provokes half of the absorbance amplitude (A_{max}-A_0) in the declining dose-response curve part.

D50 value was obtained by fitting data points in the presence of 0.3 % SDS to dose-response equation EC50 (2) as implemented in GraphPad 6.0 software:

$$A = A_0 + \frac{A_{max} - A_0}{\left(1+\left(\frac{D_{50}}{d}\right)^{Hill}\right)} \quad (2)$$

where A_0 – absorbance at λmax at d = 0, A_{max} – the highest reached absorbance at λ_{max}. d – dielectric constant of 1,4-dioxane-water mixture at a given point, *Hill* - Hill slope coefficient determining the steepness of a dose-response curve, D_{50} - corresponds to d value that provokes half of the absorbance amplitude (A_{max}-A_0).

Maintenance and preparation of the cells

Human primary dermal fibroblasts (Lonza, #CC-2511) were cultured in high-glucose DMEM (Thermo Fisher, #31053044) with 10% FBS (Thermo Fisher, #10082147) supplemented with 1 mM Sodium pyruvate (Sigma, #S8636), 1% GlutaMax (Thermo Fisher, #35050038) and 1% Penicillin-Streptomycin (Sigma, #P0781) in a humidified 5% CO_2 incubator at 37 °C. The cells were split every 3-4 days or at confluence.

HeLa (ATCC, CCL-2) cells were cultured in high-glucose DMEM (Thermo Fisher, #31966047) with 10% FBS (BioSELL, #S0615) supplemented with 1% Penicillin-Streptomycin (Sigma, #P0781) in a humidified 5% CO_2 incubator at 37 °C. The cells were split every 3-4 days or at confluence.

U-2 OS cells (ATCC, HTB-96) were cultured in McCoys 5A medium (Thermo Fisher, #16600082) with 10% FBS (BioSELL, #S0615) supplemented with 1 mM Sodium pyruvate (Sigma, #S8636) and 1% of Penicillin-Streptomycin (Sigma #P0781) in a humidified 5% CO_2 incubator at 37 °C. The cells were split every 3-4 days or at confluence.

Confocal and STED microscopy experiments were performed using µ-Slide 8 Well Glass Bottom dishes (Ibidi, #80827).

Chemical Synthesis of the building blocks

Reagents were purchased as reagent-grade (from Sigma-Aldrich, BLD Pharm, Enamine, abcr, TCI or Aaron Chem) and used without further purification. 6-SiR-CO_2H was synthesized according to published procedure.[2] Flash chromatographies were carried out on Biotage® Selekt with Biotage® Sfär Silica HC (20 µm) columns.

NMR spectra were recorded at 25 °C with an Agilent 400-MR spectrometer at 400 MHz (^{1}H) and 101 MHz (^{13}C) using Agilent VnmrJ Software V 4.2. Chemical shifts (δ), which are expressed in part per million (ppm), were determined relative to residual non-deuterated solvent as an internal reference: $CDCl_3$ (^{13}C NMR: δ = 77.16 ppm; ^{1}H NMR: δ = 7.26 ppm) and $(CD_3)_2SO$ (^{13}C NMR: δ = 39.52 ppm; ^{1}H NMR: δ = 2.50 ppm) or with an external reference (^{19}F: δ = 0 ppm for $CFCl_3$). Multiplicities of signals are described as follows: s = singlet, d = doublet, t = triplet, q = quartet, m = multiplet or overlap of non-equivalent resonances; br = broad signal. Coupling constants (*J*) are given in Hz. Each sample was analyzed once (n=1).

ESI-MS were recorded on a Varian 500-MS spectrometer (Agilent). ESI-HRMS were recorded on a MICROTOF spectrometer (Bruker) equipped with ESI ion source (Apollo) and direct injector with LC autosampler Agilent RR 1200. Each sample was measured once (n=1) and the obtained spectra were analyzed using Bruker Compass DataAnalysis 4.0 software.

Analytical LC–MS analysis was performed on an Agilent 1260 Infinity II LC/MS system controlled with Agilent OpenLAB CDS ChemStation Edition Rev. C.01.08 [216] and equipped with an Autosampler (G7129A), Binary Pump (G7112B), Diode Array Detector WR (G7115A), Fluorescence Detector Spectra, and Single Quadrupole MSD XT (G6135B). Each sample was analyzed once (n=1). Analysis was done by using a Ascentis® Express AQ-C18 UHPLC Column 2 µm, 5 cm x 2.1 mm with Mobile phase A: 25 mM $HCOONH_4$ (pH = 3.5) aqueous buffer/ Mobile Phase B: MeOH and the following gradient (Flow: 0.4 mL/min, 40°C):

Time (min)	%B
**0**	40
**2**	40
**8**	100
**9**	100
**10**	40
**15**	40

Preparative HPLC was performed on a combined Agilent1260/1290 Infinity II preparative system equipped with a 1290 Infinity II open-bed sampler (G7169B)/fraction collector (G7159B), 1260 Infinity II multiple wavelength detector (G7165A) and with:

Device A: 1260 Infinity II preparative binary pump (G7161A) and Agilent 5 Prep-C_{18}, 5 µm, 100 x 50 mm preparative column.

Device B: 1290 Infinity II preparative binary pump (G7161B) and Agilent Pursuit 10 C_{18}, 10 µm, 250 X 50 mm preparative column.

Compound *p*-7, *tert*-butyl (4-(mercaptomethyl)benzyl)carbamate :

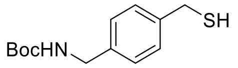

Chemical Formula: $C_{13}H_{19}NO_2S$
Molecular Weight: 253,36

***tert*-Butyl-4-(bromomethyl)benzylcarbamate (2.5 g, 8.32 mmol, 1.0 eq.)** and **thiourea (634 mg, 8.32 mmol, 1.0 eq.)** were refluxed together in **EtOH (21 mL, 0.4M)** for 2h.Water (1 mL) and **NaOH (664 mg, 16.6 mmol, 2.0 eq.)** were added. After 1h at 80°C, the mixture was diluted in water, the pH was lower to 2-3 using aqueous HCl 1M. The aqueous layer was extracted twice with AcOEt. Organic layers were combined, washed with brine and dried over Na_2SO_4. The crude was purified by flash chromatography on silica gel (120g, Hexanes/AcOEt from 90:10 to 60:40) to give the expected product as **a white solid (1.63 g, 6.44 mmol, η = 77 %**).

^{1}H NMR (400 MHz, $CDCl_3$): δ (ppm) = 7.28 (d, *J* = 8.0 Hz, 2H), 7.23 (d, *J* = 8.0 Hz, 2H), 4.81 (br s, 1H), 4.29 (br s, 2H), 3.72 (d, *J* = 7.5 Hz, 2H), 1.74 (t, *J* = 7.5 Hz, 1H), 1.46 (s, 9H).

^{13}C NMR (101 MHz, $CDCl_3$): δ (ppm) = 156.0 (C_q), 140.4 (C_q), 137.9 (C_q), 128.4 (CH), 127.9 (CH), 79.7 (C_q), 44.5 (CH_2), 28.8 (CH_2), 28.6 (CH_3).

ESI-MS, positive mode : m/z = 276.1 $[M+Na]^+$

ESI-MS, negative mode : m/z = 252.1 $[M-H]^-$

HRMS (ESI) calculated for $C_{13}H_{19}NO_2SNa^+$ $[M+Na]^+$: 276.1029, found: 276.1031.

Compound *m*-7, *tert*-butyl (3-(mercaptomethyl)benzyl)carbamate:

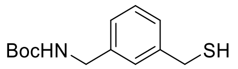

Chemical Formula: $C_{13}H_{19}NO_2S$
Molecular Weight: 253,36

***tert*-Butyl-3-(bromomethyl)benzylcarbamate (2.0 g, 6.66 mmol, 1.0 eq.)** and **thiourea (507 mg, 6.66 mmol, 1.0 eq.)** were refluxed together in **EtOH (17 mL, 0.4 M)** for 2h. **Water (1 mL)** and **NaOH (533 mg, 13.3 mmol, 2.0 eq.)** were added. After 1h at 80°C, the mixture was diluted in water, the pH was lower to 2-3 using aqueous HCl 1M. The aqueous layer was extracted twice with AcOEt. Organic layers were combined, washed with brine and dried over Na_2SO_4. The crude was purified by flash chromatography on silica gel (50 g, Hexanes/AcOEt from 100:0 to 80:20) to give the expected product as **white crystals (1450 mg, 5.72 mmol, η=86%).**

^{1}H NMR (400 MHz, $CDCl_3$): δ (ppm) = 7.35 – 7.17 (m, 3H), 7.17 – 7.13 (m, 1H), 4.90 (s, 1H), 4.27 (s, 2H), 3.70 (d, *J* = 7.5 Hz, 2H), 1.75 (t, *J* = 7.5 Hz, 1H), 1.45 (s, 9H).

^{13}C NMR (101 MHz, $CDCl_3$): δ (ppm) = 156.0 (C_q), 141.5 (C_q), 139.5 (C_q), 129.0 (CH), 127.1 (CH), 127.0 (CH), 126.2 (CH), 79.6 (C_q), 44.7 (CH_2), 28.9 (CH_2), 28.5 (CH_3).

ESI-MS, positive mode : m/z = 276.1 $[M+Na]^+$

ESI-MS, negative mode : m/z = 252.1 $[M-H]^-$

HRMS (ESI) calculated for $C_{13}H_{20}NO_2S^+$ $[M+H]^+$: 254.1209, found: 254.1213.

Compound *o*-7, *tert*-butyl (2-(mercaptomethyl)benzyl)carbamate:

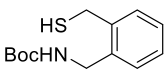

Chemical Formula: $C_{13}H_{19}NO_2S$
Molecular Weight: 253,36

***tert*-Butyl-2-(bromomethyl)benzylcarbamate (2.0 g, 6.66 mmol, 1.0 eq.)** and **thiourea (507 mg, 6.66 mmol, 1.0 eq.)** were refluxed together in **EtOH (17 mL, 0.4M)** for 2h. Water (1 mL) **and NaOH (533 mg, 13.3 mmol, 2.0 eq.)** were added. After 1h at 80°C, the mixture was diluted in water, the pH was lower to 2-3 using aqueous HCl 1M. The aqueous layer was extracted twice with AcOEt. Organic layers were combined, washed with brine and dried over Na_2SO_4. The crude was purified by flash chromatography on silica gel (80 g, Hexanes/AcOEt from 100:0 to 80:20) to give the expected product as **white crystals (1500 mg, 5.92 mmol, η=90%).**

^{1}H NMR (400 MHz, $CDCl_3$): δ (ppm) = 7.46 – 7.15 (m, 4H), 4.96 (s, 1H), 4.42 (s, 2H), 3.78 (d, *J* = 7.0 Hz, 2H), 1.79 (t, *J* = 7.0 Hz, 1H), 1.47 (s, 9H).

^{13}C NMR (101 MHz, $CDCl_3$): δ (ppm) = 155.8 (C_q), 139.2 (C_q), 136.3 (C_q), 129.5 (CH), 129.2 (CH), 128.1 (CH), 127.8 (CH), 79.7 (C_q), 42.1 (CH_2), 28.5 (CH_3), 26.1 (CH_2).

ESI-MS, positive mode : m/z = 276.1 $[M+Na]^+$

ESI-MS, negative mode : m/z = 252.1 $[M-H]^-$

HRMS (ESI) calculated for $C_{13}H_{20}NO_2S^+$ $[M+H]^+$: 254.1209, found: 254.1219.

Compound 8:

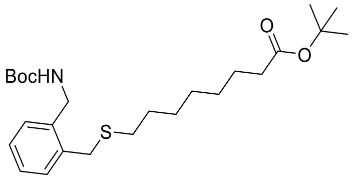

Chemical Formula: $C_{25}H_{41}NO_4S$
Molecular Weight: 451,66

Under Ar atmosphere, ***o*-7 (300 mg, 1.18 mmol, 1.0 eq.)** and **potassium carbonate (546 mg, 2.37 mmol, 2.0 eq.)** were mixed together in **dry DMF (12 mL, 0.1M).** ***Tert*-butyl 8-bromooctanoate (497 mg, 1.78 mmol, 1.5 eq.)** was added and the solution was stirred at rt. After 18h, water (25 mL) and AcOEt (30 mL) were added. The aqueous layer was extracted twice with AcOEt. Organic layers were combined, was washed with brine and dried over Na_2SO_4. The crude was purified by flash column chromatography on silica gel (10g, Hexanes/AcOEt from 100:0 to 85:15) to give the expected product as a colorless oil **(493 mg, 1.09 mmol, η = 92%)**.

^{1}H NMR (400 MHz, $CDCl_3$): δ (ppm) = 7.34 – 7.30 (m, 1H), 7.25 – 7.17 (m, 3H), 5.08 (s, 1H), 4.41 (s, 2H), 3.73 (s, 2H), 2.49 – 2.40 (m, 2H), 2.18 (t, *J* = 7.5 Hz, 2H), 1.63 – 1.51 (m, 4H), 1.44 (s, 9H), 1.43 (s, 9H), 1.36 – 1.20 (m, 6H).

^{13}C NMR (101 MHz, $CDCl_3$): δ (ppm) = 173.3 (C_q), 155.9 (C_q), 137.2 (C_q), 136.1 (C_q), 130.3 (CH), 129.3 (CH), 127.7 (CH), 127.5 (CH), 80.0 (C_q), 79.5 (C_q), 42.1 (CH_2), 35.6 (CH_2), 33.9 (CH_2), 29.3 (CH_2), 29.0 (CH_2), 29.0 (CH_2), 28.8 (CH_2), 28.5 (CH_3), 28.2 (CH_3), 25.1 (CH_2).

ESI-MS, positive mode : m/z = 452.2 $[M+H]^+$

HRMS (ESI) calculated for $C_{25}H_{42}NO_4S^+$ $[M+H]^+$: 452.2829, found: 452.2841.

Compound 9:

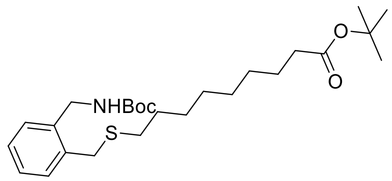

Chemical Formula: $C_{26}H_{43}NO_4S$
Molecular Weight: 465,69

Under Ar atmosphere, ***o*-7 (150 mg, 0.59 mmol, 1.0 eq.)** and **potassium carbonate (163 mg, 1.18 mmol, 2.0 eq.)** were mixed together in **dry DMF (6 mL, 0.1M). *Tert*-butyl 9-bromononanoate (249 mg, 0.89 mmol, 1.5 eq.)** was added and the solution was stirred at rt for 24h. AcOEt was added (20 mL). The organic layer was washed once with brine and dried over Na_2SO_4. The crude was purified by flash column chromatography on silica gel (10g, Hexanes/AcOEt from 100:0 to 85:15) to give the expected product as a colorless oil **(217 mg, 0.592 mmol, η = 79%)**.

^{1}H NMR (400 MHz, $CDCl_3$): δ (ppm) = 7.35 – 7.31 (m, 1H), 7.26 – 7.17 (m, 3H), 5.06 (s, 1H), 4.42 (s, 2H), 3.74 (s, 2H), 2.49 – 2.42 (m, 2H), 2.19 (t, J = 7.5 Hz, 2H), 1.62 – 1.53 (m, 4H), 1.45 (s, 9H), 1.44 (s, 9H), 1.38 – 1.23 (m, 8H).

^{13}C NMR (101 MHz, $CDCl_3$): δ (ppm) = 173.4 (C_q), 155.9 (C_q), 137.3 (C_q), 136.2 (C_q), 130.4 (CH), 129.4 (CH), 127.8 (CH), 127.5 (CH), 80.0 (C_q), 79.6 (C_q), 42.2 (CH_2), 35.7 (CH_2), 33.9 (CH_2), 32.1 (CH_2), 29.4 (CH_2), 29.3 (CH_2), 29.2 (CH_2), 29.1 (CH_2), 29.0, (CH_2) 28.6 (CH_3), 28.3 (CH_3), 25.2 (CH_2).

ESI-MS, positive mode : m/z = 466.2 $[M+H]^+$

HRMS (ESI) calculated for $C_{26}H_{44}NO_4S^+$ $[M+H]^+$: 466.2986, found: 466.2988.

Compound 10:

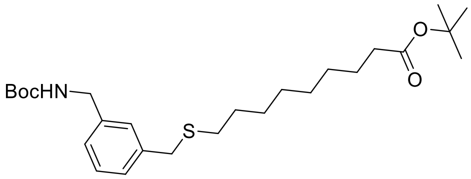

Chemical Formula: $C_{26}H_{43}NO_4S$
Molecular Weight: 465,69

Under Ar atmosphere, ***m*-7 (300 mg, 1.18 mmol, 1.0 eq.)** and **potassium carbonate (163 mg, 1.18 mmol, 2.0 eq.)** were mixed together in **dry DMF (6 mL, 0.1M). Tert-butyl 9-bromononanoate (249 mg, 0.79 mmol, 1.5 eq.)** was added and the solution was stirred at rt for 24h. AcOEt was added (20 mL). The organic layer was washed once with brine and dried over Na_2SO_4. The organic layer was washed once with brine and dried over Na_2SO_4. The crude was purified by flash column chromatography on silica gel (10g, Hexanes/AcOEt from 100:0 to 85:15) to give the expected product as a colorless oil **(256 mg, η = 93%)**.

^{1}H NMR (400 MHz, $CDCl_3$): δ (ppm) = 7.27 – 7.11 (m, 4H), 4.89 (s, 1H), 4.27 (s, 2H), 3.66 (s, 2H), 2.42 – 2.35 (m, 2H), 2.17 (t, J = 7.5 Hz, 2H), 1.60 – 1.49 (m, 4H), 1.44 (s, 9H), 1.42 (s, 9H), 1.38 – 1.21 (m, 8H).

^{13}C NMR (101 MHz, $CDCl_3$): δ (ppm) = 173.3 (C_q), 156.0 (C_q), 139.3 (C_q), 139.1 (C_q), 128.8 (CH), 127.9 (CH), 127.9 (CH), 126.1 (CH), 80.0 (C_q), 79.6 (C_q), 44.7 (CH_2), 36.3 (CH_2), 35.7 (CH_2), 31.6 (CH_2), 29.2 (CH_2), 29.2 (CH_2), 29.1 (CH_2), 29.1 (CH_2), 28.9 (CH_2), 28.5 (CH_3), 28.2 (CH_3), 25.1 (CH_2).

ESI-MS, positive mode : m/z = 466.3 $[M+H]^+$

HRMS (ESI) calculated for $C_{26}H_{44}NO_4S^+$ $[M+H]^+$: 466.2986, found: 466.2992.

Compound 11:

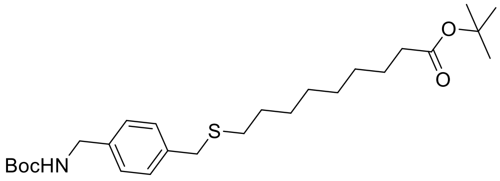

Chemical Formula: $C_{26}H_{43}NO_4S$
Molecular Weight: 465,69

Under Ar atmosphere, ***p*-7 (150 mg, 0.59 mmol, 1.0 eq.)** and **potassium carbonate (163 mg, 1.18 mmol, 2.0 eq.)** were mixed together in **dry DMF (6 mL, 0.1M). Tert-butyl 9-bromononanoate (249 mg, 0.89 mmol, 1.5 eq.)** was added and the solution was stirred at rt for 24h. AcOEt was added (20 mL). The organic layer was washed once with brine and dried over Na_2SO_4. The crude was purified by flash column chromatography on silica gel (10g, Hexanes/AcOEt from 100:0 to 80:20) to give the expected product as a colorless oil **(227 mg, 0.59 mmol, η = 82%)**.

^{1}H NMR (400 MHz, $CDCl_3$): δ (ppm) = 7.22 (d, *J* = 8.0 Hz, 2H), 7.17 (d, *J* = 8.0 Hz, 2H), 4.95 (s, 1H), 4.24 (s, 2H), 3.63 (s, 2H), 2.38 – 2.31 (m, 2H), 2.15 (t, *J* = 7.5 Hz, 2H), 1.56 – 1.47 (m, 4H), 1.42 (s, 9H), 1.41 (s, 9H), 1.32 – 1.20 (m, 8H).

^{13}C NMR (101 MHz, $CDCl_3$): δ (ppm) = 173.2 (C_q), 155.9 (C_q), 137.7 (C_q), 137.6 (C_q), 129.0 (CH), 127.5 (CH), 79.9 (C_q), 79.4 (C_q), 44.4 (CH_2), 35.9 (CH_2), 35.6 (CH_2), 31.3 (CH_2), 29.1 (CH_2), 29.1 (CH_2), 29.0 (CH_2), 29.0 (CH_2), 28.8 (CH_2), 28.4 (CH_3), 28.1 (CH_3), 25.0 (CH_2).

ESI-MS, positive mode : m/z = 466.2 $[M+H]^+$

HRMS (ESI) calculated for $C_{26}H_{43}NO_4SNa^+$ $[M+Na]^+$: 488.2805, found: 488.2804.

Compound 12:

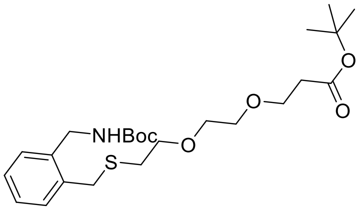

Chemical Formula: $C_{24}H_{39}NO_6S$
Molecular Weight: 469,63

Under Ar atmosphere, ***o*-7 (100 mg, 0.39 mmol, 1.0 eq.)** and **potassium carbonate (108 mg, 0.78 mmol, 2.0 eq.)** were mixed together in **dry DMF (4 mL, 0.1M). Bromo-PEG$_2$- *tert*-butyl ester (129 mg, 0.43 mmol, 1.1 eq.)** was added and the solution was stirred at rt for 18h. AcOEt (20 mL) was added. The organic layer was washed twice with brine and dried over Na_2SO_4. The crude was purified by flash column chromatography on silica gel (10g, Hexanes/AcOEt from 70:30 to 0:100) to give the expected product as a colorless oil **(140 mg, 0.30 mmol, η = 76 %)**.

^{1}H NMR (400 MHz, $CDCl_3$): δ (ppm) = 7.36 – 7.32 (m, 1H), 7.27 – 7.17 (m, 3H), 4.43 (d, *J* = 3.5 Hz, 2H), 3.84 (s, 2H), 3.71 (t, *J* = 6.5 Hz, 2H), 3.65 – 3.56 (m, 6H), 2.66 (t, *J* = 6.5 Hz, 2H), 2.50 (t, *J* = 6.5 Hz, 2H), 1.46 (s, 9H), 1.44 (s, 9H).

^{13}C NMR (101 MHz, $CDCl_3$): δ (ppm) = 171.0 (C_q), 155.9 (C_q), 137.4 (C_q), 136.0 (C_q), 130.5 (CH), 129.5 (CH), 127.9 (CH), 127.6 (CH), 80.7 (C_q), 79.7 (C_q, from HMBC), 71.2 (CH_2), 70.5 (CH_2), 70.4 (CH_2), 67.0 (CH_2), 42.1 (CH_2), 36.4 (CH_2), 34.3 (CH_2), 31.3 (CH_2), 28.6 (CH_3), 28.2 (CH_3).

ESI-MS, positive mode : m/z = 470.2 $[M+H]^+$

HRMS (ESI) calculated for $C_{24}H_{39}NO_6SNa^+$ $[M+Na]^+$: 492.2390, found: 492.2392.

Compound 13:

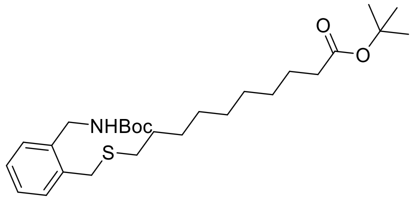

Chemical Formula: $C_{27}H_{45}NO_4S$
Molecular Weight: 479,72

Under Ar atmosphere, ***o*-7 (100 mg, 0.40 mmol, 1.0 eq.)** and **potassium carbonate (109 mg, 0.79 mmol, 2.0 eq.)** were mixed together in **dry DMF (4 mL, 0.1M). Tert-butyl 10-bromodecanoate (147 mg, 0.47 mmol, 1.2 eq.)** was added and the solution was stirred at rt for 24h. AcOEt (20 mL) was added. The organic layer was washed once with brine and dried over Na_2SO_4. The crude was purified by flash column chromatography on silica gel (10g, Hexanes/AcOEt from 100:0 to 80:20) to give the expected product as a colorless oil **(133 mg, 0.27 mmol, η = 70%)**.

^{1}H NMR (400 MHz, $CDCl_3$): δ (ppm) = 7.34 – 7.31 (m, 1H), 7.25 – 7.16 (m, 3H), 5.05 (s, 1H), 4.42 (s, 2H), 3.74 (s, 2H), 2.48 – 2.42 (m, 2H), 2.21 – 2.15 (m, 2H), 1.61 – 1.51 (m, 4H), 1.45 (s, 9H), 1.43 (s, 9H), 1.36 – 1.23 (m, 10H).

^{13}C NMR (101 MHz, $CDCl_3$): δ (ppm) = 173.4 (C_q), 155.9 (C_q), 137.2 (C_q), 136.2 (C_q), 130.4 (CH), 129.4 (CH), 127.8 (CH), 127.5 (CH), 80.0 (C_q), 79.6 (C_q), 42.2 (CH_2), 35.7 (CH_2), 33.9 (CH_2), 32.1 (CH_2), 29.4 (CH_2), 29.3 (CH_2), 29.3 (CH_2), 29.2 (CH_2), 29.0 (CH_2), 28.6 (CH_3), 28.2 (CH_3), 25.2 (CH_2).

ESI-MS, positive mode : m/z = 480.3 $[M+H]^+$

HRMS (ESI) calculated for $C_{27}H_{46}NO_4S^+$ $[M+H]^+$: 480.3142, found: 480.3142.

General procedure for sulfonium synthesis:

The thioether (1.0 eq) was solubilized in **a HCOOH/AcOH mixture (1:1, 0.5M)**. **Methyl trifluoromethanesulfonate (3.0 eq.)** was added and the solution was stirred at rt for 1h. The mixture was diluted in acetonitrile and purified by reverse-phase HPLC (Device A, H_2O+0.1%TFA/ACN, linear gradient from 95:5 to 0:100, 40 mL/min). Solvents were removed under vacuum. As this type of compounds is highly hygroscopic, the final product was dissolved in a precise amount of **DMSO-d$_6$.** Concentration of the samples and therefore yields were determined by ^{1}H-NMR with an external standard. The products were kept in DMSO-d$_6$ (stored with preactivated powdered 4Å molecular sieves) and use in the final step without any further treatments.

Compound 14:

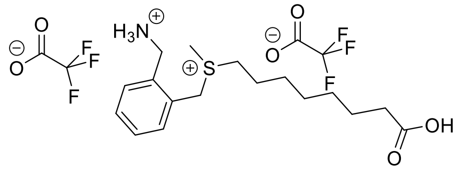

Chemical Formula: $C_{21}H_{29}F_6NO_6S$
Molecular Weight: 537,51

The general procedure starting with **8 (70 mg, 0.155 mmol, 1.0 eq.)** gave the expected product as **a colorless oil (0.140 mmol, η = 90 %).**

^{1}H NMR (400 MHz, DMSO-d$_6$): δ(ppm) = 8.53 (s, 3H), 7.63 (dd, *J* = 7.5, 1.5 Hz, 1H), 7.58 – 7.44 (m, 3H), 4.92 (d, *J* = 13.0 Hz, 1H), 4.83 (d, *J* = 13.0 Hz, 1H), 4.27 – 4.23 (m, 2H), 3.40 – 3.29 (m, 2H), 2.87 (s, 3H), 2.18 (t, *J* = 7.5 Hz, 2H), 1.74 – 1.63 (m, 1H), 1.60 – 1.44 (m, 3H), 1.36 – 1.20 (m, 6H).

^{13}C NMR (101 MHz, DMSO-d$_6$): δ(ppm) = 174.5 (C_q), 158.8 (q, J_F = 34.2 Hz), 134.4 (C_q), 132.3 (CH), 131.0 (CH), 130.2 (CH), 129.5 (CH), 127.8 (C_q), 116.4 (q, J_F = 294.5 Hz), 42.8 (CH_2), 40.9 (CH_2), 38.9 (CH_2), 33.7 (CH_2), 28.2 (CH_2), 28.0 (CH_2), 27.7 (CH_2), 24.4 (CH_2), 23.4 (CH_2), 22.0 (CH_3).

^{19}F NMR (376 MHz, DMSO-d$_6$): δ(ppm) = -74.5.

ESI-MS, positive mode: m/z = 310.1 [M-H-2CF_3CO_2]$^+$

ESI-MS, negative mode: m/z = 308.1 [M-2H-2CF_3CO_2]$^-$

HRMS (ESI) calculated for $C_{17}H_{28}NO_2S^+$ [M-H-2CF_3CO_2]$^+$: 310.1835, found: 310.1836.

Compound 15:

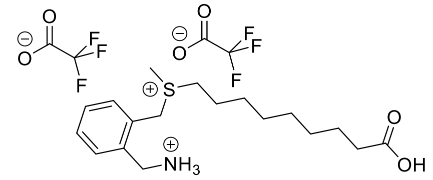

Chemical Formula: $C_{22}H_{31}F_6NO_6S$
Molecular Weight: 551,54

The general procedure starting with **9 (100 mg, 0.215 mmol, 1.0 eq.)** gave the expected product as **a colorless oil (0.210 mmol, η = 98 %).**

^{1}H NMR (400 MHz, DMSO-d$_6$): δ(ppm) = 8.55 (s, 3H, NH), 7.62 (dd, *J* = 7.5, 1.5 Hz, 1H), 7.57 – 7.44 (m, 3H), 4.92 (d, *J* = 13.0 Hz, 1H), 4.83 (d, *J* = 13.0 Hz, 1H), 4.24 (t, *J* = 5.5 Hz, 2H), 3.38 – 3.29 (m, 2H), 2.86 (s, 3H), 2.18 (t, *J* = 7.5 Hz, 2H), 1.76 – 1.60 (m, 1H), 1.59 - 1.43 (m, 3H), 1.34 – 1.19 (m, 8H).

^{13}C NMR (101 MHz, DMSO-d$_6$): δ(ppm) = 174.6 (C_q), 158.8 (q, J_F = 34.3 Hz), 134.5 (C_q), 132.3 (CH), 131.0 (CH), 130.2 (CH), 129.5 (CH), 127.8 (C_q), 116.5 (q, J_F = 294.7 Hz), 42.9 (CH_2), 40.9 (CH_2), 38.9 (CH_2), 33.8 (CH_2), 28.5 (CH_2), 28.4 (CH_2), 28.2 (CH_2), 27.8 (CH_2), 24.5 (CH_2), 23.4 (CH_2), 22.0 (CH_3).

^{19}F NMR (376 MHz, DMSO-d$_6$): δ(ppm) = -74.5.

ESI-MS, positive mode: m/z = 324.2 [M-H-2CF_3CO_2]$^+$

ESI-MS, negative mode: m/z = 322.2 [M-2H-2CF_3CO_2]$^-$

HRMS (ESI) calculated for $C_{18}H_{30}NO_2S^+$ [M-H-2CF_3CO_2]$^+$: 324.1992, found: 324.1993.

Compound 16:

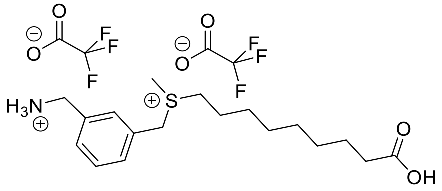

Chemical Formula: $C_{22}H_{31}F_6NO_6S$
Molecular Weight: 551,54

The general procedure starting with **10 (100 mg, 0.215 mmol, 1.0 eq.)** gave the expected product as **a colorless oil (0.210 mmol, η = 98 %).**

^{1}H NMR (400 MHz, DMSO-d$_6$): δ(ppm) = 8.49 (s, 3H), 7.61 – 7.48 (m, 4H), 4.78 (d, *J* = 13.0 Hz, 1H), 4.69 (d, *J* = 13.0 Hz, 1H), 4.08 (q, *J* = 5.5 Hz, 2H), 3.33 – 3.20 (m, 2H), 2.81 (s, 3H), 2.19 (t, *J* = 7.5 Hz, 2H), 1.78 – 1.59 (m, 2H), 1.55 – 1.42 (m, 2H), 1.40 – 1.20 (m, 8H).

^{13}C NMR (101 MHz, DMSO-d$_6$): δ(ppm) = 174.6 (C$_q$), 158.8 (q, J_F = 35.1 Hz), 135.4 (C$_q$), 131.2 (CH), 130.9 (CH), 130.3 (CH), 129.7 (CH), 128.8 (C$_q$), 116.2 (q, J_F = 293.1 Hz), 44.4 (CH$_2$), 42.1 (CH$_2$), 40.6 (CH$_2$), 33.8 (CH$_2$), 28.5 (CH$_2$), 28.5 (CH$_2$), 28.2 (CH$_2$), 27.8 (CH$_2$), 24.6 (CH$_2$), 23.4 (CH$_2$), 21.6 (CH$_3$).

^{19}F NMR (376 MHz, DMSO-d$_6$): δ(ppm) = -74.7.

ESI-MS, positive mode : m/z = 324.2 [M-H-2CF$_3$CO$_2$]$^+$

ESI-MS, negative mode : m/z = 322.2 [M-2H-2CF$_3$CO$_2$]$^-$

HRMS (ESI) calculated for $C_{18}H_{30}NO_2S^+$ [M-H-2CF$_3$CO$_2$]$^+$: 324.1992, found: 324.1992.

Compound 17:

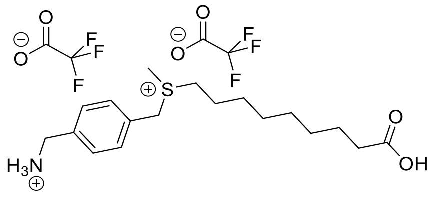

Chemical Formula: $C_{22}H_{31}F_6NO_6S$
Molecular Weight: 551,54

The general procedure starting with **11 (100 mg, 0.215 mmol, 1.0 eq.)** gave the expected product as **a colorless oil (0.196 mmol, η = 91 %).**

^{1}H NMR (400 MHz, DMSO-d$_6$): δ(ppm) = 8.49 (s, 3H), 7.62 – 7.48 (m, 4H), 4.78 (d, J = 13.0 Hz, 1H), 4.71 (d, J = 13.0 Hz, 1H), 4.08 (q, J = 5.5 Hz, 2H), 3.32 – 3.17 (m, 2H), 2.80 (s, 3H), 2.19 (t, J = 7.5 Hz, 2H), 1.78 – 1.59 (m, 2H), 1.52 – 1.45 (m, 2H), 1.38 – 1.17 (m, 8H).

^{13}C NMR (101 MHz, DMSO-d$_6$): δ(ppm) = 174.6 (C$_q$), 158.8 (q, J_F = 34.7 Hz), 135.7 (C$_q$), 130.9 (CH), 129.8 (CH), 128.7 (C$_q$), 116.3 (q, J_F = 293.8 Hz), 44.3 (CH$_2$), 42.0 (CH$_2$), 40.6 (CH$_2$), 33.8 (CH$_2$), 28.5 (CH$_2$), 28.5 (CH$_2$), 28.2 (CH$_2$), 27.8 (CH$_2$), 24.6 (CH$_2$), 23.3 (CH$_2$), 21.7 (CH$_3$).

^{19}F NMR (376 MHz, DMSO-d$_6$): δ(ppm) = -74,7.

ESI-MS, positive mode: m/z = 324.1 [M-H-2CF$_3$CO$_2$]$^+$

ESI-MS, negative mode: m/z = 322.1 [M-2H-2CF$_3$CO$_2$]$^-$

HRMS (ESI) calculated for $C_{18}H_{30}NO_2S^+$ [M-H-2CF$_3$CO$_2$]$^+$: 324.1992, found: 324.1999.

Compound 18:

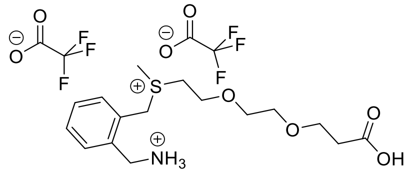

Chemical Formula: $C_{20}H_{27}F_6NO_8S$
Molecular Weight: 555,49

The general procedure starting with **12 (75 mg, 0.160 mmol, 1.0 eq.)** gave the expected product as **a colorless oil (0.088 mmol, η = 55 %).**

^{1}H NMR (400 MHz, DMSO-d$_6$): δ(ppm) = 8.47 (s, 3H), 7.65 – 7.60 (m, 1H), 7.59 – 7.44 (m, 3H), 4.93 (d, J = 13.0 Hz, 1H), 4.83 (d, J = 13.0 Hz, 1H), 4.23 (q, J = 5.5 Hz, 2H), 3.91 – 3.78 (m, 2H), 3.71 – 3.43 (m, 8H), 2.88 (s, 3H), 2.43 (t, J = 6.0 Hz, 2H).

^{13}C NMR (101 MHz, DMSO-d$_6$): δ(ppm) = 172.7 (C$_q$), 158.6 (q, J_F = 35.0 Hz, C$_q$), 134.5 (C$_q$), 132.4 (CH), 130.8 (CH), 130.3 (CH), 129.5 (CH), 127.6 (C$_q$), 116.1 (q, J_F = 294.9 Hz, C$_q$), 69.7 (CH$_2$), 69.3 (CH$_2$), 66.3 (CH$_2$), 64.4 (CH$_2$), 43.1 (CH$_2$), 41.6 (CH$_2$), 38.8 (CH$_2$), 34.8 (CH$_2$), 22.5 (CH$_3$).

^{19}F NMR (376 MHz, DMSO-d$_6$): δ(ppm) = -74.6.

ESI-MS, positive mode: m/z = 328.1 [M-H-2CF$_3$CO$_2$]$^+$

ESI-MS, negative mode: m/z = 326.1 [M-2H-2CF$_3$CO$_2$]$^-$

HRMS (ESI) calculated for $C_{16}H_{26}NO_4S^+$ [M-H-2CF$_3$CO$_2$]$^+$: 325.1577, found: 325.1582.

Compound 19:

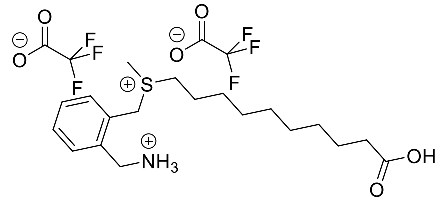

Chemical Formula: $C_{23}H_{33}F_6NO_6S$
Molecular Weight: 565,56

The general procedure starting with **13 (40 mg, 0.083 mmol, 1.0 eq.)** gave the expected product as **a colorless oil (0.080 mmol, η = 96 %).**

^{1}H NMR (400 MHz, DMSO-d$_6$): δ(ppm) = 8.52 (s, 3H), 7.66 – 7.59 (m, 1H), 7.59 – 7.42 (m, 3H), 4.90 (d, J = 13.0 Hz, 1H), 4.82 (d, J = 13.0 Hz, 1H), 4.24 (s, 2H), 3.39 – 3.28 (m, 2H), 2.86 (s, 3H), 2.18 (t, J = 7.5 Hz, 2H), 1.73 – 1.63 (m, 1H), 1.60 – 1.42 (m, 3H), 1.36 – 1.28 (m, 2H), 1.27 – 1.20 (m, 8H).

^{13}C NMR (101 MHz, DMSO-d$_6$): δ(ppm) = 174.5 (C$_q$), 158.6 (q, J_F = 34.9 Hz), 134.3 (C$_q$), 132.3 (CH), 130.9 (CH), 130.2 (CH), 129.4 (CH), 127.7 (C$_q$), 116.2 (q, J_F = 293.6 Hz), 42.7 (CH$_2$), 40.8 (CH$_2$), 38.8 (CH$_2$), 33.7 (CH$_2$), 28.6 (CH$_2$), 28.5 (CH$_2$), 28.5 (CH$_2$), 28.2 (CH$_2$), 27.8 (CH$_2$), 24.5 (CH$_2$), 23.3 (CH$_2$), 21.9 (CH$_3$).

^{19}F NMR (376 MHz, DMSO-d$_6$): δ(ppm) = -74.6.

ESI-MS, positive mode: m/z = 338.2 [M-H-2CF$_3$CO$_2$]$^+$

ESI-MS, negative mode: m/z = 336.2 [M-2H-2CF$_3$CO$_2$]$^-$

HRMS (ESI) calculated for $C_{19}H_{32}NO_2S^+$ [M-H-2CF$_3$CO$_2$]$^+$: 338.2148, found: 338.2147.

CTX-NH$_2$.CF$_3$OCO$_2$H:

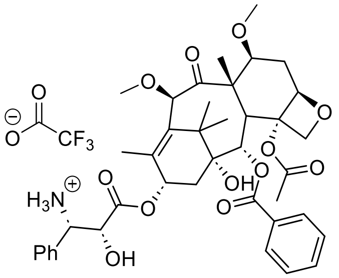

Chemical Formula: $C_{42}H_{50}F_3NO_{14}$
Molecular Weight: 849,85

A solution of **cabazitaxel (1 g, 1.20 mmol, 1.0 eq.)** in **95% formic acid (5 mL)** was stirred at room temperature for 4h. Reaction progress was monitored by analytical HPLC. Once reaction was complete, formic acid was evaporated on rotary evaporator. The residue was solubilized in a H_2O/ACN mixture and purified by reverse-phase HPLC (Device B,

H_2O+0.1%TFA/ACN, linear gradient 70:30 to 0:100, 150 mL/min). The fractions containing the product were lyophilized to obtain the expected compound as a white powder **(660 mg, 0.78 mmol, η = 65 %)**.

^{1}H NMR (400 MHz, DMSO-d$_6$): δ(ppm) = 8.71 (s, 3H, NH_3), 7.94 – 7.89 (m, 2H), 7.76 – 7.71 (m, 1H), 7.67 – 7.62 (m, 2H), 7.53 – 7.42 (m, 4H), 7.35 – 7.30 (m, 1H), 5.84 (td, *J* = 9.0, 2.0 Hz, 1H), 5.34 (d, *J* = 7.0 Hz, 1H), 4.93 (dd, *J* = 9.5, 2.0 Hz, 1H), 4.67 (s, 1H), 4.49 – 4.38 (m, 2H), 4.01 – 3.96 (m, 2H), 3.72 (dd, *J* = 10.5, 6.5 Hz, 1H), 3.55 (d, *J* = 7.0 Hz, 1H), 3.29 (s, 3H), 3.20 (s, 3H), 2.67 – 2.60 (m, 1H), 2.08 (s, 3H), 1.80 (s, 3H), 1.68 (dd, *J* = 15.5, 9.0 Hz, 1H), 1.57 – 1.42 (m, 5H), 0.97 (s, 3H), 0.94 (s, 3H).

^{13}C NMR (101 MHz, DMSO-d$_6$): δ(ppm) = 204.7 (C_q), 171.2 (C_q), 169.8 (C_q), 165.1 (C_q), 158.3 (q, J_F = 32.0 Hz), 137.9 (C_q), 135.1 (C_q), 133.5 (CH), 133.4 (C_q), 129.9 (C_q), 129.5 (CH), 129.3 (CH), 128.9 (CH), 128.7 (CH), 128.0 (CH), 117.0 (q, J_F = 296.5 Hz), 83.2 (CH), 82.0 (CH), 80.3 (C_q), 80.2 (CH), 76.8 (C_q), 75.2 (CH_2), 74.3 (CH), 73.0 (CH), 70.2 (CH), 57.0 (CH), 56.7 (CH_3), 56.7 (CH_3), 56.0 (C_q), 46.4 (CH), 42.9 (C_q), 34.7 (CH_2), 31.7 (CH_2), 26.7 (CH_3), 22.5 (CH_3), 21.2 (CH_3), 14.0 (CH_3), 10.2 (CH_3).

^{19}F NMR (376 MHz, DMSO-d$_6$): δ(ppm) = -73.9.

ESI-MS, positive mode: m/z = 736.2 [M-$CF_3OCO_2^-$]

ESI-MS, negative mode: m/z = 780.30 [M-CF_3OCO_2H + $HCOO^-$]

HRMS (ESI) calculated for $C_{40}H_{50}NO_{12}^+$ [M-CF_3CO_2]$^+$: 736.3328, found: 736.3347.

Characterization of the final probes

Probe 1 :

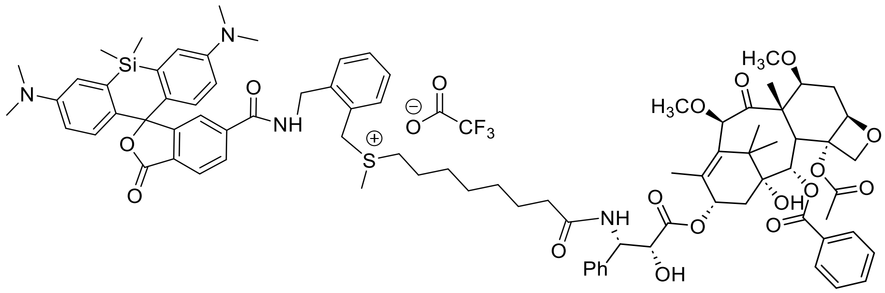

Exact Mass: 1481,67

According to the general procedure, **6-SiR-CO$_2$H** (2.0 mg, 4.23 µmol), sulfonium **14** (13.8 µL -of a 0.4M solution in DMSO- ,5.50 µmol) and **CTX-NH$_2$.CF$_3$CO$_2$H** (10.8 mg, 12.7 µmol) led to the expected product as a blue powder (2.46 µmol, **η = 58 %**).

^{1}H NMR (400 MHz, DMSO-*d$_6$*): δ = 9.33 (t, *J* = 5.5 Hz, 1H, NH), 8.36 (d, *J* = 9.0 Hz, 1H, NH), 8.14 (dd, *J* = 8.0, 1.5 Hz, 1H, H_{Ar}), 8.07 (d, *J* = 8.0 Hz, 1H, H_{Ar}), 8.00 – 7.96 (m, 2H, H_{Ar}), 7.72 – 7.66 (m, 2H, H_{Ar}), 7.62 – 7.57 (m, 2H, H_{Ar}), 7.46 – 7.31 (m, 8H, H_{Ar}), 7.24 – 7.20 (m, 1H, H_{Ar}), 7.04 (s, 2H, H_{Ar}), 6.67 -6.62 (m, 4H, H_{Ar}), 5.96 – 5.91 (m, 1H, CH), 5.39 (d, *J* = 7.0 Hz, 1H, CH), 5.32 – 5.27 (m, 1H, CH), 4.98 – 4.94 (m, 1H, CH), 4.86 (d, *J* = 13.0 Hz, 1H, 1H from CH_2), 4.76 (d, *J* = 13.0 Hz, 1H, 1H from CH_2), 4.71 (s, 1H, CH), 4.56 (d, *J* = 5.5 Hz, 2H, CH_2), 4.42 (d, *J* = 5.5 Hz, 1H, CH), 4.03 (s, 2H, CH_2), 3.76 – 3.73 (dd, *J* = 10.5, 6.5 Hz, 1H, CH), 3.63 (d, *J* = 7.0 Hz, 1H, CH), 3.33 – 3.26 (m, 5H, CH_3 + CH_2), 3.21 (s, 3H, CH_3), 2.93 (s, 12H, 4*CH_3), 2.84 (s, 3H, CH_3), 2.68 – 2.63 (m, 1H, 1H from CH_2), 2.25 (s, 3H, CH_3), 2.19 – 2.11 (td, *J* = 7.0, 4.0 Hz, 2H, CH_2), 2.00 – 1.94 (m, 1H, 1H from CH_2), 1.90 – 1.81 (m, 4H, CH_3 +1H from CH_2), 1.60 – 1.41 (m, 8H, 1*CH_3 + 2*CH_2 + 1H from CH_2), 1.24 – 1.13 (m, 6H, 3*CH_2), 1.02 (s, 3H, CH_3), 0.98 (s, 3H, CH_3), 0.62 (s, 3H, CH_3), 0.52 (s, 3H, CH_3).

^{19}F NMR (376 MHz, DMSO-d$_6$): δ(ppm) = -74,6.

ESI-MS, positive mode: m/z = 1481.5 [M-$CF_3CO_2^-$]$^+$

HRMS (ESI) calculated for $C_{84}H_{101}N_4O_{16}SSi^+$ [M-$CF_3CO_2^-$]$^+$: 1481.6697, found: 1481.6697.

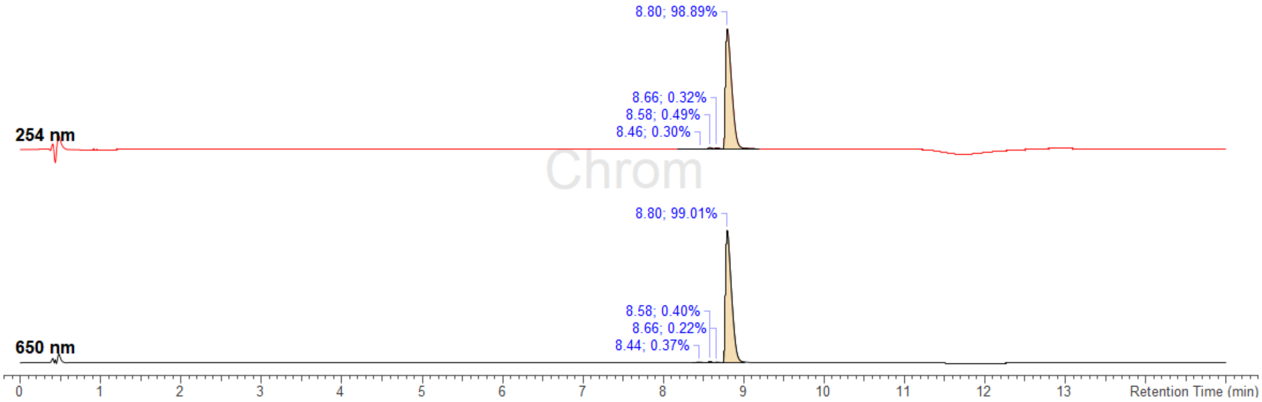

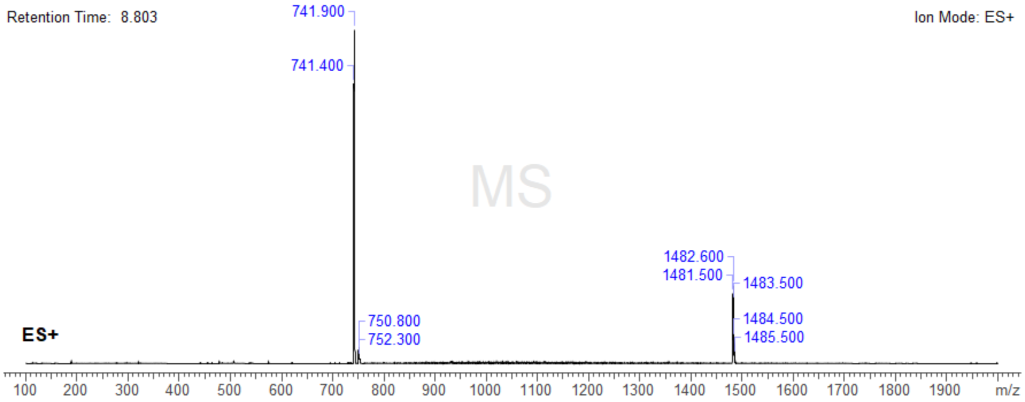

Analysis Info		Acquisition Date	04.09.2024 23:01:49	
Analysis Name	Z:\Data\2024\2409\sam040924\MA230_30_01_127436.d			
Method	hystar_p.m	Operator	BDAL@DE	
Sample Name	MA230	Instrument / Ser#	micrOTOF	10237
Comment				

Acquisition Parameter					
Source Type	ESI	Ion Polarity	Positive	Set Nebulizer	0.4 Bar
Focus	Not active			Set Dry Heater	180 °C
Scan Begin	50 m/z	Set Capillary	4500 V	Set Dry Gas	4.0 l/min
Scan End	3000 m/z	Set End Plate Offset	-500 V	Set Divert Valve	Source

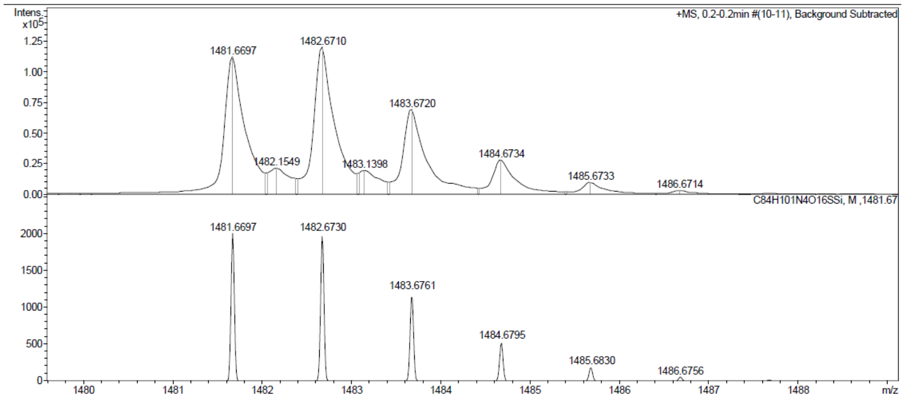

Probe 2 - 6-SiR-*o*-C9-CTX:

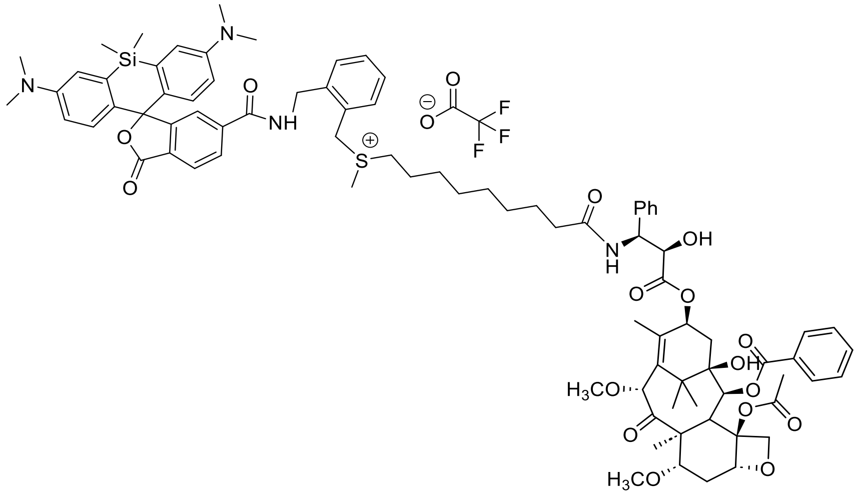

Exact Mass: 1495,69

According to the general procedure, **6-SiR-CO_2H** (3.0 mg, 6.35 µmol), sulfonium **15** (27.5 µL -of a 0.3M solution in DMSO-, 8.26 µmol) and **CTX-NH_2.CF_3CO_2H** (16.2 mg, 19.0 µmol) led to the expected product as a blue powder (3.24 µmol, **η = 51 %**).

^{1}H NMR (400 MHz, DMSO-*d*$_6$): δ = 9.34 (t, *J* = 5.5 Hz, 1H, NH), 8.36 (d, *J* = 9.0 Hz, 1H, NH), 8.14 (dd, *J* = 8.0, 1.5 Hz, 1H, H_{Ar}), 8.07 (d, *J* = 8.0 Hz, 1H, H_{Ar}), 8.00 – 7.96 (m, 2H, H_{Ar}), 7.72 – 7.66 (m, 2H, H_{Ar}), 7.62 – 7.57 (m, 2H, H_{Ar}), 7.48 – 7.28 (m, 8H, H_{Ar}), 7.24 – 7.20 (m, 1H, H_{Ar}), 7.04 (s, 2H, H_{Ar}), 6.66 -6.64 (m, 4H, H_{Ar}), 5.96 – 5.91 (m, 1H, CH), 5.40 (d, *J* = 7.0 Hz, 1H, CH), 5.30 (dd, *J* = 9.0, 5.5 Hz, 1H, CH), 4.96 (dd, *J* = 10.0, 2.0 Hz, 1H, CH), 4.86 (d, *J* = 13.0 Hz, 1H, 1H from CH_2), 4.76 (d, *J* = 13.0 Hz, 1H, 1H from CH_2), 4.71 (s, 1H, CH), 4.56 (d, *J* = 5.5 Hz, 2H, CH_2), 4.43 (d, *J* = 5.5 Hz, 1H, CH), 4.03 (s, 2H, CH_2), 3.76 – 3.73 (m, 1H, CH, from HSQC), 3.63 (d, *J* = 7.0 Hz, 1H, CH), 3.33 – 3.27 (m, 5H, CH_3 + CH_2), 3.21 (s, 3H, CH_3), 2.93 (s, 12H, 4*CH_3), 2.84 (s, 3H, CH_3), 2.71 – 2.61 (m, 1H, 1H from CH_2), 2.25 (s, 3H, CH_3), 2.19 – 2.11 (m, 2H, CH_2), 2.01 – 1.94 (m, 1H, 1H from CH_2), 1.91 – 1.81 (m, 4H, CH_3 +1H from CH_2), 1.64 – 1.42 (m, 8H, 1*CH_3 + 2*CH_2 + 1H from CH_2), 1.24 – 1.13 (m, 8H, 4*CH_2), 1.03 (s, 3H, CH_3), 0.98 (s, 3H, CH_3), 0.62 (s, 3H, CH_3), 0.52 (s, 3H, CH_3).

^{19}F NMR (376 MHz, DMSO-d$_6$): δ(ppm) = -74,6.

ESI-MS, positive mode: m/z = 1495.5 [M-$CF_3CO_2^-$]$^+$

HRMS (ESI) calculated for $C_{85}H_{103}N_4O_{16}SSi^+$ [M-$CF_3CO_2^-$]$^+$: 1495.6854, found: 1495.6874.

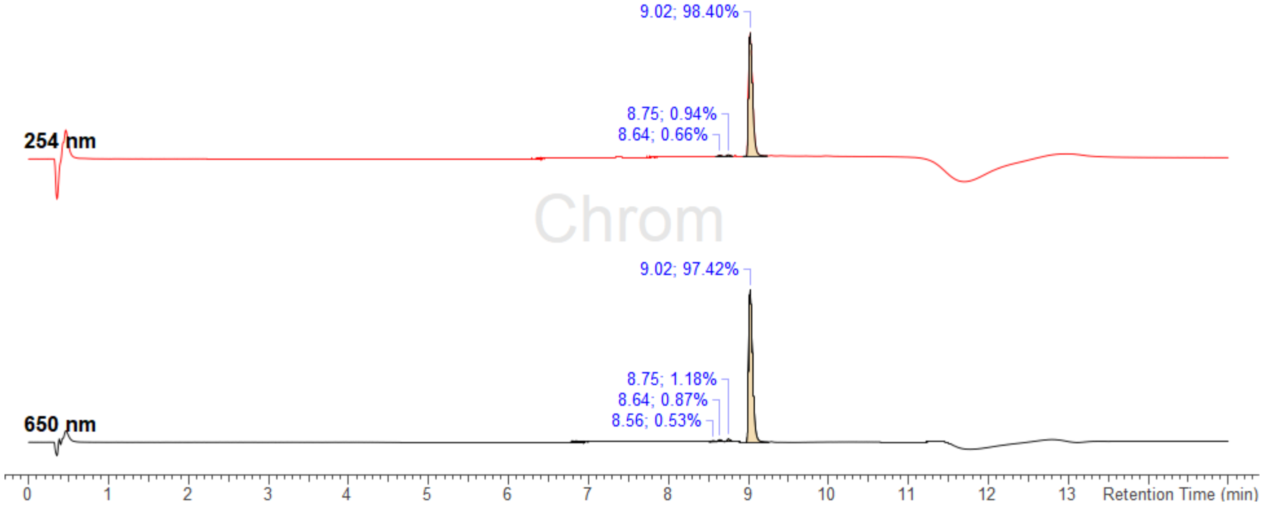

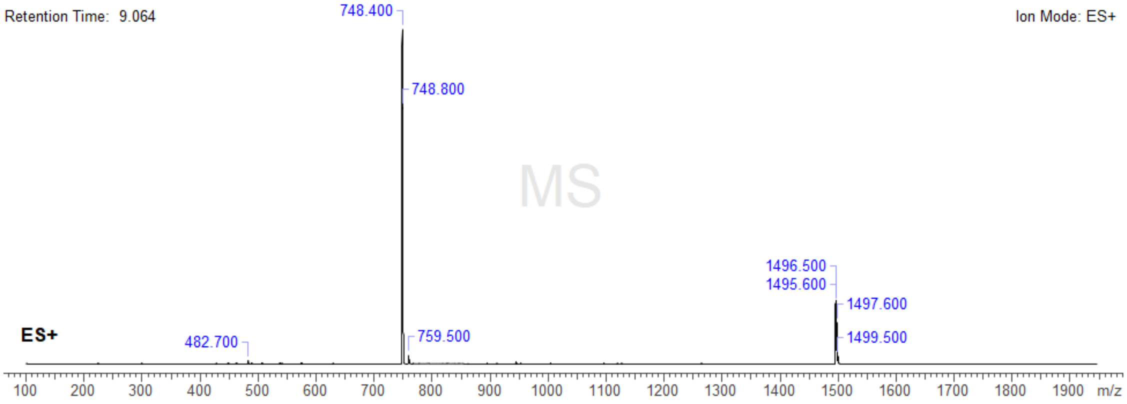

Analysis Info		Acquisition Date	04.09.2024 22:43:15	
Analysis Name	Z:\Data\2024\2409\sam040924\MA280_26_01_127432.d			
Method	hystar_p.m	Operator	BDAL@DE	
Sample Name	MA280	Instrument / Ser#	micrOTOF	10237
Comment				

Acquisition Parameter					
Source Type	ESI	Ion Polarity	Positive	Set Nebulizer	0.4 Bar
Focus	Not active			Set Dry Heater	180 °C
Scan Begin	50 m/z	Set Capillary	4500 V	Set Dry Gas	4.0 l/min
Scan End	3000 m/z	Set End Plate Offset	-500 V	Set Divert Valve	Source

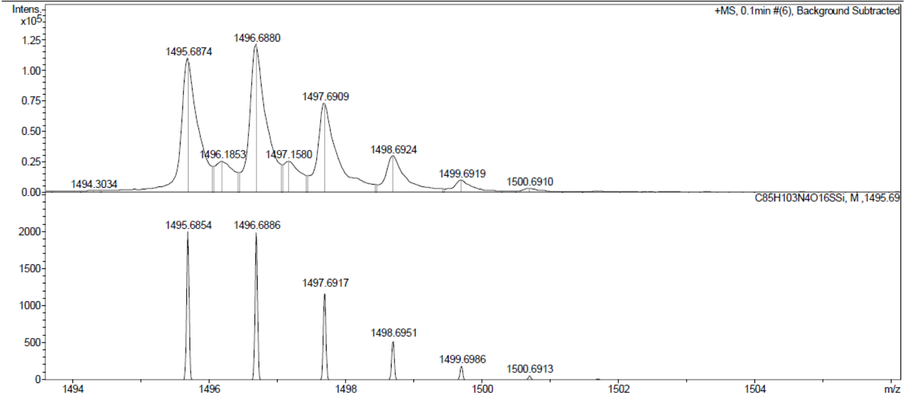

Probe 3 :

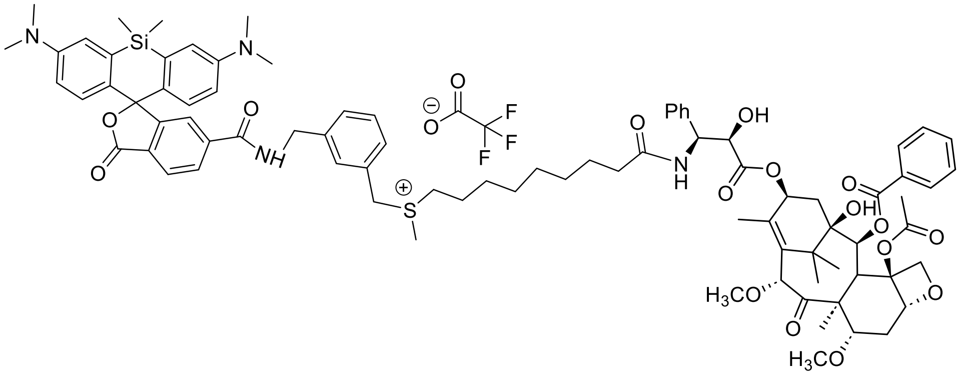

Exact Mass: 1495,69

According to the general procedure, **6-SiR-CO_2H** (2.0 mg, 4.23 µmol), sulfonium **16** (18.3 µL -of a 0.3M solution in DMSO-,5.50 µmol) and **CTX-NH_2.CF_3CO_2H** (10.8 mg, 12.7 µmol) led to the expected product as a blue powder (2.29 µmol, **η = 54 %**).

^{1}H NMR (400 MHz, DMSO-d_6): δ (ppm) = 9.35 (t, *J* = 6.0 Hz, 1H, NH), 8.36 (d, *J* = 9.0 Hz, 1H, NH), 8.13 (dd, *J* = 8.0, 1.5 Hz, 1H, H_{Ar}), 8.06 (d, *J* = 8.0 Hz, 1H, H_{Ar}), 8.01 – 7.95 (m, 2H, H_{Ar}), 7.72 – 7.65 (m, 2H, H_{Ar}), 7.62 – 7.56 (m, 2H, H_{Ar}), 7.42 – 7.31 (m, 8H, H_{Ar}), 7.24 – 7.20 (m, 1H, H_{Ar}), 7.03 (s, 2H, H_{Ar}), 6.66 -6.64 (m, 4H, H_{Ar}), 5.96 – 5.91 (m, 1H, CH), 5.40 (d, *J* = 7.0 Hz, 1H, CH), 5.30 (dd, *J* = 9.0, 5.5 Hz, 1H, CH), 4.98 – 4.95 (m, 1H, CH), 4.71 (s, 1H, CH), 4.69 – 4.57 (m, 2H, CH_2), 4.46 – 4.42 (m, 3H, 1*CH + 1*CH_2), 4.03 (s, 2H, CH_2), 3.76 (dd, *J* = 10.5, 6.5 Hz, 1H, CH), 3.63 (d, *J* = 7.0 Hz, 1H, CH), 3.31 (s, 3H, CH_3), 3.21 (s, 3H, CH_3), 3.20 – 3.12 (m, 2H, CH_2), 2.92 (s, 12H, 4*CH_3), 2.72 (s, 3H, CH_3), 2.70 – 2.60 (m, 1H, 1H from CH_2), 2.26 (s, 3H, CH_3), 2.22 – 2.08 (m, 2H, CH_2), 2.02 – 1.95 (m, 1H, CH_2, 1H from CH_2), 1.90 – 1.83 (m, 4H, 1*CH_3+1H from CH_2), 1.63 – 1.41 (m, 8H, 1*CH_3 + 2*CH_2 + 1H from CH_2), 1.25 – 1.10 (m, 8H, 4*CH_2), 1.03 (s, 3H, CH_3), 0.98 (s, 3H, CH_3), 0.63 (s, 3H, CH_3), 0.52 (s, 3H, CH_3).

^{19}F NMR (376 MHz, DMSO-d_6): δ(ppm) = -74,5.

ESI-MS, positive mode: m/z = 1495.6 [M-$CF_3CO_2^-$]$^+$

HRMS (ESI) calculated for $C_{85}H_{103}N_4O_{16}SSi^+$ [M-$CF_3CO_2^-$]$^+$: 1495.6854, found: 1495.6861.

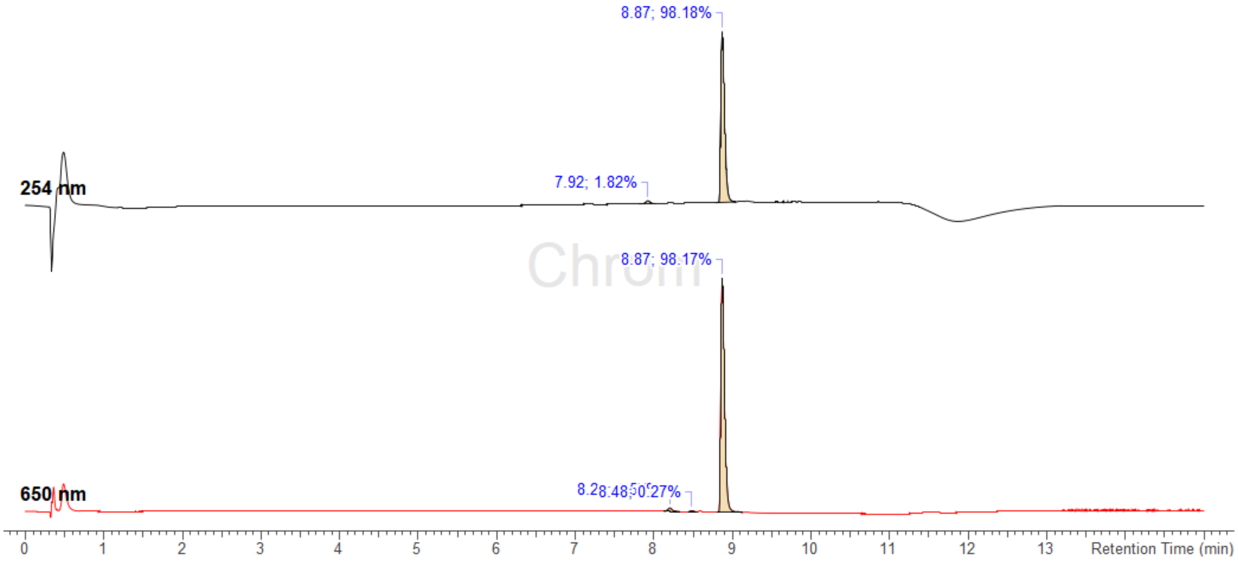

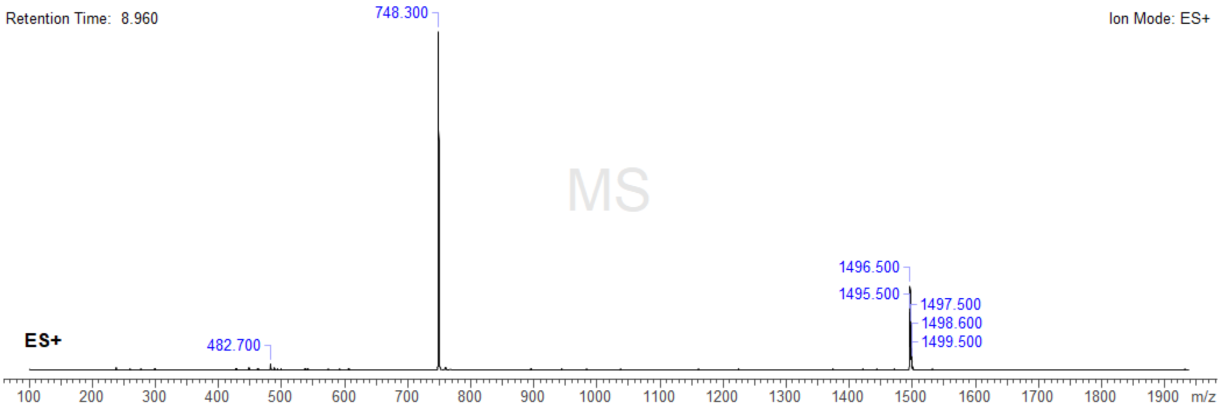

**Analysis Info**		Acquisition Date	04.09.2024 23:06:27
Analysis Name	Z:\Data\2024\2409\sam040924\MA278_31_01_127437.d		
Method	hystar_p.m	Operator	BDAL@DE
Sample Name	MA278	Instrument / Ser#	micrOTOF 10237
Comment			

Acquisition Parameter

Source Type	ESI	Ion Polarity	Positive	Set Nebulizer	0.4 Bar
Focus	Not active			Set Dry Heater	180 °C
Scan Begin	50 m/z	Set Capillary	4500 V	Set Dry Gas	4.0 l/min
Scan End	3000 m/z	Set End Plate Offset	-500 V	Set Divert Valve	Source

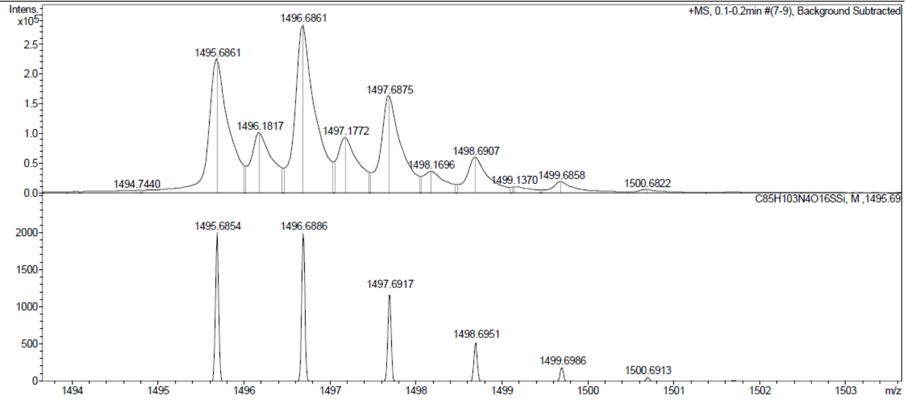

Probe 4 :

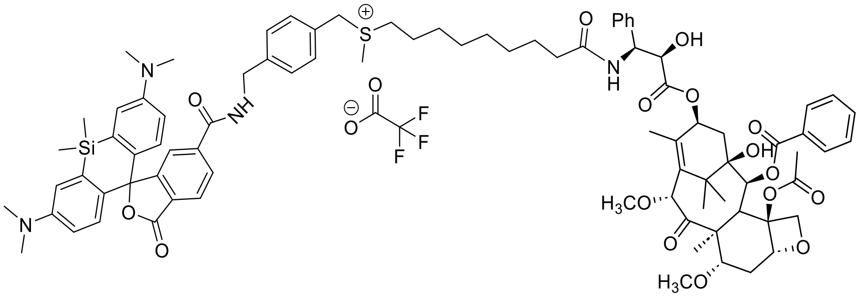

Exact Mass: 1495,68

According to the general procedure, **6-SiR-CO_2H** (2.0 mg, 4.23 µmol), sulfonium **17** (19.6 µL -of a 0.28M solution in DMSO-,5.50 µmol) and **CTX-NH_2.CF_3CO_2H** (10.8 mg, 12.7 µmol) led to the expected product as a blue powder (2.46 µmol, **η = 58 %**).

^{1}H NMR (400 MHz, DMSO-d$_6$): δ (ppm) = 9.35 (t, *J* = 6.0 Hz, 1H, NH), 8.36 (d, *J* = 9.0 Hz, 1H, NH), 8.15 – 8.11 (m, 1H, H_{Ar}), 8.06 (d, *J* = 8.0 Hz, 1H, H_{Ar}), 8.00 – 7.96 (m, 2H, H_{Ar}), 7.70 – 7.66 (m, 2H, H_{Ar}), 7.62 – 7.56 (m, 2H, H_{Ar}), 7.42 – 7.31 (m, 8H, H_{Ar}), 7.25 – 7.20 (m, 1H, H_{Ar}), 7.03 (s, 2H, H_{Ar}), 6.68 -6.63 (m, 4H, H_{Ar}), 5.99 – 5.89 (m, 1H, CH),5.40 (d, *J* = 7.0 Hz, 1H, CH), 5.30 (dd, *J* = 9.0, 5.5 Hz, 1H, CH), 4.96 (dd, *J* = 9.5, 2.0 Hz, 1H, CH), 4.71 (s, 1H, CH), 4.67 (d, *J* = 13.0 Hz, 1H, 1H from CH_2), 4.59 (d, *J* = 13.0 Hz, 1H, 1H from CH_2), 4.46 (d, *J* = 6.0 Hz, 2H, CH_2), 4.43 (d, *J* = 5.5 Hz, 1H, CH), 4.03 (s, 2H, CH_2), 3.76 (dd, *J* = 10.5, 6.5 Hz, 1H, CH), 3.63 (d, *J* = 7.0 Hz, 1H, CH), 3.31 (s, 3H, CH_3), 3.23 – 3.13 (m, 5H, 1*CH_2 + 1*CH_3), 2.93 (s, 12H, 4*CH_3), 2.73 (s, 3H, CH_3), 2.67 – 2.62 (m, 1H, 1H from CH_2), 2.26 (s, 3H, CH_3), 2.20 – 2.12 (m, 2H, CH_2), 2.00 – 1.95 (m, 1H, 1H from CH_2), 1.90 – 1.81 (m, 4H, 1*CH_3 + 1H from CH_2),1.64 – 1.44 (m, 8H, 1*CH_3 + 2*CH_2 + 1H from CH_2), 1.27 – 1.14 (m, 8H, 4*CH_2), 1.03 (s, 3H, CH_3), 0.98 (s, 3H, CH_3), 0.63 (s, 3H, CH_3), 0.52 (s, 3H, CH_3).

^{19}F NMR (376 MHz, DMSO-d$_6$): δ(ppm) = -74,5.

ESI-MS, positive mode: m/z = 1495.5 [M-$CF_3CO_2^-$]$^+$

HRMS (ESI) calculated for $C_{85}H_{103}N_4O_{16}SSi^+$ [M-$CF_3CO_2^-$]$^+$: 1495.6854, found: 1495.6857.

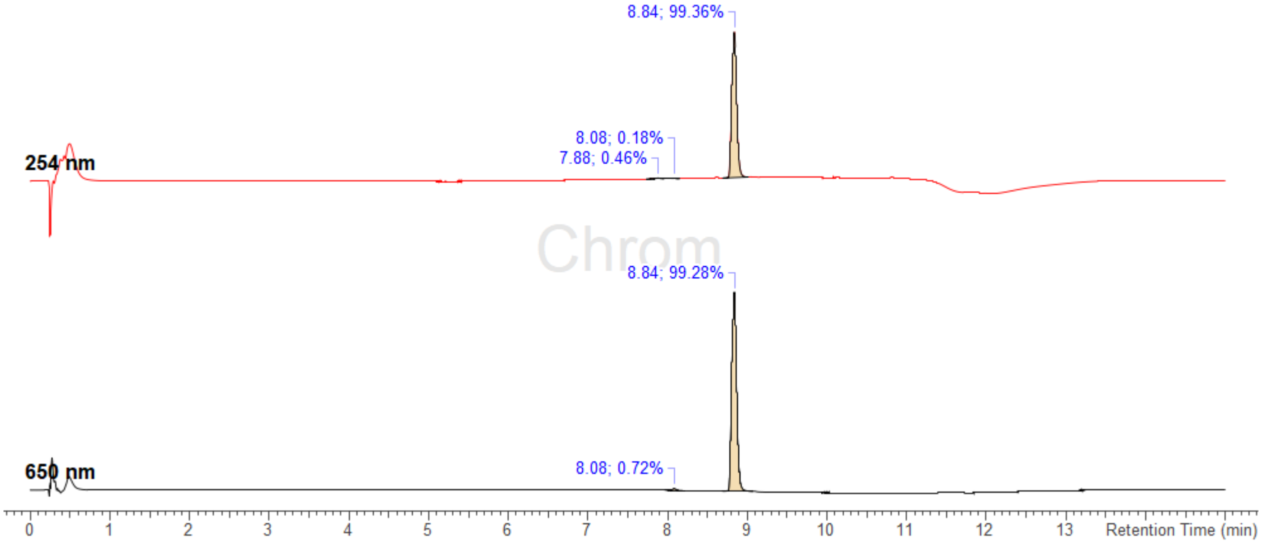

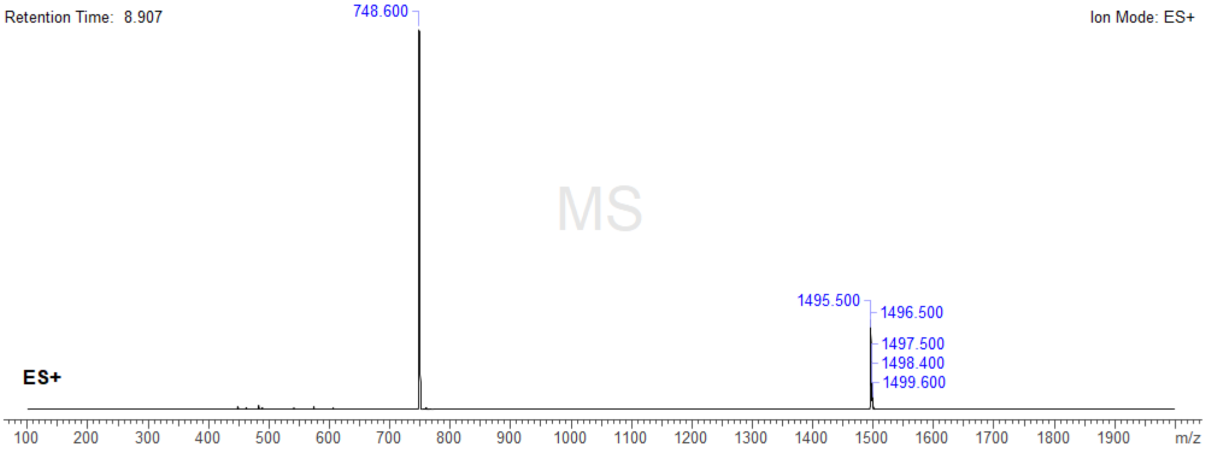

Analysis Info

Analysis Name	Z:\Data\2024\2409\sam040924\MA279_29_01_127435.d	Acquisition Date	04.09.2024 22:57:09
Method	hystar_p.m	Operator	BDAL@DE
Sample Name	MA279	Instrument / Ser#	micrOTOF 10237
Comment			

Acquisition Parameter

Source Type	ESI	Ion Polarity	Positive	Set Nebulizer	0.4 Bar
Focus	Not active			Set Dry Heater	180 °C
Scan Begin	50 m/z	Set Capillary	4500 V	Set Dry Gas	4.0 l/min
Scan End	3000 m/z	Set End Plate Offset	-500 V	Set Divert Valve	Source

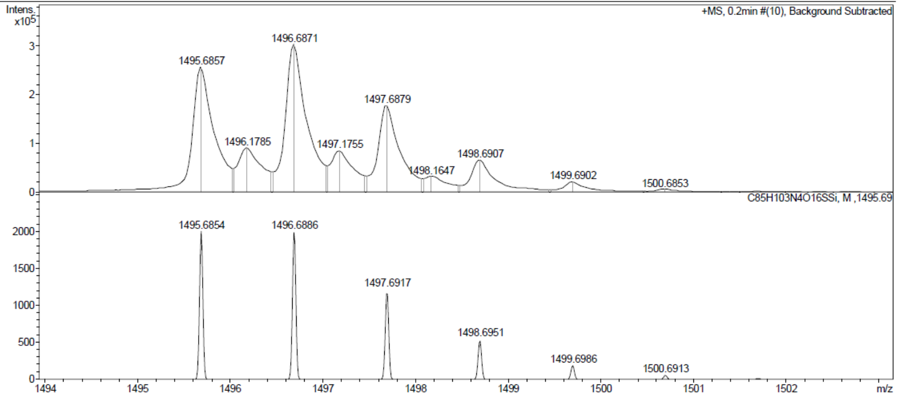

Probe 5 :

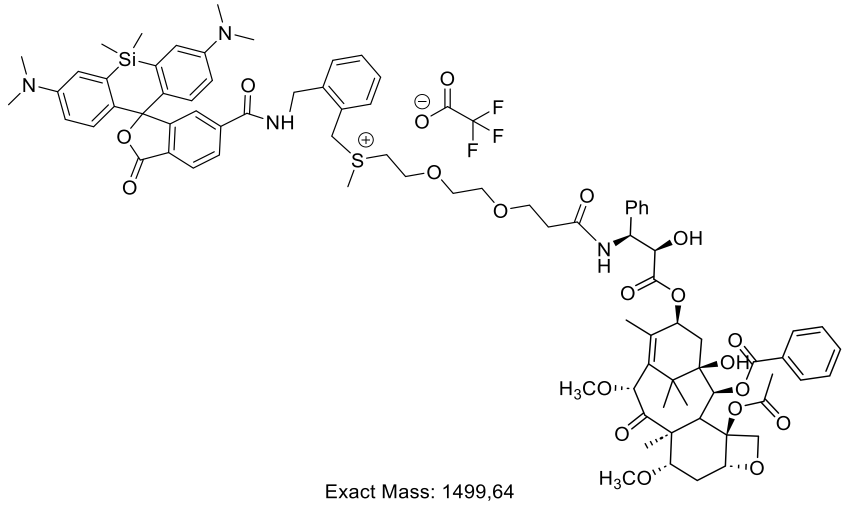

Exact Mass: 1499,64

According to the general procedure, **6-SiR-CO_2H** (2.0 mg, 4.23 µmol), sulfonium **18** (37.7 µL -of a 0.146 M solution in DMSO-,5.50 µmol) and **CTX-NH_2.CF_3CO_2H** (10.8 mg, 12.7 µmol) led to the expected product as a blue powder (2.26 µmol, **η = 53 %**).

^{1}H NMR (400 MHz, DMSO-d_6): δ (ppm) = 9.34 (t, *J* = 5.5 Hz, 1H, NH), 8.46 (d, *J* = 9.0 Hz, 1H, NH), 8.13 (dd, *J* = 8.0, 1.5 Hz, 1H, H_{Ar}), 8.07 (d, *J* = 8.0 Hz, 1H, H_{Ar}), 8.00 – 7.96 (m, 2H, H_{Ar}), 7.72 – 7.65 (m, 2H, H_{Ar}), 7.62 – 7.56 (m, 2H, H_{Ar}), 7.45 – 7.40 (m, 3H, H_{Ar}), 7.38 – 7.29 (m, 5H, H_{Ar}), 7.23 – 7.18 (m, 1H, H_{Ar}), 7.03 (s, 2H, H_{Ar}), 6.64 (s, 4H, H_{Ar}), 5.97 – 5.92 (m, 1H, CH), 5.39 (d, *J* = 7.0 Hz, 1H, CH), 5.28 (dd, *J* = 9.0, 5.5 Hz, 1H, CH), 4.97 – 4.94 (m, 1H, CH), 4.91 (d, *J* = 13.0 Hz, 1H, 1H from CH_2), 4.77 (d, *J* = 13.0 Hz, 1H, 1H from CH_2), 4.70 (s, 1H, CH), 4.55 (d, *J* = 5.5 Hz, 2H, CH_2), 4.43 (d, *J* = 6.0 Hz, 1H, CH), 4.05 – 4.00 (m, 2H, CH_2), 3.84 – 3.68 (m, 3H, CH_2 + CH), 3.63 (d, *J* = 7.0 Hz, 1H, CH), 3.57 – 3.41 (m, 8H, 4*CH_2), 3.30 (s, 3H, CH_3), 3.21 (s, 3H, CH_3), 2.92 (s, 12H, 4*CH_3), 2.83 (s, 3H, CH_3), 2.69 – 2.61 (m, 1H, 1H from CH_2), 2.45 – 2.38 (m, 2H, CH_2), 2.23 (s, 3H, CH_3), 1.96 (dd, *J* = 15.0, 9.0 Hz, 1H, 1H from CH_2), 1.85 – 1.79 (m, 4H, 1*CH_3 + 1H from CH_2), 1.54 – 1.46 (m, 4H, 1*CH_3 + 1H from CH_2), 1.02 (s, 3H, CH_3), 0.98 (s, 3H, CH_3), 0.62 (s, 3H, CH_3), 0.52 (s, 3H, CH_3).

^{19}F NMR (376 MHz, DMSO-d_6): δ(ppm) = -74.5.

ESI-MS, positive mode: m/z = 1499.6 [M-$CF_3CO_2^-$]$^+$

HRMS (ESI) calculated for $C_{83}H_{99}N_4O_{18}SSi^+$ [M-$CF_3CO_2^-$]$^+$: 1499.6439, found: 1499.6442.

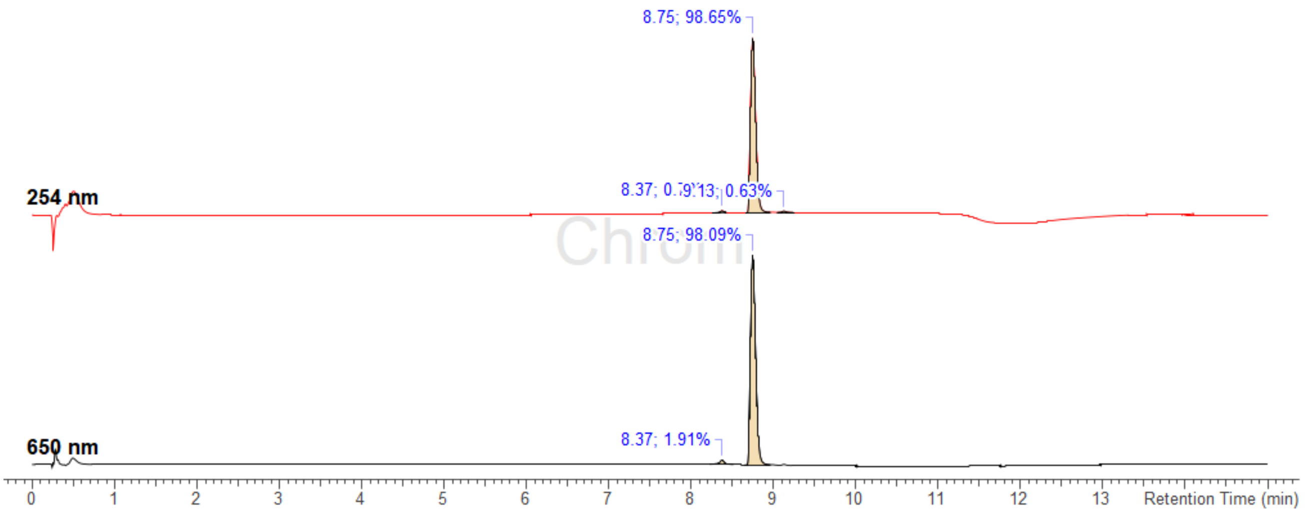

Retention Time: 8.829 Ion Mode: ES+

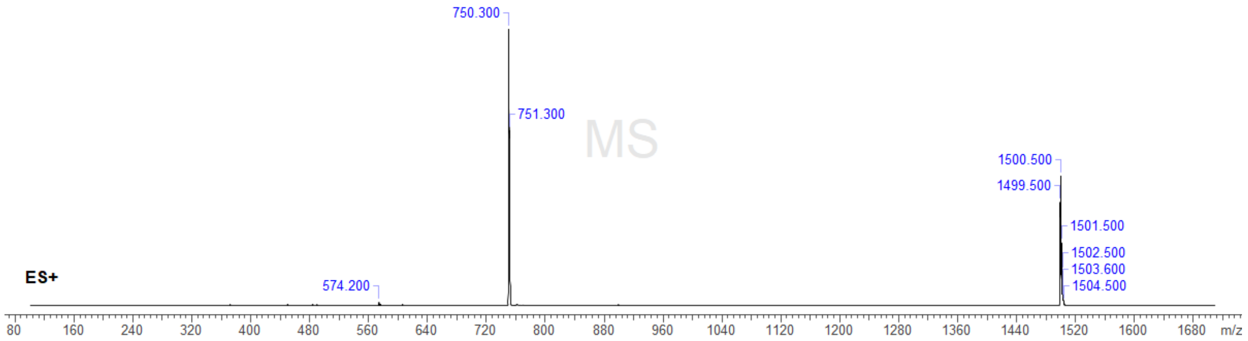

Analysis Info

Analysis Name	Z:\Data\2024\2409\sam040924\MA244_27_01_127433.d	Acquisition Date	04.09.2024 22:47:54	
Method	hystar_p.m	Operator	BDAL@DE	
Sample Name	MA244	Instrument / Ser#	micrOTOF	10237
Comment				

Acquisition Parameter

Source Type	ESI	Ion Polarity	Positive	Set Nebulizer	0.4 Bar
Focus	Not active			Set Dry Heater	180 °C
Scan Begin	50 m/z	Set Capillary	4500 V	Set Dry Gas	4.0 l/min
Scan End	3000 m/z	Set End Plate Offset	-500 V	Set Divert Valve	Source

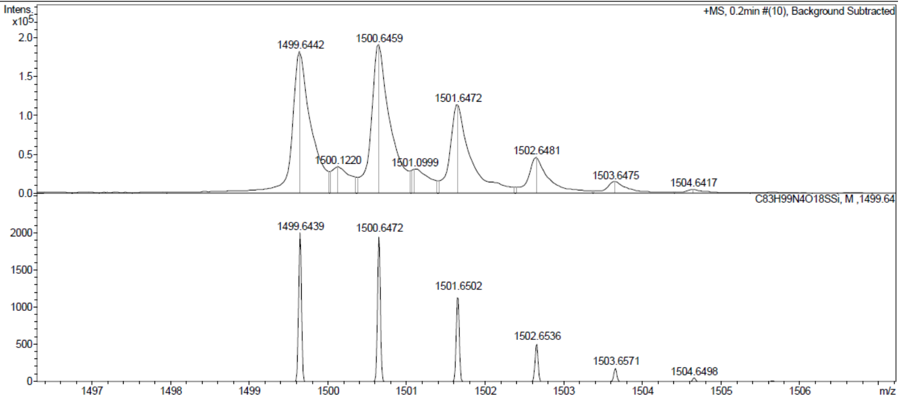

Probe 6 :

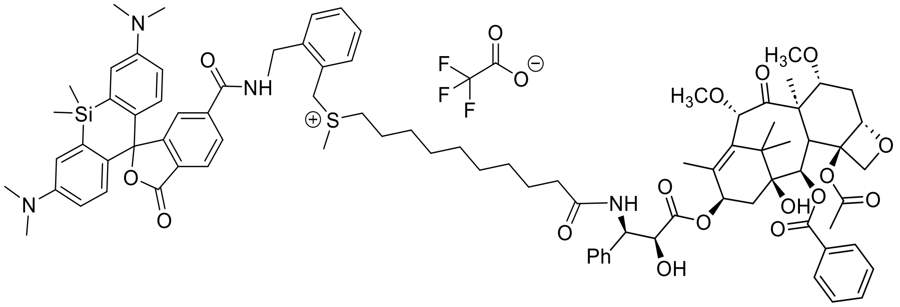

Exact Mass: 1509,70

According to the general procedure, **6-SiR-CO_2H** (2.0 mg, 4.23 µmol), sulfonium **18** (71.4 µL -of a 77 mM solution in DMSO-,5.50 µmol) and **CTX-NH_2.CF_3CO_2H** (10.8 mg, 12.7 µmol) led to the expected product as a blue powder (1.48 µmol, **η = 35 %**).

^{1}H NMR (400 MHz, DMSO-d_6): δ (ppm) = 9.33 (t, *J* = 5.5 Hz, 1H, NH), 8.35 (d, *J* = 9.0 Hz, 1H, NH), 8.13 (dd, *J* = 8.0, 1.5 Hz, 1H, , H_{Ar}), 8.06 (d, *J* = 8.0 Hz, 1H, H_{Ar}), 8.00 – 7.96 (m, 2H, , H_{Ar}), 7.72 – 7.66 (m, 2H, H_{Ar}), 7.62 – 7.57 (m, 2H,H_{Ar}), 7.46 – 7.40 (m, 3H, H_{Ar}), 7.39 – 7.31 (m, 5H, H_{Ar}), 7.24 – 7.20 (m, 1H, H_{Ar}), 7.02 (s, 2H, H_{Ar}), 6.63 (s, 4H, H_{Ar}), 5.96 -5.91 (m, 1H, CH), 5.40 (d, *J* = 7.0 Hz, 1H, CH), 5.32 – 5.28 (m, 1H, CH), 4.98 – 4.94 (m, 1H, CH), 4.86 (d, *J* = 13.0 Hz, 1H, 1H from CH_2), 4.76 (d, *J* = 13.0 Hz, 1H, 1H from CH_2), 4.71 (s, 1H, CH), 4.64 (s, 1H, OH), 4.57 – 4.54 (m, 2H, CH_2), 4.43 (d, *J* = 5.5 Hz, 1H, CH), 4.03 (s, 2H, CH_2), 3.76 (dd, *J* = 10.5, 6.5 Hz, 1H, CH), 3.63 (d, *J* = 7.0 Hz, 1H, CH), 3.33 – 3.27 (m, 5H, 1*CH_2 + 1*CH_3), 3.21 (s, 3H, CH_3), 2.92 (s, 12H, 4*CH_3), 2.84 (s, 3H, CH_3), 2.69 – 2.64 (m, 1H, 1H from CH_2), 2.26 (s, 3H, CH_3), 2.19 – 2.11 (m, 2H, CH_2), 2.01 – 1.95 (m, 1H, 1H from CH_2), 1.92 – 1.82 (m, 4H, 1*CH_3 + 1H from CH_2), 1.64 – 1.40 (m, 8H, 1*CH_3 + 2*CH_2 + 1H from CH_2), 1.23 – 1.13 (m, 10H, 5*CH_2), 1.03 (s, 3H, CH_3), 0.98 (s, 3H, CH_3), 0.62 (s, 3H, CH_3), 0.52 (s, 3H, CH_3).

^{19}F NMR (376 MHz, DMSO-d_6): δ(ppm) = -74,3.

ESI-MS, positive mode: m/z = 1509.5 [M-$CF_3CO_2^-$]$^+$

HRMS (ESI) calculated for $C_{86}H_{105}N_4O_{16}SSi^+$ [M-$CF_3CO_2^-$]$^+$: 1509.7010, found: 1509.7014.

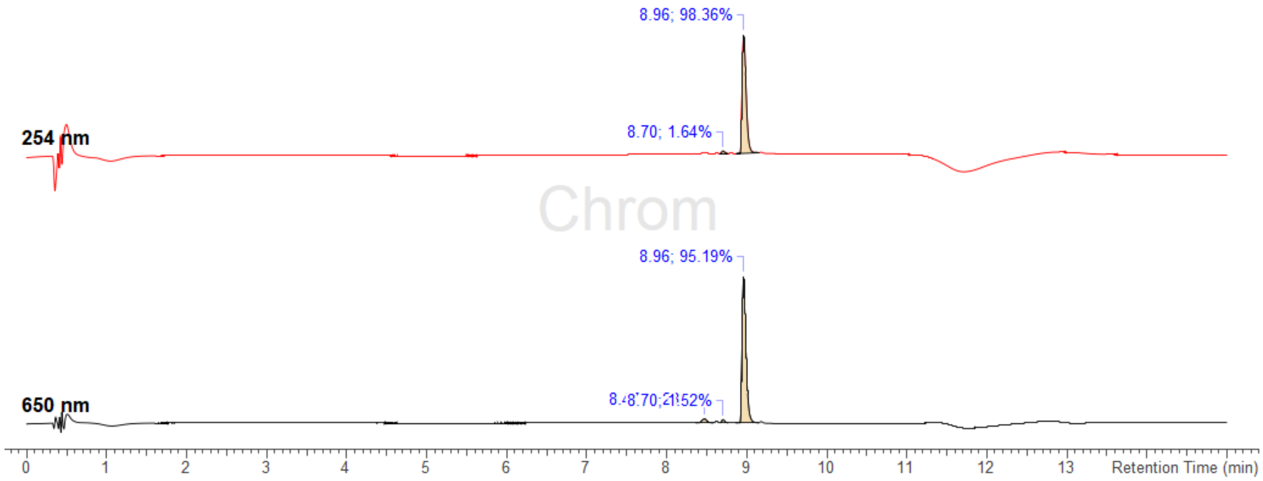

Retention Time: 9.038 Ion Mode: ES+

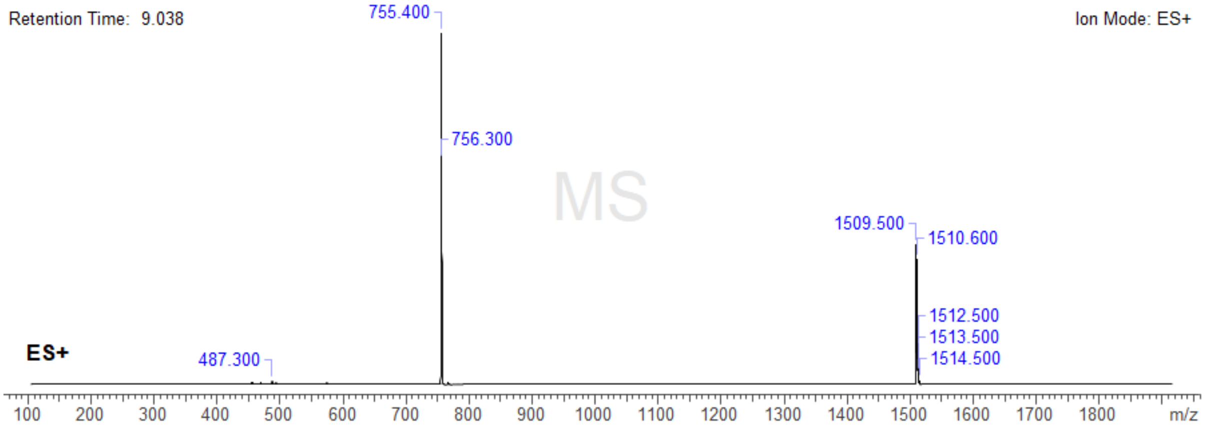

Analysis Info

Analysis Name	Z:\Data\2024\2409\sam040924\MA282_28_01_127434.d	Acquisition Date	04.09.2024 22:52:31
Method	hystar_p.m	Operator	BDAL@DE
Sample Name	MA282	Instrument / Ser#	micrOTOF 10237
Comment			

Acquisition Parameter

Source Type	ESI	Ion Polarity	Positive	Set Nebulizer	0.4 Bar
Focus	Not active			Set Dry Heater	180 °C
Scan Begin	50 m/z	Set Capillary	4500 V	Set Dry Gas	4.0 l/min
Scan End	3000 m/z	Set End Plate Offset	-500 V	Set Divert Valve	Source

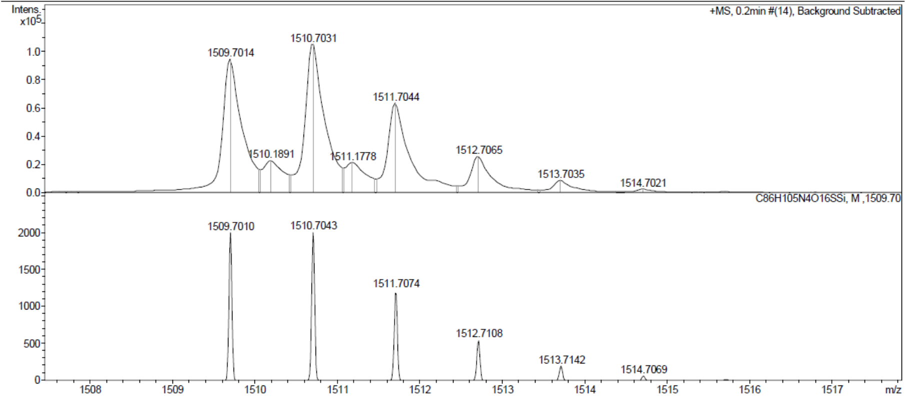

Supplementary NMR spectra

^{1}H NMR of ***p*-7**:

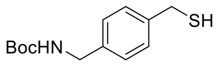

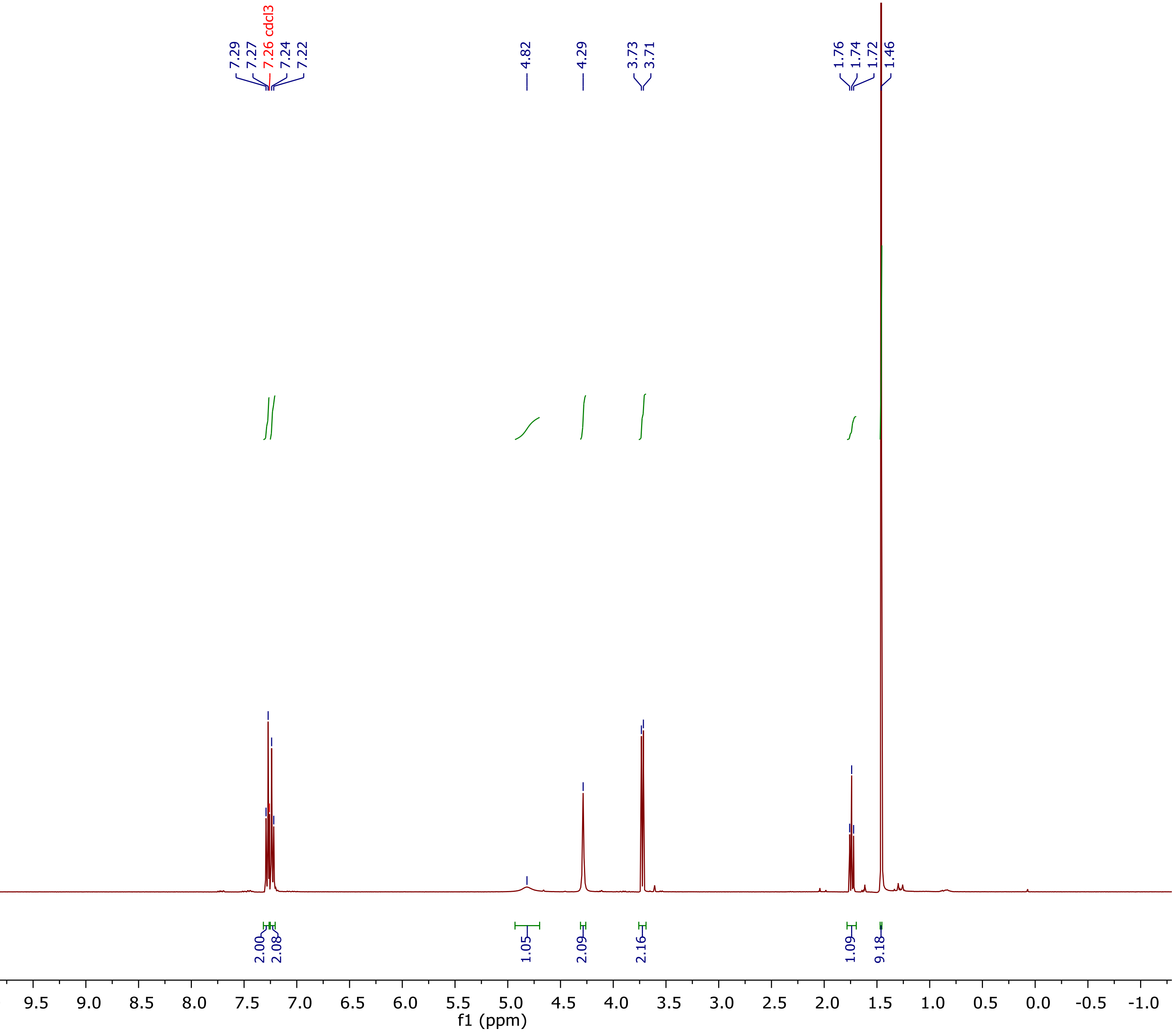

^{13}C NMR of ***p*-7:**

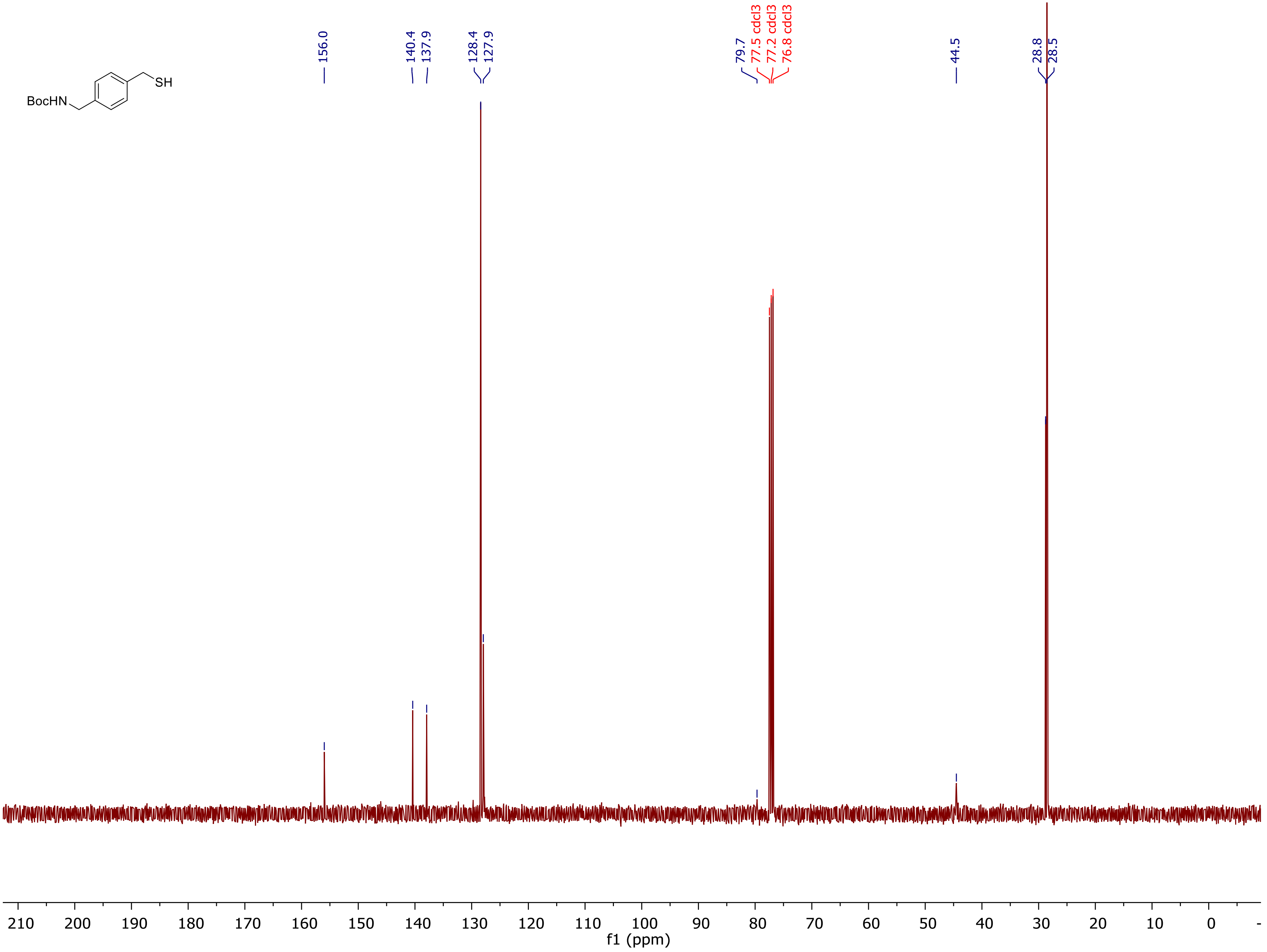

BocHN
SH
156.0
140.4
137.9
128.4
127.9
79.7
77.5 cdcl3
77.2 cdcl3
76.8 cdcl3
44.5
28.8
28.5
210
200
190
180
170
160
150
140
130
120
110
100
90
80
70
60
50
40
30
20
10
0
f1 (ppm)

^{1}H NMR of ***m*-7**:

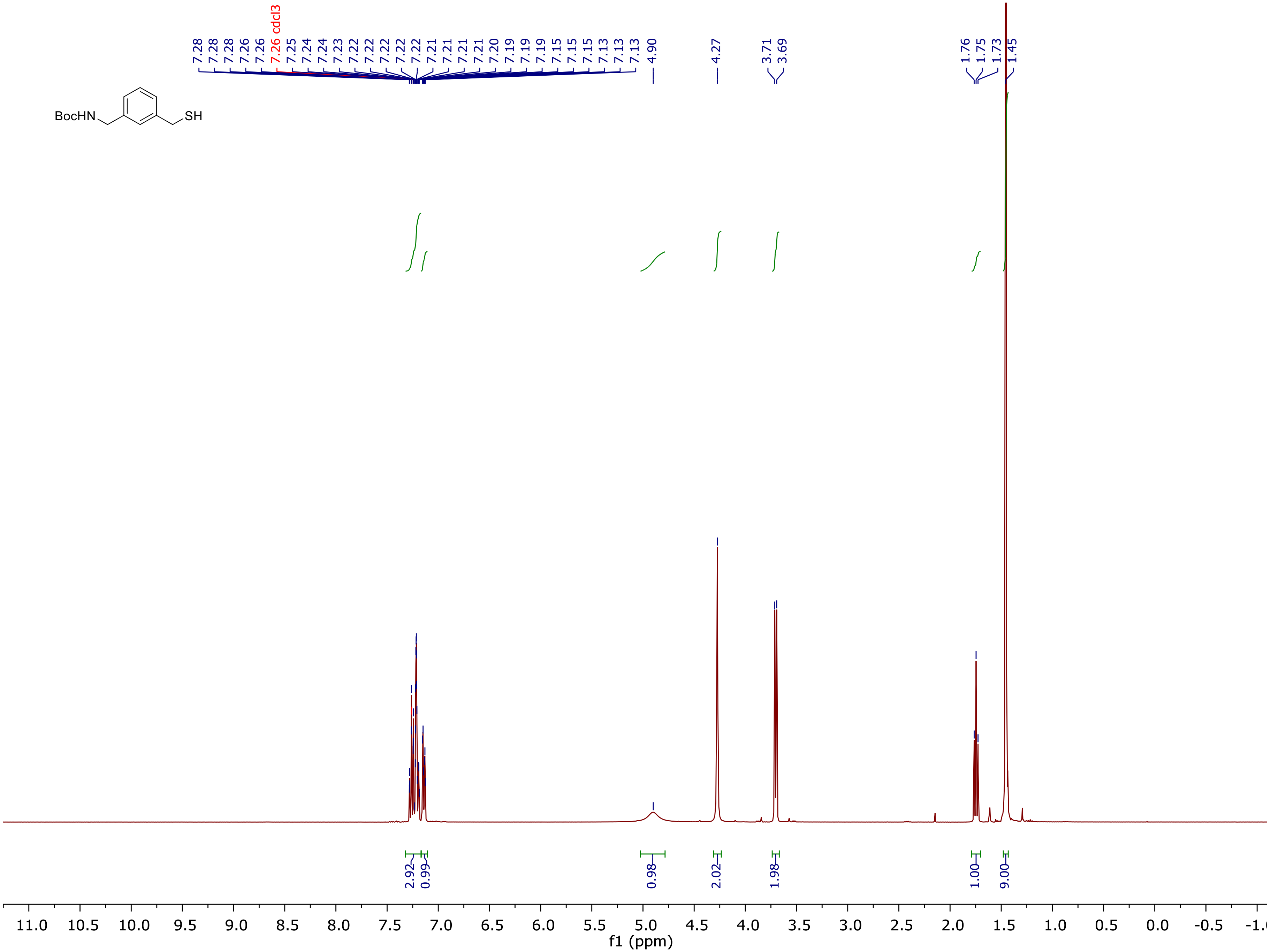

BocHN
SH
7.28
7.28
7.28
7.26
7.26
7.26 cdcl3
7.25
7.24
7.24
7.23
7.22
7.22
7.22
7.22
7.22
7.21
7.21
7.21
7.21
7.20
7.19
7.19
7.19
7.15
7.15
7.15
7.13
7.13
7.13
4.90
4.27
3.71
3.69
1.76
1.75
1.73
1.45
2.92
0.99
0.98
2.02
1.98
1.00
9.00
11.0
10.5
10.0
9.5
9.0
8.5
8.0
7.5
7.0
6.5
6.0
5.5
5.0
4.5
4.0
3.5
3.0
2.5
2.0
1.5
1.0
0.5
0.0
-0.5
f1 (ppm)

^{13}C NMR of ***m*-7**:

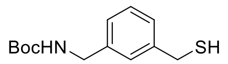
BocHN
SH

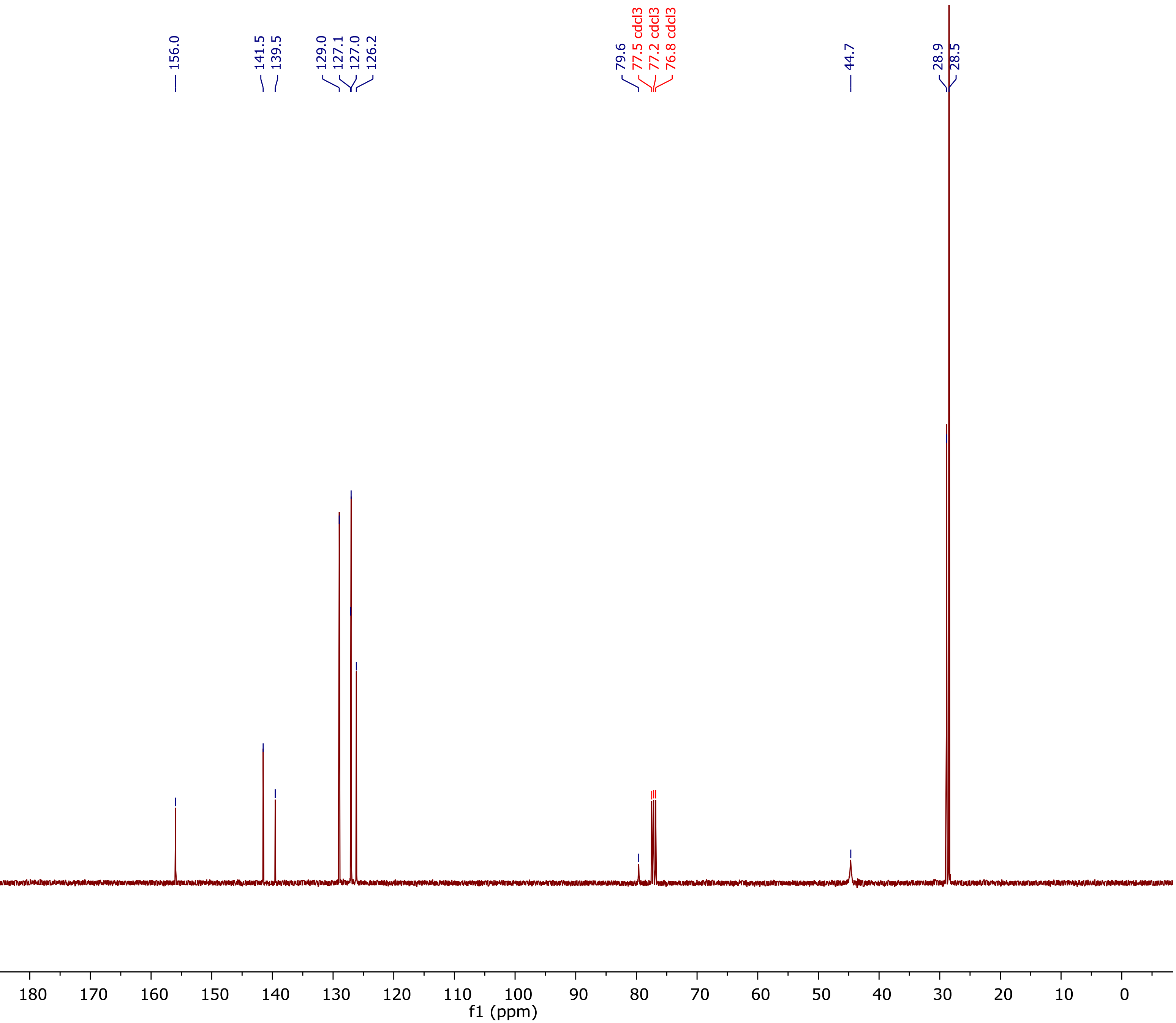
156.0
141.5
139.5
129.0
127.1
127.0
126.2
79.6
77.5 cdcl3
77.2 cdcl3
76.8 cdcl3
44.7
28.9
28.5
210
200
190
180
170
160
150
140
130
120
110
100
90
80
70
60
50
40
30
20
10
0
f1 (ppm)

^{1}H NMR of ***o*-7:**

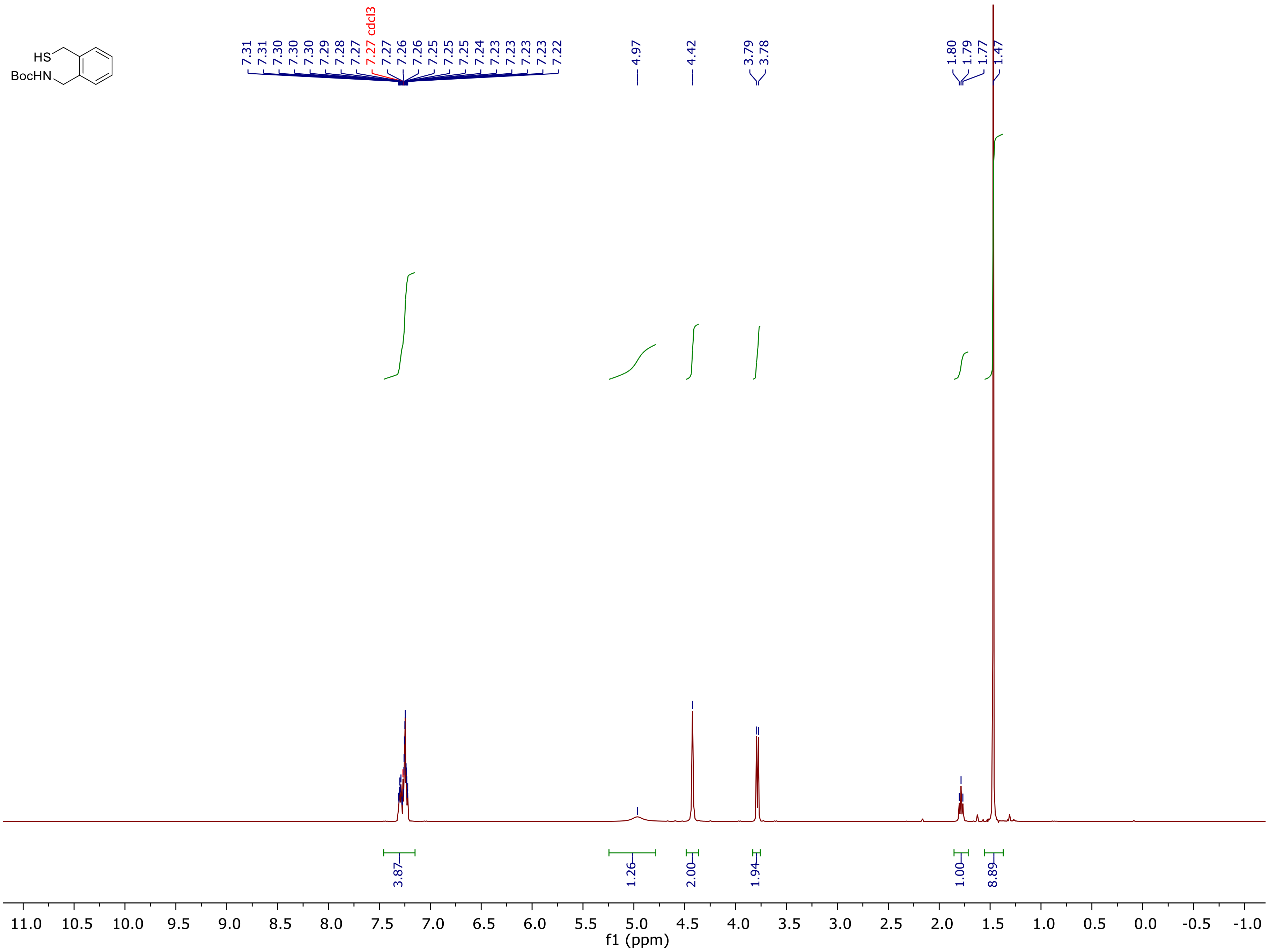
HS
BocHN
7.31
7.31
7.30
7.30
7.30
7.29
7.28
7.27
7.27 cdcl3
7.27
7.26
7.26
7.25
7.25
7.25
7.24
7.23
7.23
7.23
7.23
7.22
4.97
4.42
3.79
3.78
1.80
1.79
1.77
1.47
3.87
1.26
2.00
1.94
1.00
8.89
11.0
10.5
10.0
9.5
9.0
8.5
8.0
7.5
7.0
6.5
6.0
5.5
5.0
4.5
4.0
3.5
3.0
2.5
2.0
1.5
1.0
0.5
0.0
-0.5
-1.0
f1 (ppm)

^{13}C NMR of ***o*-7:**

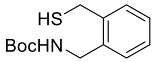
HS
BocHN

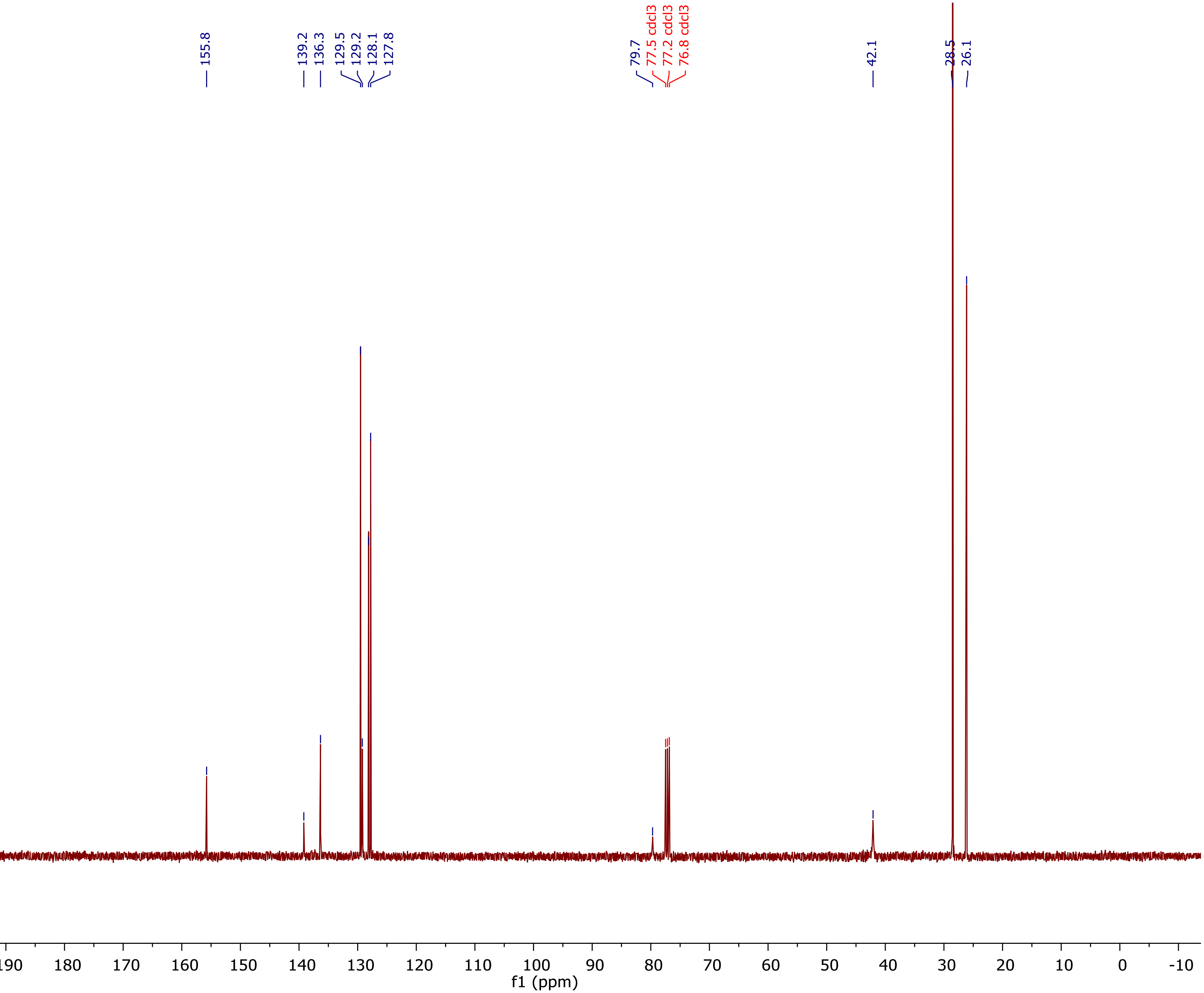
155.8
139.2
136.3
129.5
129.2
128.1
127.8
79.7
77.5 cdcl3
77.2 cdcl3
76.8 cdcl3
42.1
28.5
26.1
210
200
190
180
170
160
150
140
130
120
110
100
90
80
70
60
50
40
30
20
10
0
-10
f1 (ppm)

^{1}H NMR of **8:**

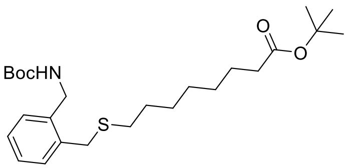
BocHN
S
O
O

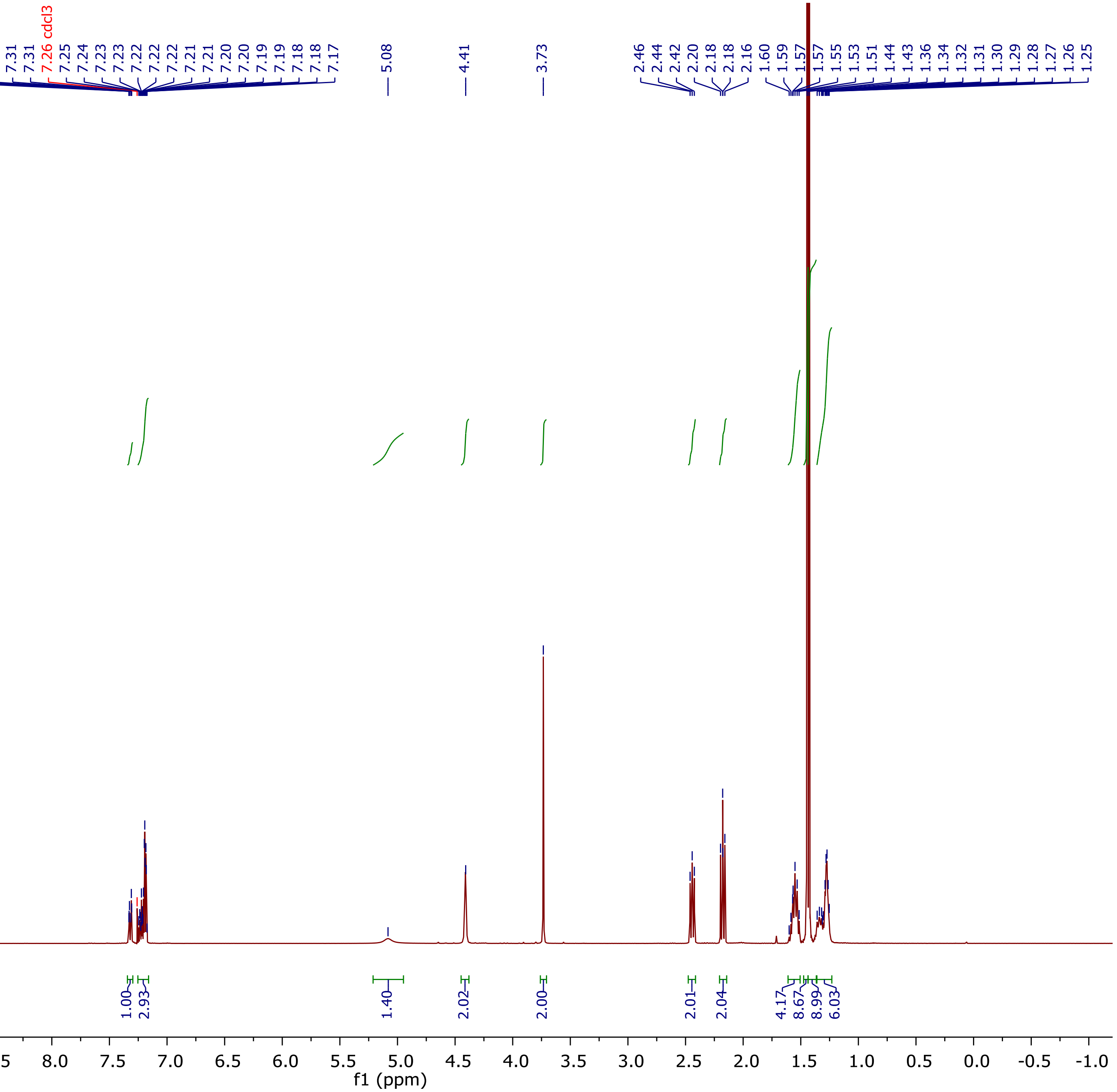
7.33
7.33
7.33
7.31
7.31
7.31
7.26 cdcl3
7.25
7.24
7.23
7.23
7.22
7.22
7.22
7.21
7.21
7.20
7.20
7.19
7.19
7.18
7.18
7.17
5.08
4.41
3.73
2.46
2.44
2.42
2.20
2.18
2.18
2.16
1.60
1.59
1.57
1.57
1.55
1.53
1.51
1.44
1.43
1.36
1.34
1.32
1.31
1.30
1.29
1.28
1.27
1.26
1.25
1.00
2.93
1.40
2.02
2.00
2.01
2.04
4.17
8.67
8.99
6.03
11.0
10.5
10.0
9.5
9.0
8.5
8.0
7.5
7.0
6.5
6.0
5.5
5.0
4.5
4.0
3.5
3.0
2.5
2.0
1.5
1.0
0.5
0.0
-0.5
-1.0
f1 (ppm)

^{13}C NMR of **8:**

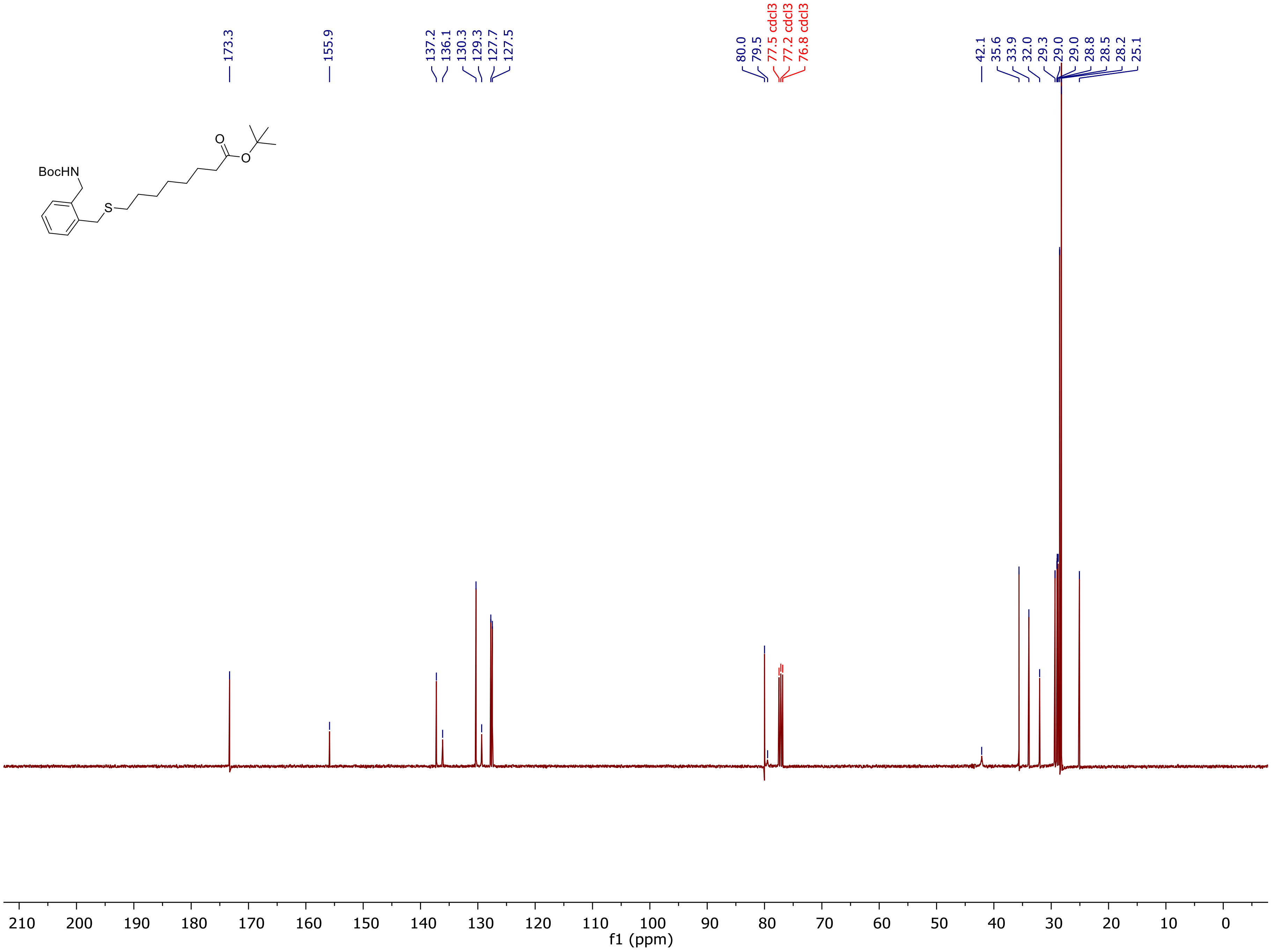

173.3
155.9
137.2
136.1
130.3
129.3
127.7
127.5
80.0
79.5
77.5 cdcl3
77.2 cdcl3
76.8 cdcl3
42.1
35.6
33.9
32.0
29.3
29.0
29.0
28.8
28.5
28.2
25.1
BocHN
S
O
O
210
200
190
180
170
160
150
140
130
120
110
100
90
80
70
60
50
40
30
20
10
0
f1 (ppm)

^{1}H NMR of **9:**

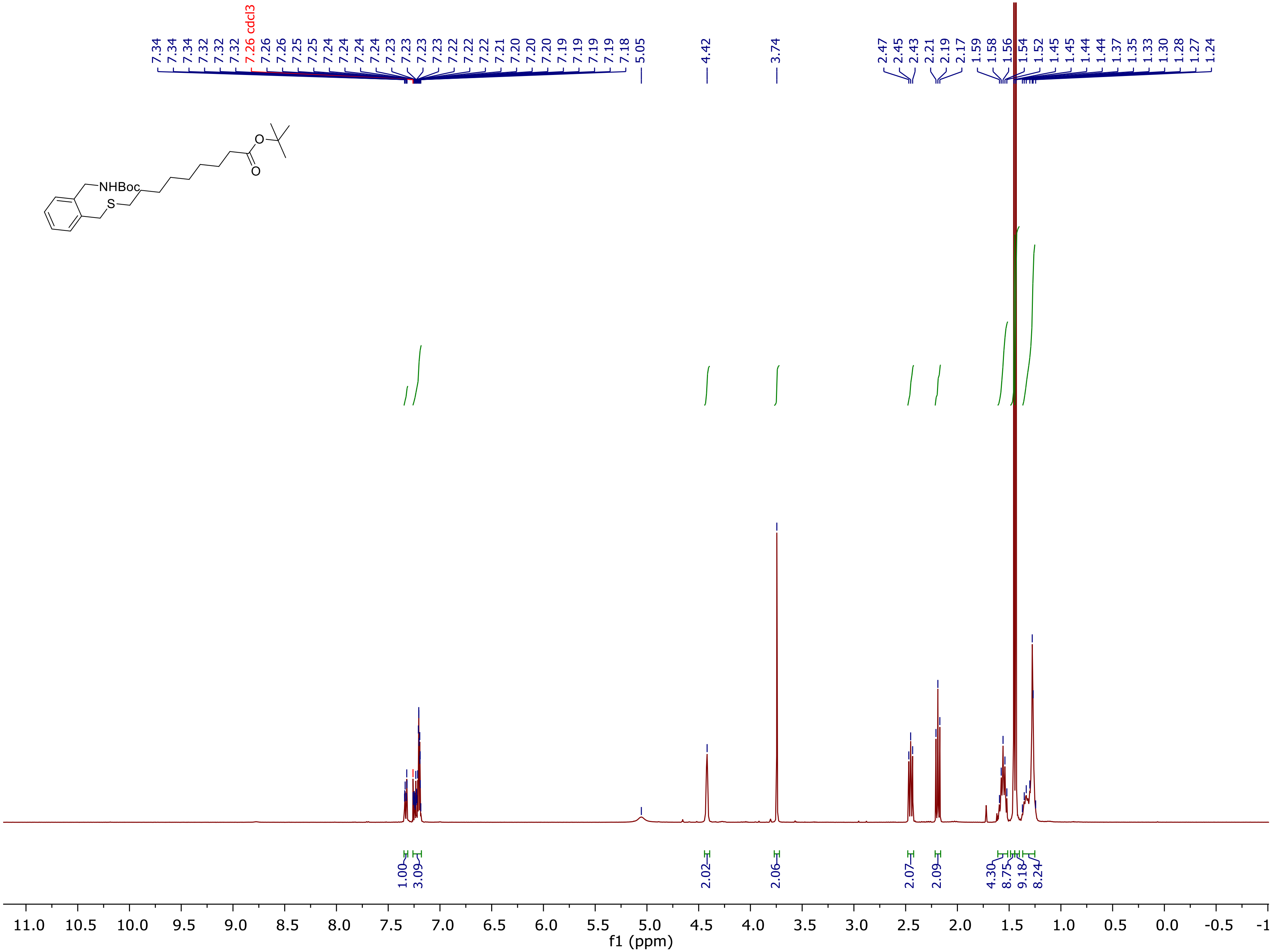

NHBoc
S
O
O
7.34
7.34
7.34
7.32
7.32
7.32
7.26 cdcl3
7.26
7.26
7.25
7.25
7.24
7.24
7.24
7.24
7.23
7.23
7.23
7.23
7.22
7.22
7.22
7.21
7.20
7.20
7.20
7.19
7.19
7.19
7.19
7.18
5.05
4.42
3.74
2.47
2.45
2.43
2.21
2.19
2.17
1.59
1.58
1.56
1.54
1.52
1.45
1.45
1.44
1.44
1.37
1.35
1.33
1.30
1.28
1.27
1.24
1.00
3.09
2.02
2.06
2.07
2.09
4.30
8.75
9.18
8.24
11.0
10.5
10.0
9.5
9.0
8.5
8.0
7.5
7.0
6.5
6.0
5.5
5.0
4.5
4.0
3.5
3.0
2.5
2.0
1.5
1.0
0.5
0.0
-0.5
-1
f1 (ppm)

^{13}C NMR of **9:**

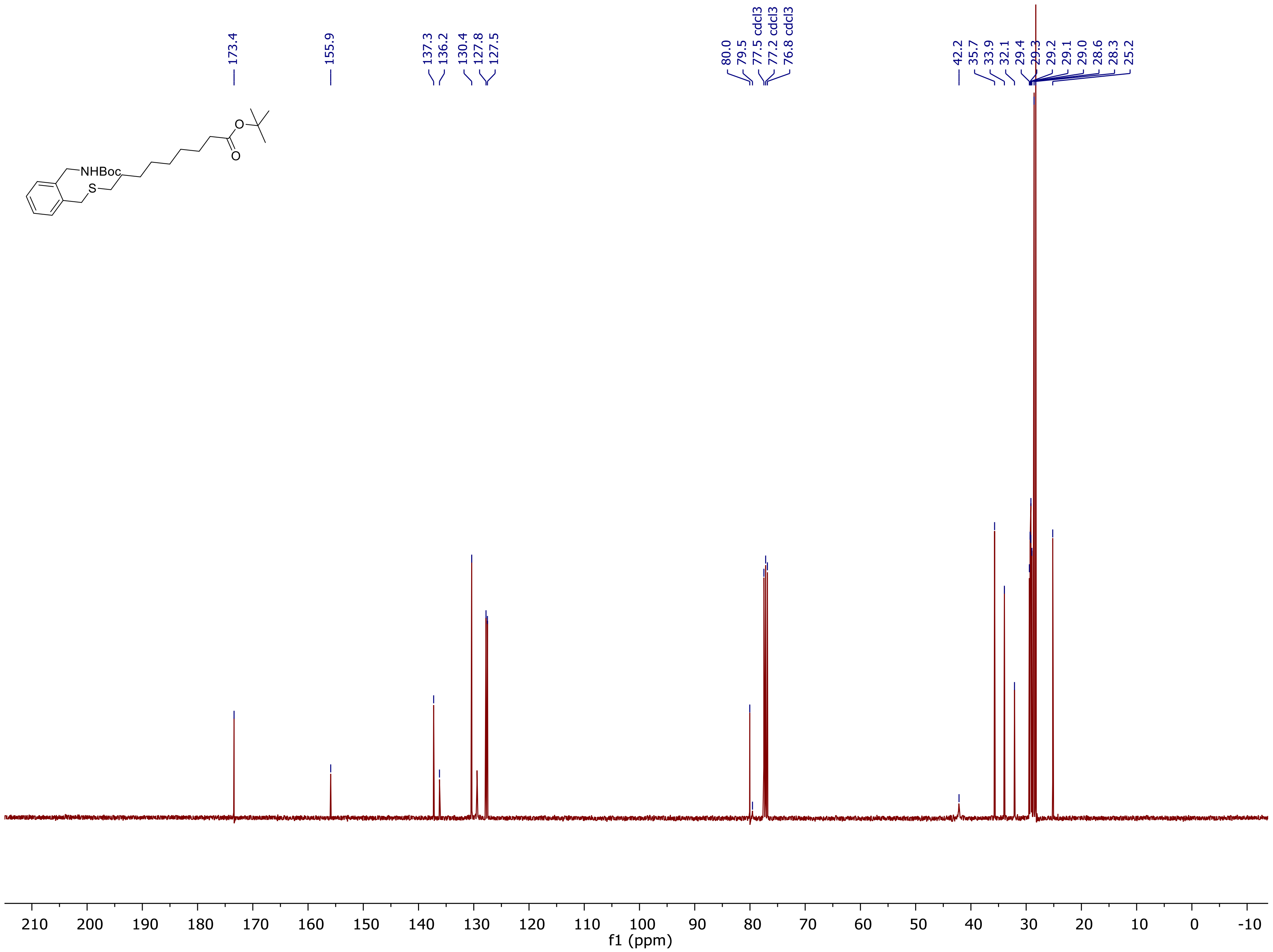
NHBoc
S
O
O
173.4
155.9
137.3
136.2
130.4
127.8
127.5
80.0
79.5
77.5 cdcl3
77.2 cdcl3
76.8 cdcl3
42.2
35.7
33.9
32.1
29.4
29.3
29.2
29.1
29.0
28.6
28.3
25.2
210
200
190
180
170
160
150
140
130
120
110
100
90
80
70
60
50
40
30
20
10
0
-10
f1 (ppm)

^{1}H NMR of **10:**

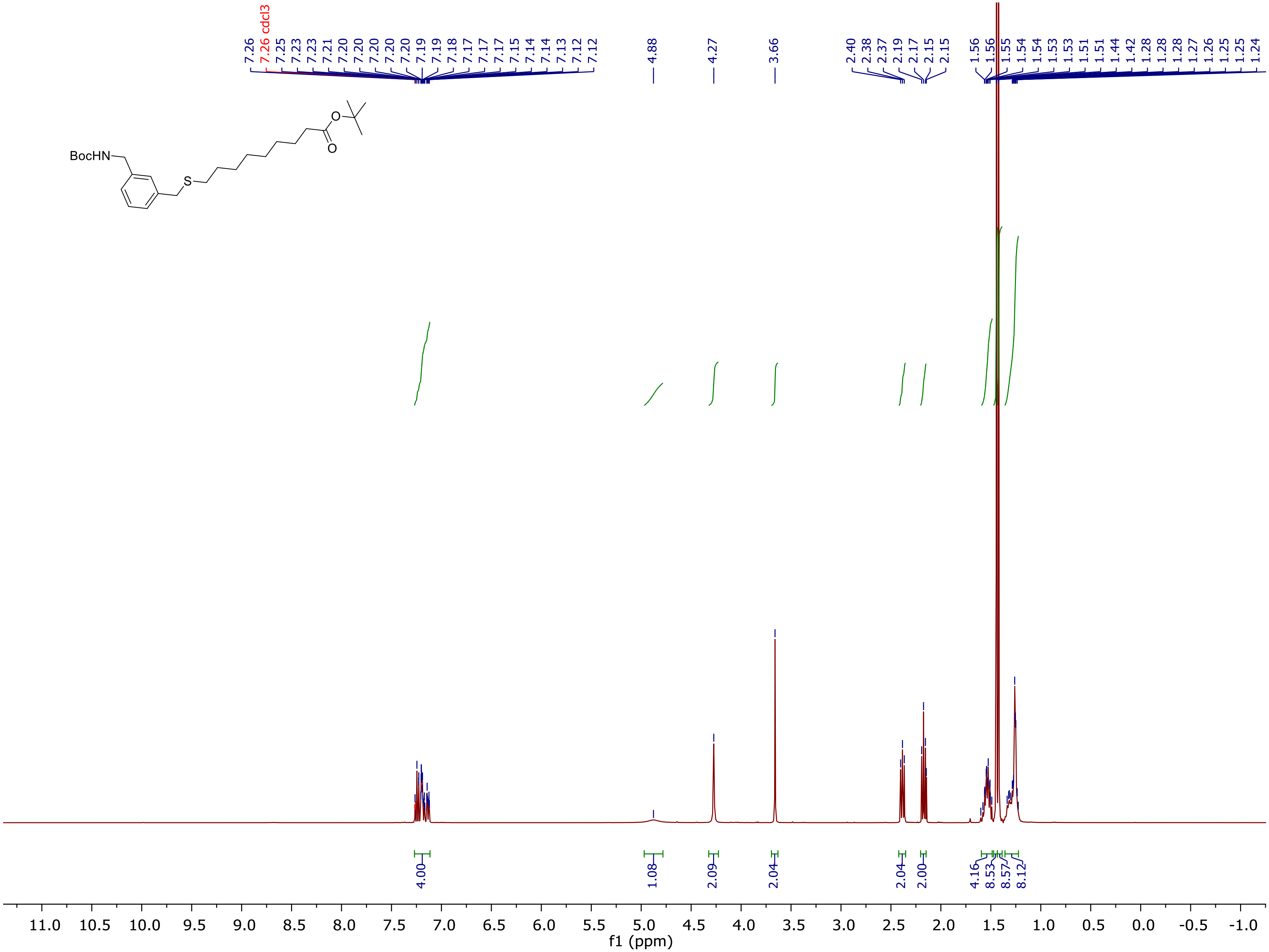

BocHN
S
O
O
7.26
7.26 cdcl3
7.25
7.23
7.23
7.21
7.20
7.20
7.20
7.20
7.20
7.19
7.19
7.18
7.17
7.17
7.17
7.15
7.14
7.14
7.13
7.12
7.12
4.88
4.27
3.66
2.40
2.38
2.37
2.19
2.17
2.15
2.15
1.56
1.56
1.55
1.54
1.54
1.53
1.53
1.51
1.51
1.44
1.42
1.28
1.28
1.28
1.27
1.26
1.25
1.25
1.24
4.00
1.08
2.09
2.04
2.04
2.00
4.16
8.53
8.57
8.12
11.0
10.5
10.0
9.5
9.0
8.5
8.0
7.5
7.0
6.5
6.0
5.5
5.0
4.5
4.0
3.5
3.0
2.5
2.0
1.5
1.0
0.5
0.0
-0.5
-1.0
f1 (ppm)

^{13}C NMR of **10:**

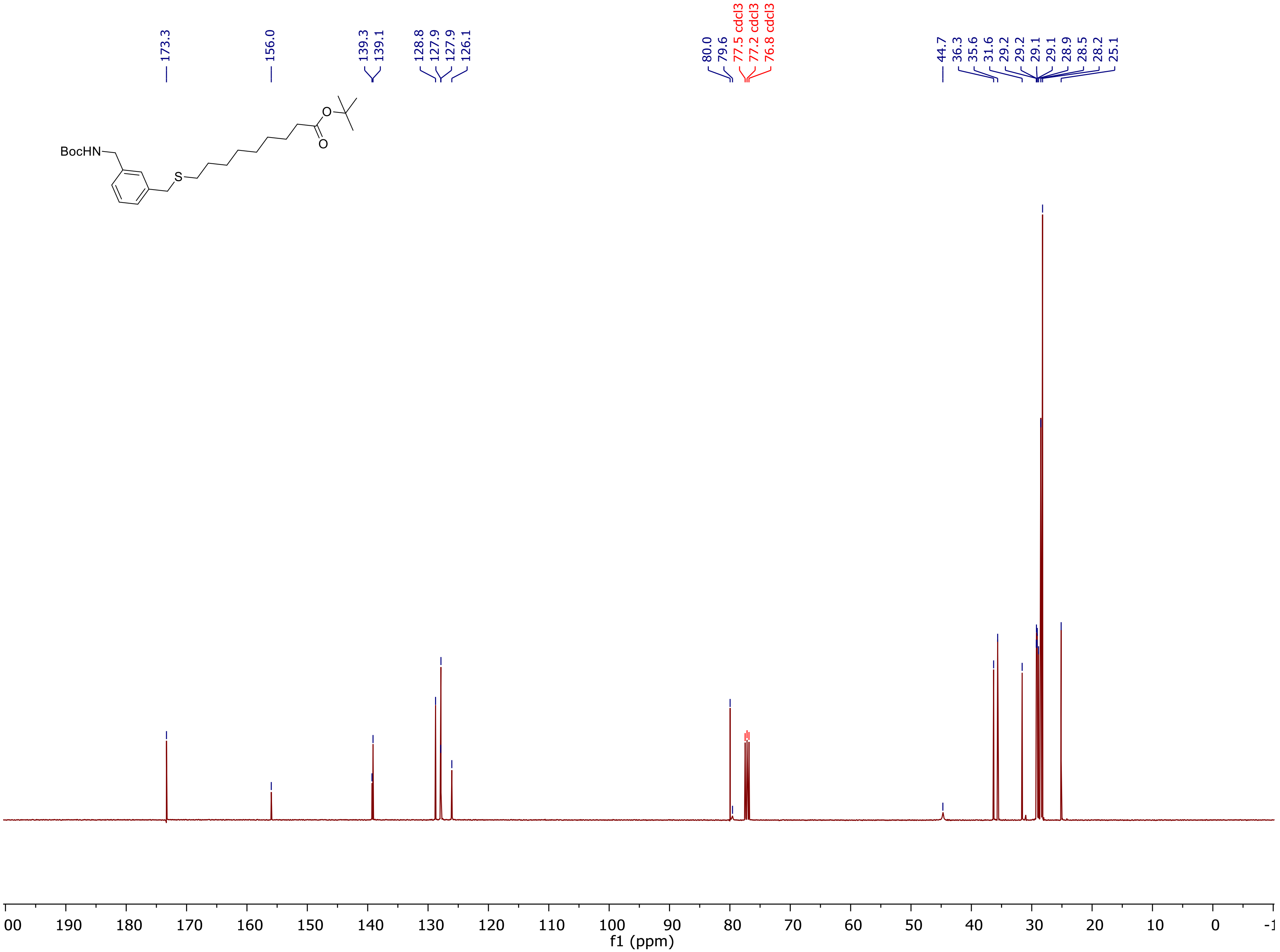
173.3
156.0
139.3
139.1
128.8
127.9
127.9
126.1
80.0
79.6
77.5 cdcl3
77.2 cdcl3
76.8 cdcl3
44.7
36.3
35.6
31.6
29.2
29.2
29.1
29.1
28.9
28.5
28.2
25.1
BocHN
S
O
O
00
190
180
170
160
150
140
130
120
110
100
90
80
70
60
50
40
30
20
10
0
-1
f1 (ppm)

^{1}H NMR of **11:**

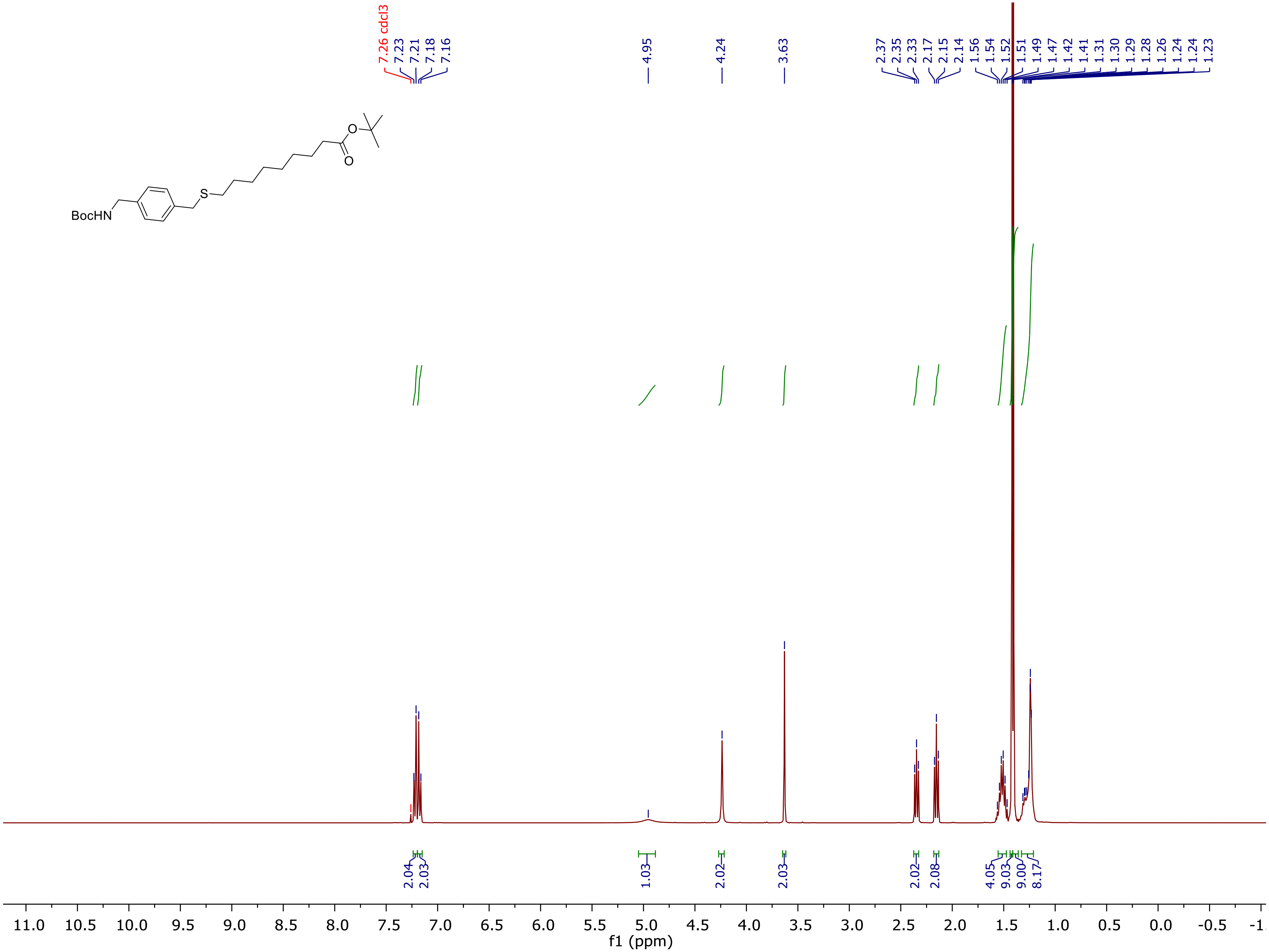

7.26 cdcl3
7.23
7.21
7.18
7.16
4.95
4.24
3.63
2.37
2.35
2.33
2.17
2.15
2.14
1.56
1.54
1.52
1.51
1.49
1.47
1.42
1.41
1.31
1.30
1.29
1.28
1.26
1.24
1.24
1.23
BocHN
S
O
O
2.04
2.03
1.03
2.02
2.03
2.02
2.08
4.05
9.03
9.00
8.17
11.0 10.5 10.0 9.5 9.0 8.5 8.0 7.5 7.0 6.5 6.0 5.5 5.0 4.5 4.0 3.5 3.0 2.5 2.0 1.5 1.0 0.5 0.0 -0.5 -1
f1 (ppm)

^{13}C NMR of **11:**

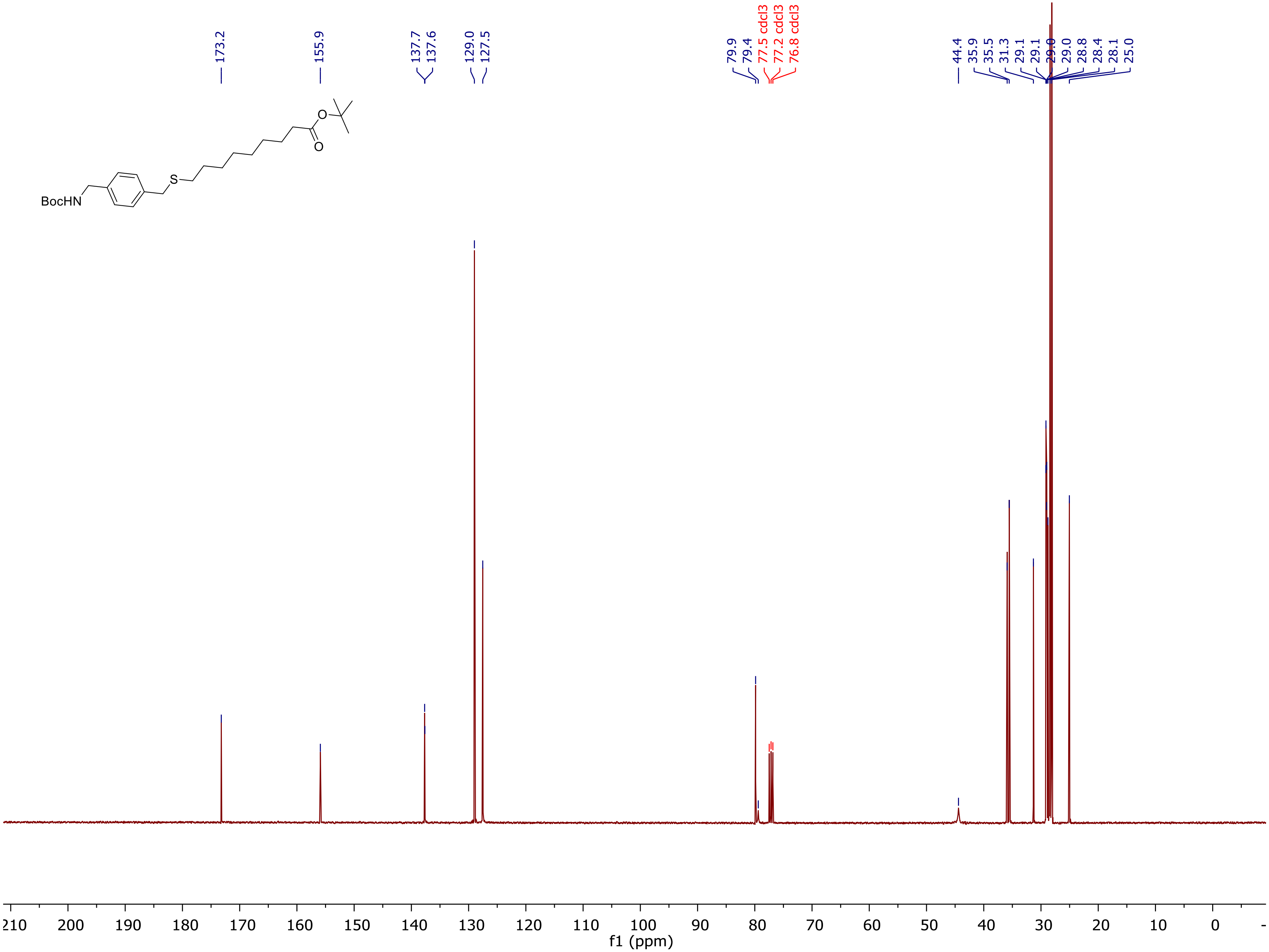
173.2
155.9
137.7
137.6
129.0
127.5
79.9
79.4
77.5 cdcl3
77.2 cdcl3
76.8 cdcl3
44.4
35.9
35.5
31.3
29.1
29.1
29.0
29.0
28.8
28.4
28.1
25.0
BocHN
S
O
O
210
200
190
180
170
160
150
140
130
120
110
100
90
80
70
60
50
40
30
20
10
0
f1 (ppm)

^{1}H NMR of **12:**

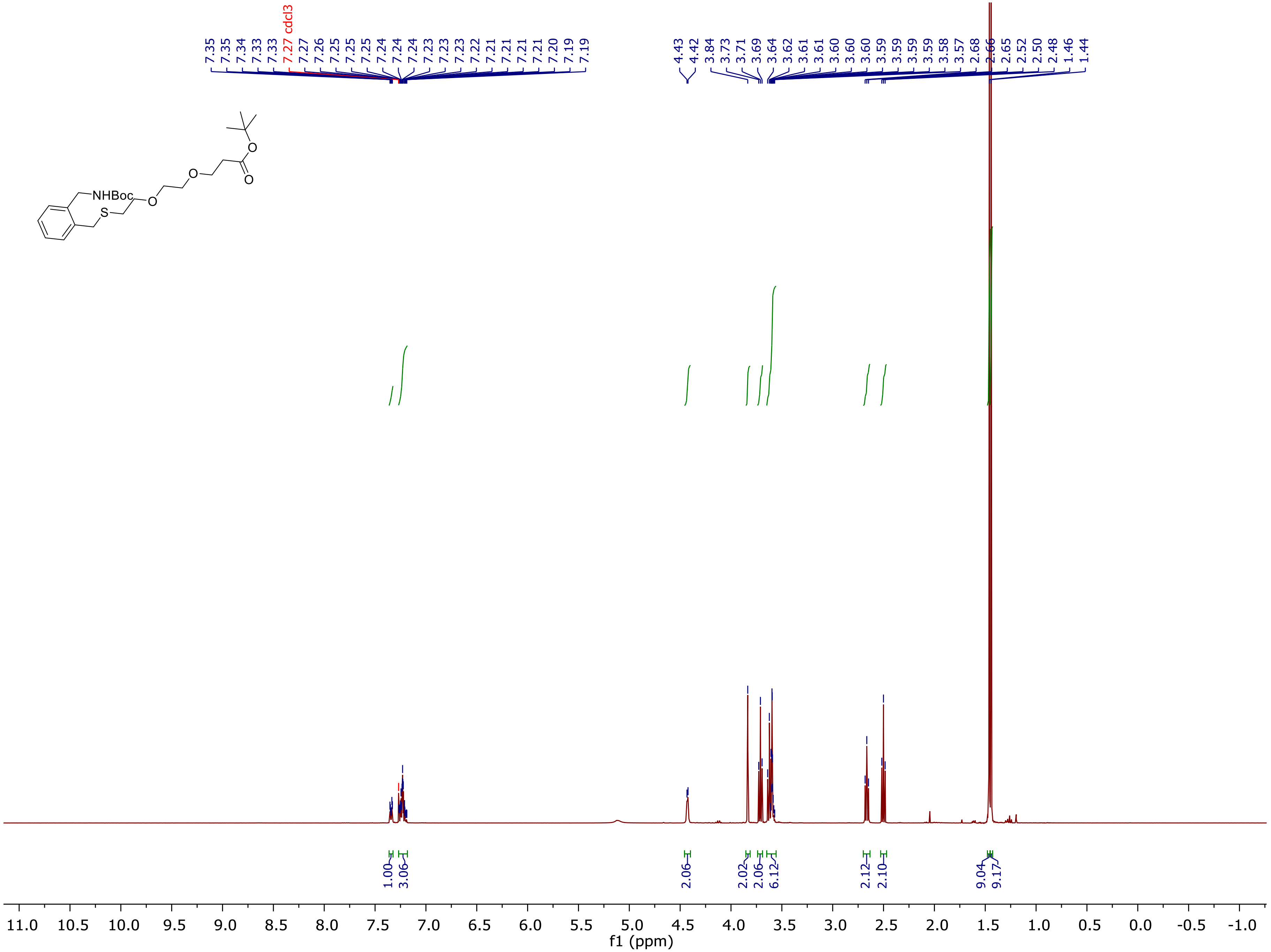
NHBoc
S
O
O
O
O
7.35
7.35
7.34
7.33
7.33
7.27 cdcl3
7.27
7.26
7.25
7.25
7.25
7.24
7.24
7.24
7.23
7.23
7.23
7.22
7.21
7.21
7.21
7.21
7.20
7.19
7.19
4.43
4.42
3.84
3.73
3.71
3.69
3.64
3.62
3.61
3.61
3.60
3.60
3.60
3.59
3.59
3.59
3.58
3.57
2.68
2.66
2.65
2.52
2.50
2.48
1.46
1.44
1.00
3.06
2.06
2.02
2.06
6.12
2.12
2.10
9.04
9.17
11.0
10.5
10.0
9.5
9.0
8.5
8.0
7.5
7.0
6.5
6.0
5.5
5.0
4.5
4.0
3.5
3.0
2.5
2.0
1.5
1.0
0.5
0.0
-0.5
-1.0
f1 (ppm)

^{13}C NMR of **12:**

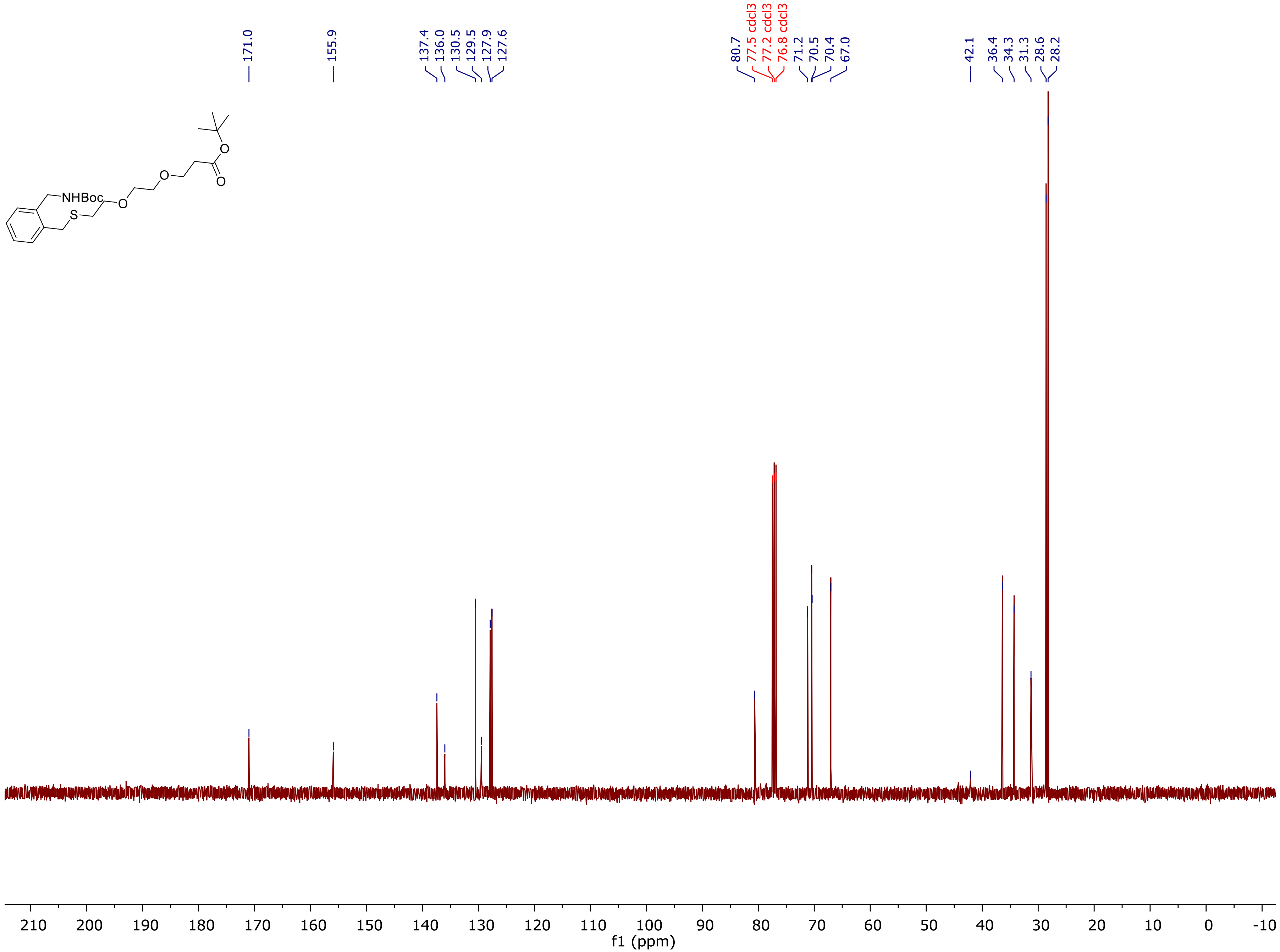
171.0
155.9
137.4
136.0
130.5
129.5
127.9
127.6
80.7
77.5 cdcl3
77.2 cdcl3
76.8 cdcl3
71.2
70.5
70.4
67.0
42.1
36.4
34.3
31.3
28.6
28.2
NHBoc
S
O
O
O
O
210
200
190
180
170
160
150
140
130
120
110
100
90
80
70
60
50
40
30
20
10
0
-10
f1 (ppm)

^{1}H NMR of **13:**

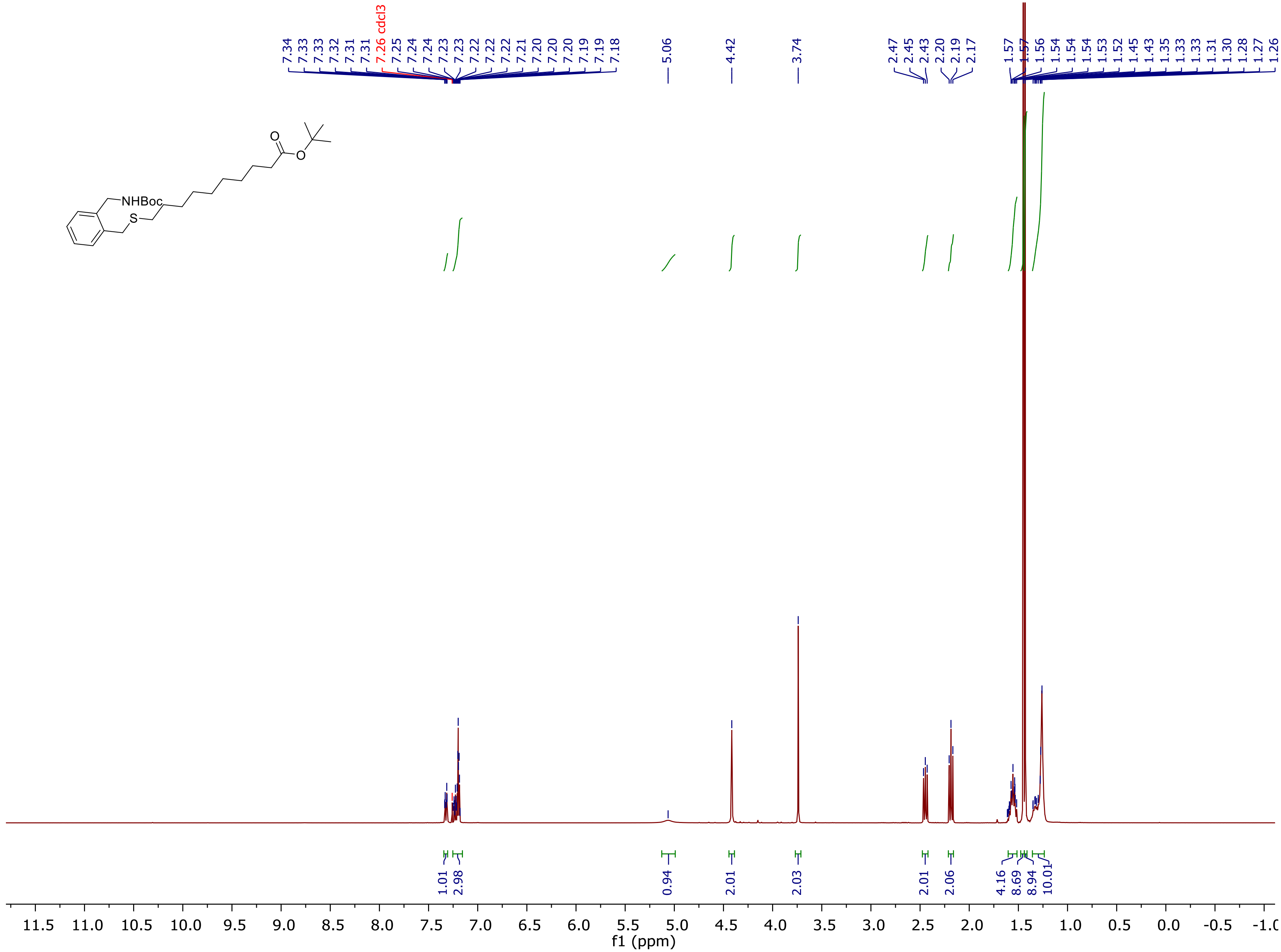

7.34 7.33 7.33 7.32 7.31 7.31 7.26 cdcl3 7.25 7.24 7.24 7.23 7.23 7.22 7.22 7.22 7.21 7.20 7.20 7.20 7.19 7.19 7.18
5.06
4.42
3.74
2.47 2.45 2.43 2.20 2.19 2.17
1.57 1.57 1.56 1.54 1.54 1.54 1.53 1.52 1.45 1.43 1.35 1.33 1.33 1.31 1.30 1.28 1.27 1.26
NHBoc
S
O
O
1.01 2.98 0.94 2.01 2.03 2.01 2.06 4.16 8.69 8.94 10.01
11.5 11.0 10.5 10.0 9.5 9.0 8.5 8.0 7.5 7.0 6.5 6.0 5.5 5.0 4.5 4.0 3.5 3.0 2.5 2.0 1.5 1.0 0.5 0.0 -0.5
f1 (ppm)

^{13}C NMR of **13:**

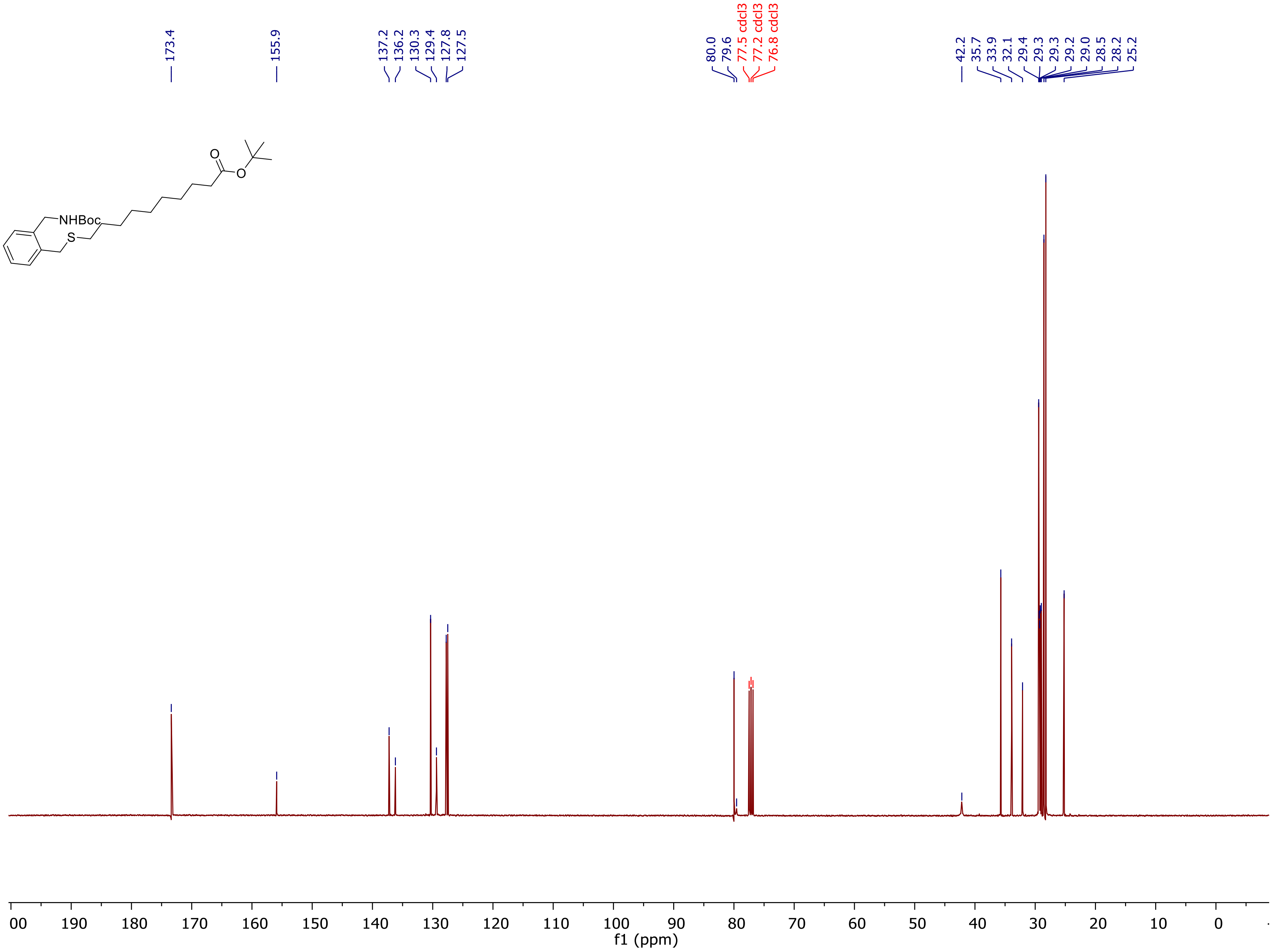
173.4
155.9
137.2
136.2
130.3
129.4
127.8
127.5
80.0
79.6
77.5 cdcl3
77.2 cdcl3
76.8 cdcl3
42.2
35.7
33.9
32.1
29.4
29.3
29.3
29.2
29.0
28.5
28.2
25.2
NHBoc
S
O
O
f1 (ppm)

^{1}H NMR of **14:**

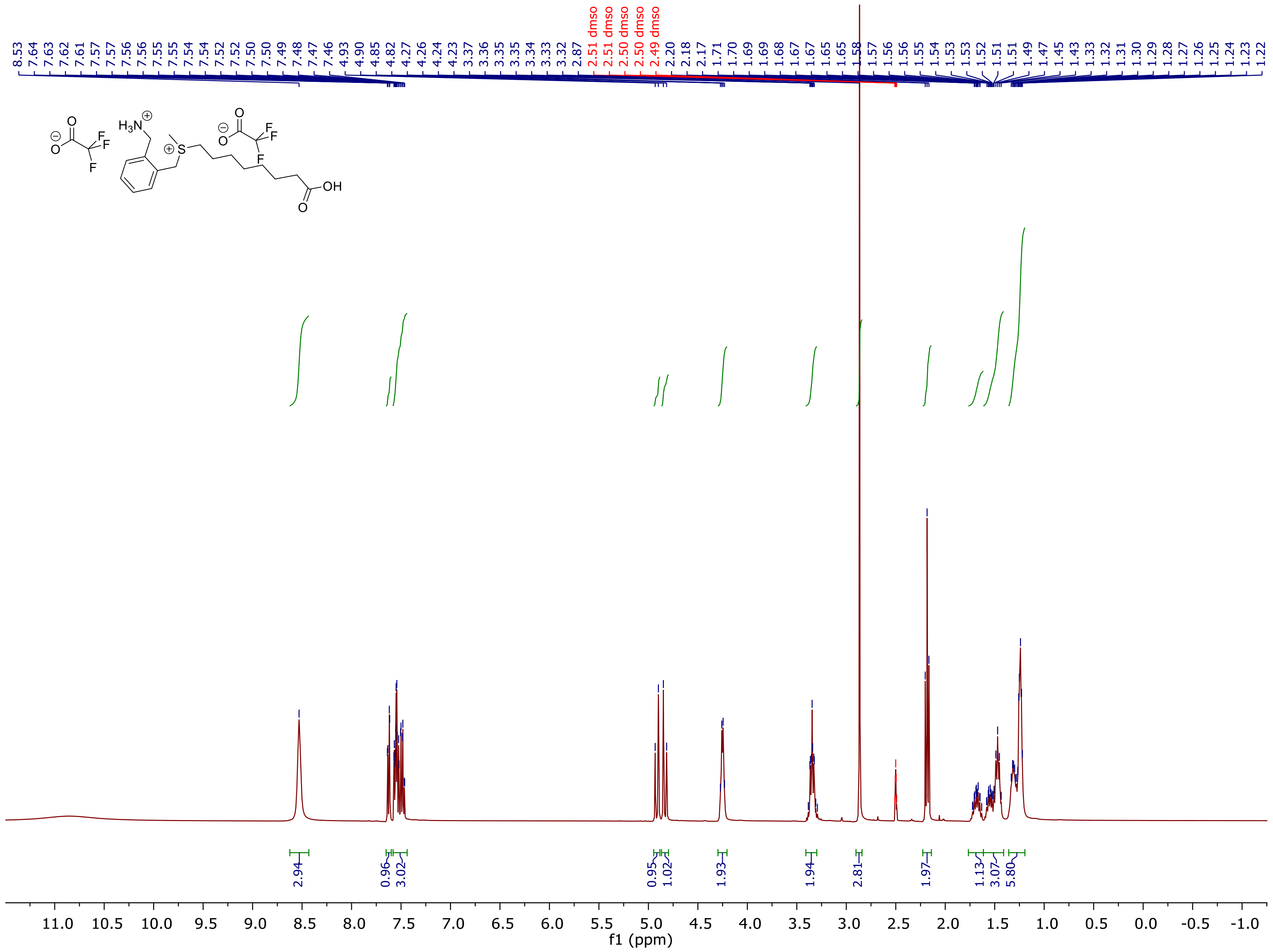

^{13}C NMR of **14:**

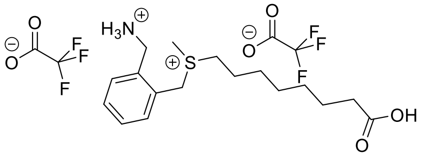

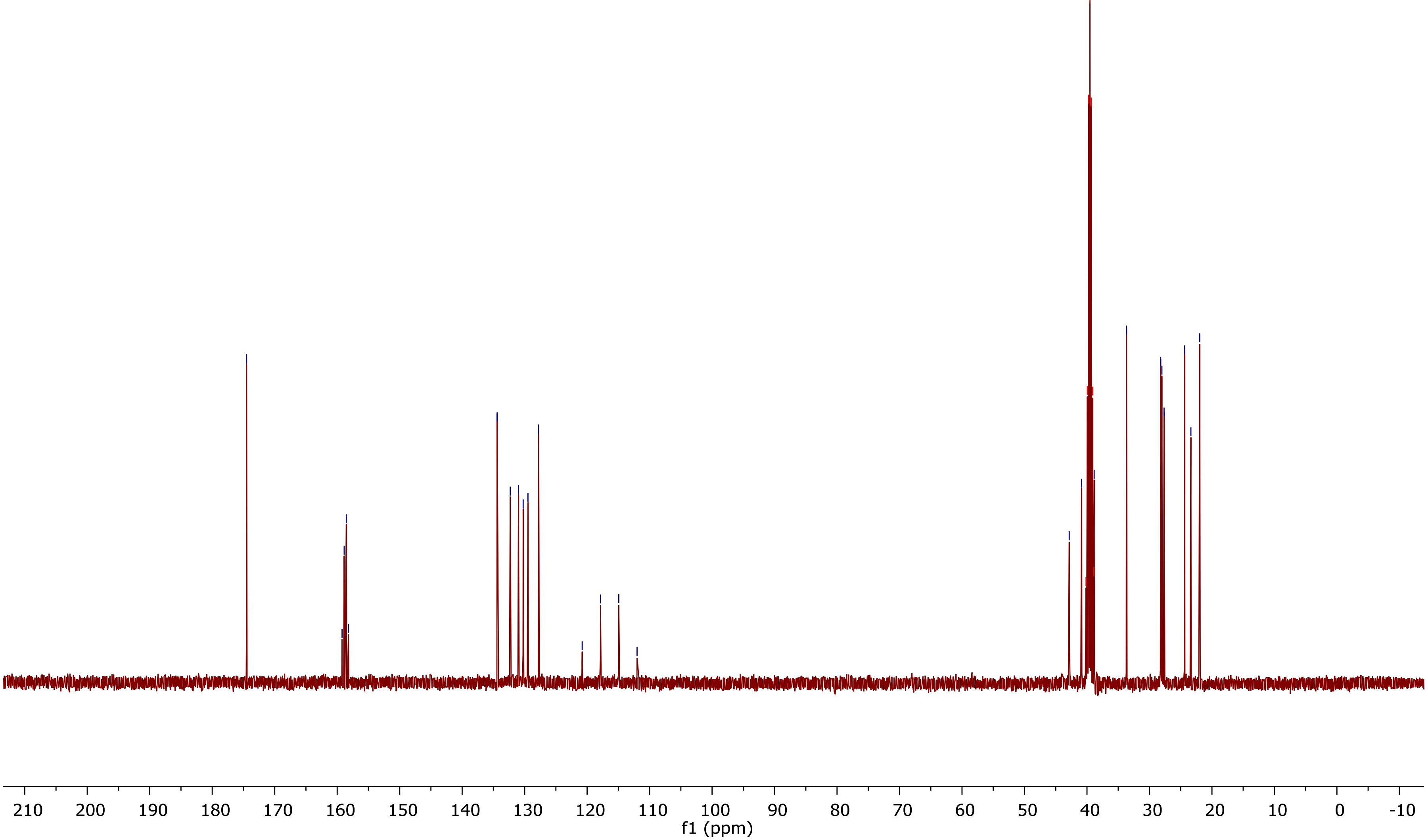
174.5
159.2
158.9
158.5
158.2
134.4
132.3
131.0
130.2
129.5
127.8
120.8
117.9
114.9
112.0
42.8
40.9
40.1 dmso
39.9 dmso
39.7 dmso
39.5 dmso
39.3 dmso
39.1 dmso
38.9 dmso
38.8
33.7
28.2
28.0
27.7
24.4
23.4
22.0
f1 (ppm)

^{1}H NMR of **15:**

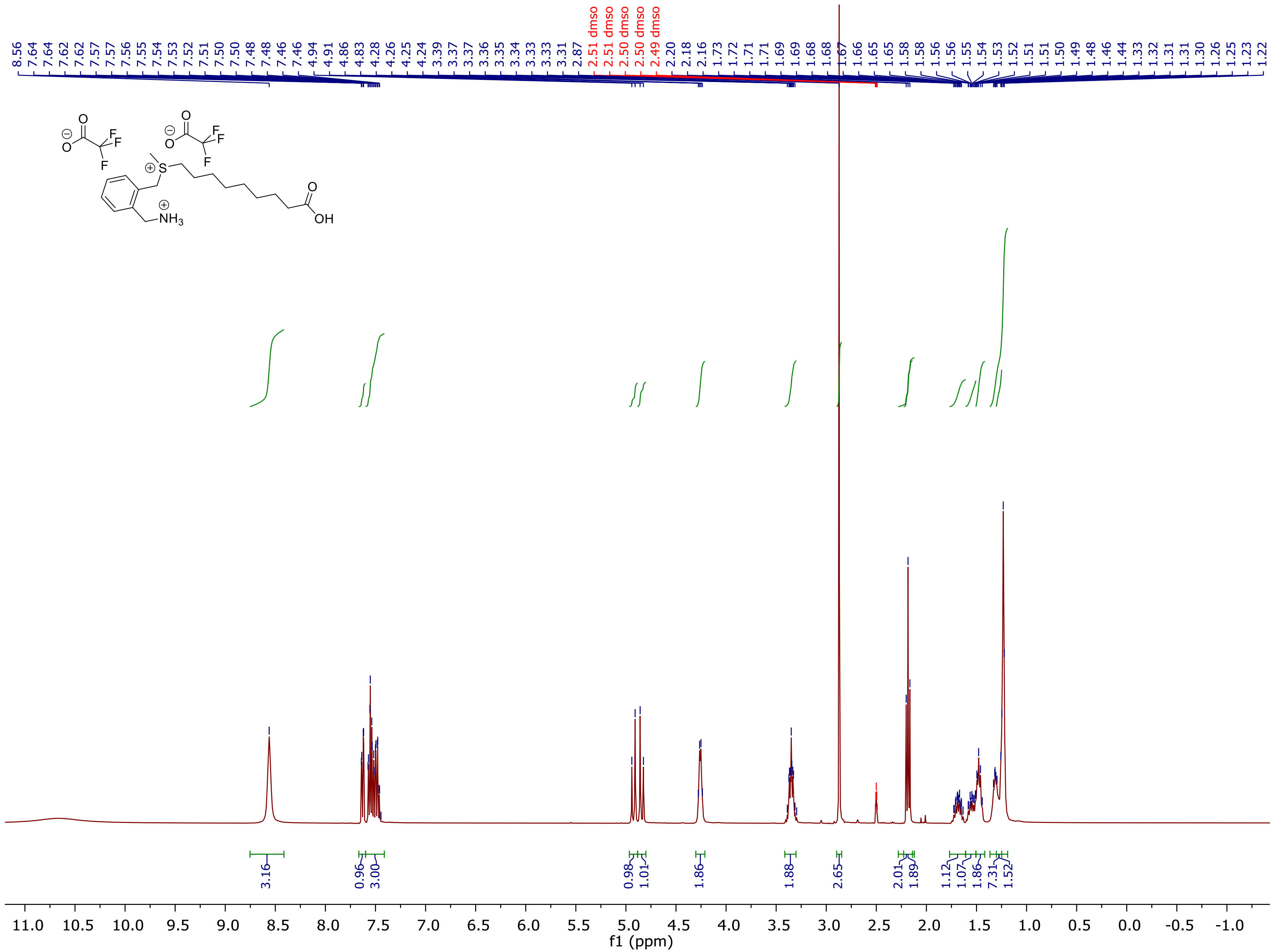

^{13}C NMR of **15:**

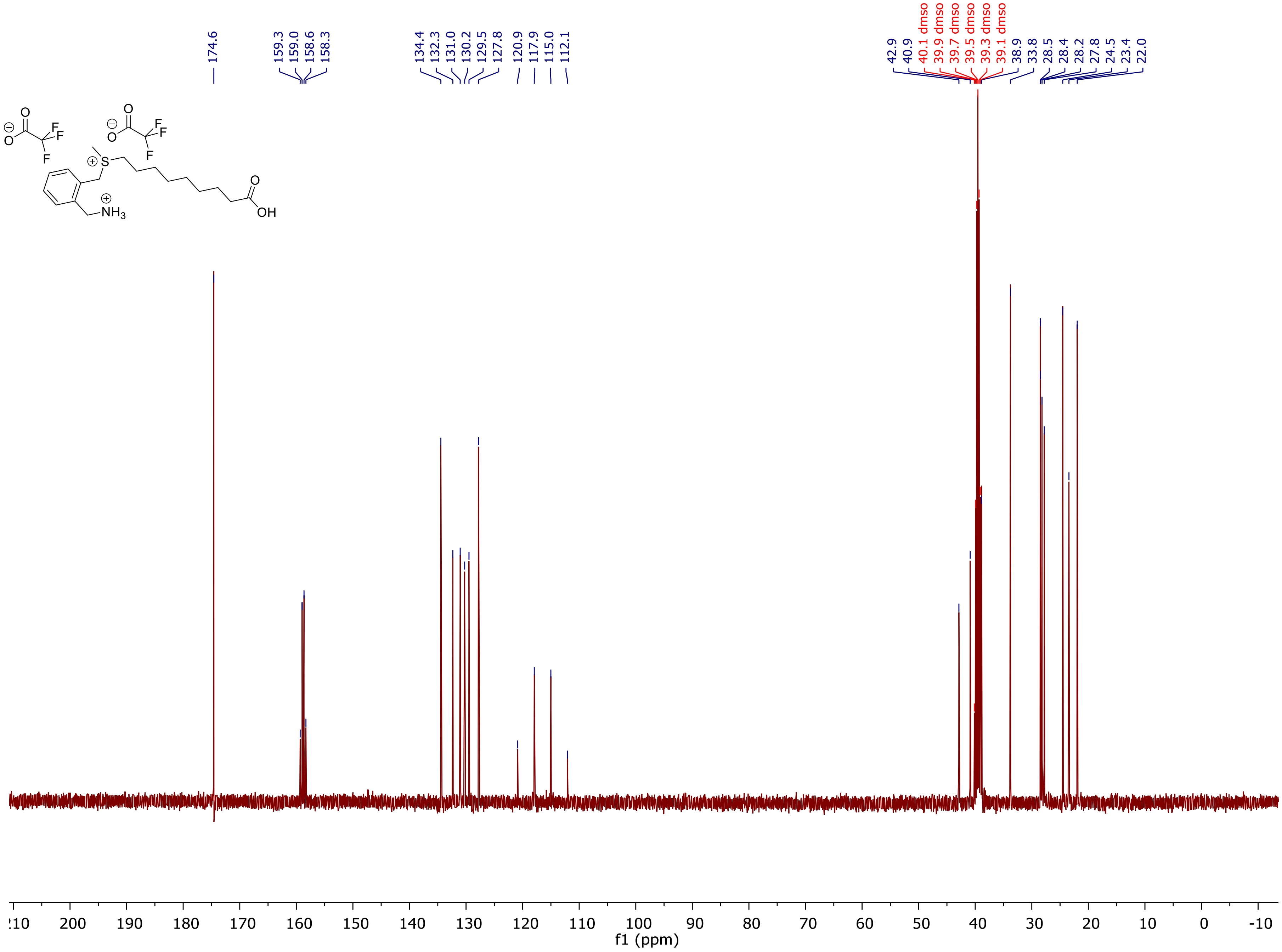
174.6
159.3
159.0
158.6
158.3
134.4
132.3
131.0
130.2
129.5
127.8
120.9
117.9
115.0
112.1
42.9
40.9
40.1 dmso
39.9 dmso
39.7 dmso
39.5 dmso
39.3 dmso
39.1 dmso
38.9
33.8
28.5
28.4
28.2
27.8
24.5
23.4
22.0
O
F
F
F
S
NH3
OH
f1 (ppm)

^{1}H NMR of **16:**

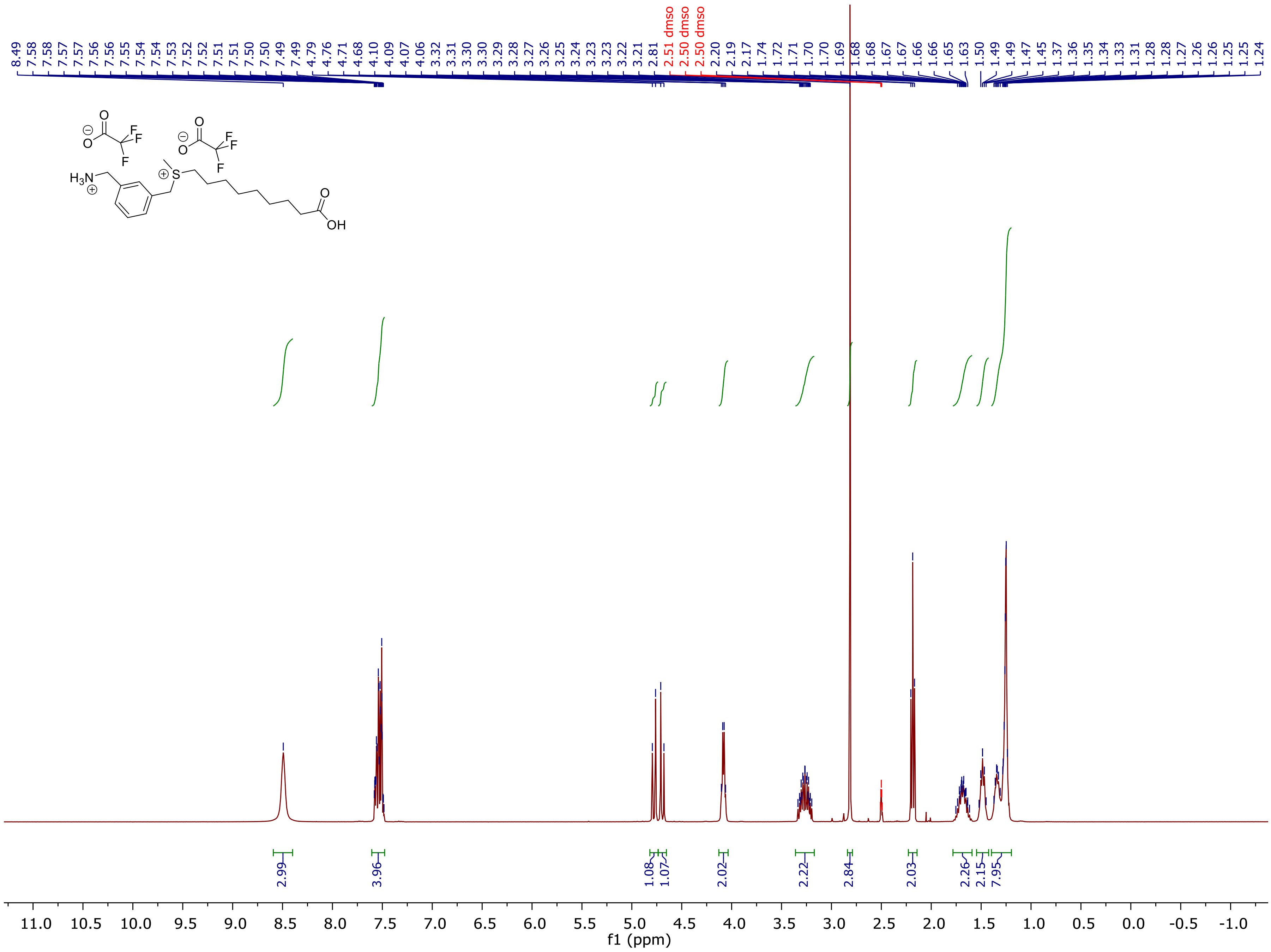
8.49 7.58 7.58 7.57 7.57 7.56 7.56 7.55 7.54 7.54 7.53 7.52 7.52 7.51 7.51 7.50 7.50 7.49 7.49 4.79 4.76 4.71 4.68 4.10 4.09 4.07 4.06 3.32 3.31 3.30 3.30 3.29 3.28 3.27 3.26 3.25 3.24 3.23 3.23 3.22 3.21 2.81 2.51 dmso 2.50 dmso 2.50 dmso 2.20 2.19 2.17 1.74 1.72 1.71 1.70 1.70 1.69 1.68 1.68 1.67 1.67 1.66 1.66 1.65 1.63 1.50 1.49 1.49 1.47 1.45 1.37 1.36 1.35 1.34 1.33 1.31 1.28 1.27 1.26 1.26 1.25 1.24
2.99
3.96
1.08
1.07
2.02
2.22
2.84
2.03
2.26
2.15
7.95
11.0 10.5 10.0 9.5 9.0 8.5 8.0 7.5 7.0 6.5 6.0 5.5 5.0 4.5 4.0 3.5 3.0 2.5 2.0 1.5 1.0 0.5 0.0 -0.5 -1.0
f1 (ppm)

^{13}C NMR of **16:**

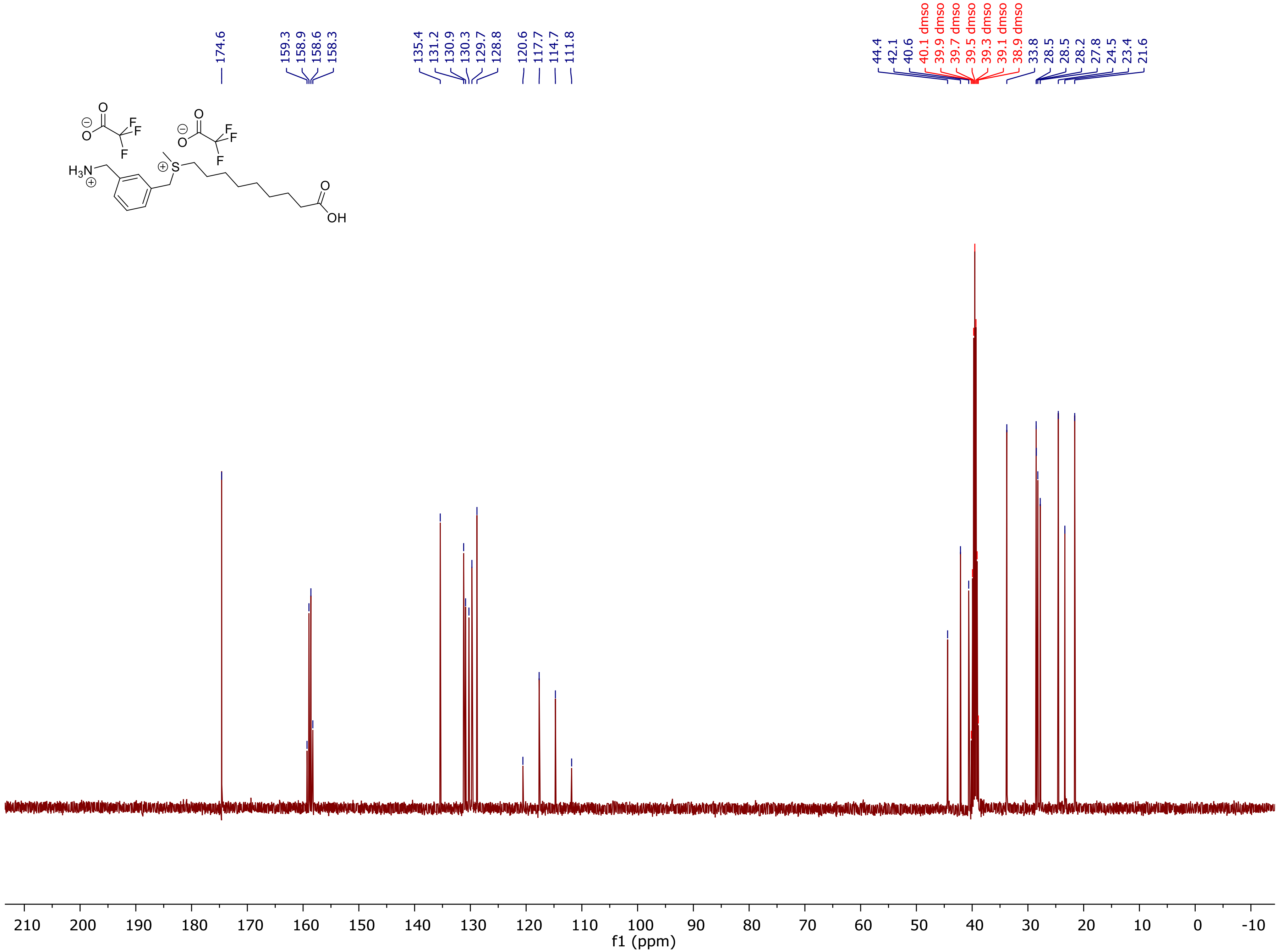

174.6
159.3
158.9
158.6
158.3
135.4
131.2
130.9
130.3
129.7
128.8
120.6
117.7
114.7
111.8
44.4
42.1
40.6
40.1 dmso
39.9 dmso
39.7 dmso
39.5 dmso
39.3 dmso
39.1 dmso
38.9 dmso
33.8
28.5
28.5
28.2
27.8
24.5
23.4
21.6
f1 (ppm)

^{1}H NMR of **17:**

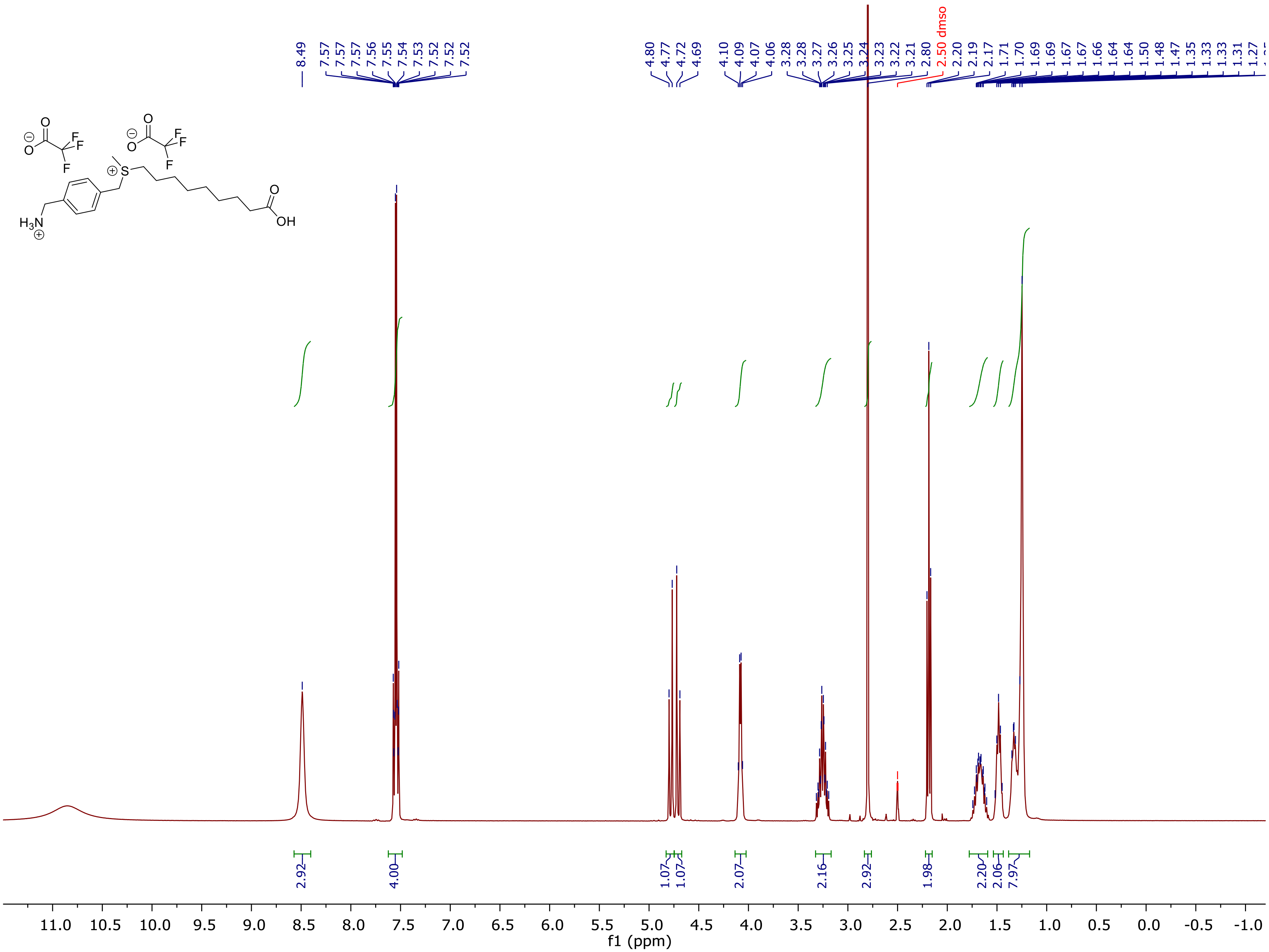

8.49
7.57
7.57
7.57
7.56
7.55
7.54
7.53
7.52
7.52
7.52
4.80
4.77
4.72
4.69
4.10
4.09
4.07
4.06
3.28
3.28
3.27
3.26
3.25
3.24
3.23
3.22
3.21
2.80
2.50 dmso
2.20
2.19
2.17
1.71
1.70
1.69
1.69
1.67
1.67
1.66
1.64
1.50
1.48
1.47
1.35
1.33
1.33
1.31
1.27
O
F
F
F
S
H3N
OH
2.92
4.00
1.07
1.07
2.07
2.16
2.92
1.98
2.20
2.06
7.97
11.0
10.5
10.0
9.5
9.0
8.5
8.0
7.5
7.0
6.5
6.0
5.5
5.0
4.5
4.0
3.5
3.0
2.5
2.0
1.5
1.0
0.5
0.0
-0.5
-1.0
f1 (ppm)

^{13}C NMR of **17:**

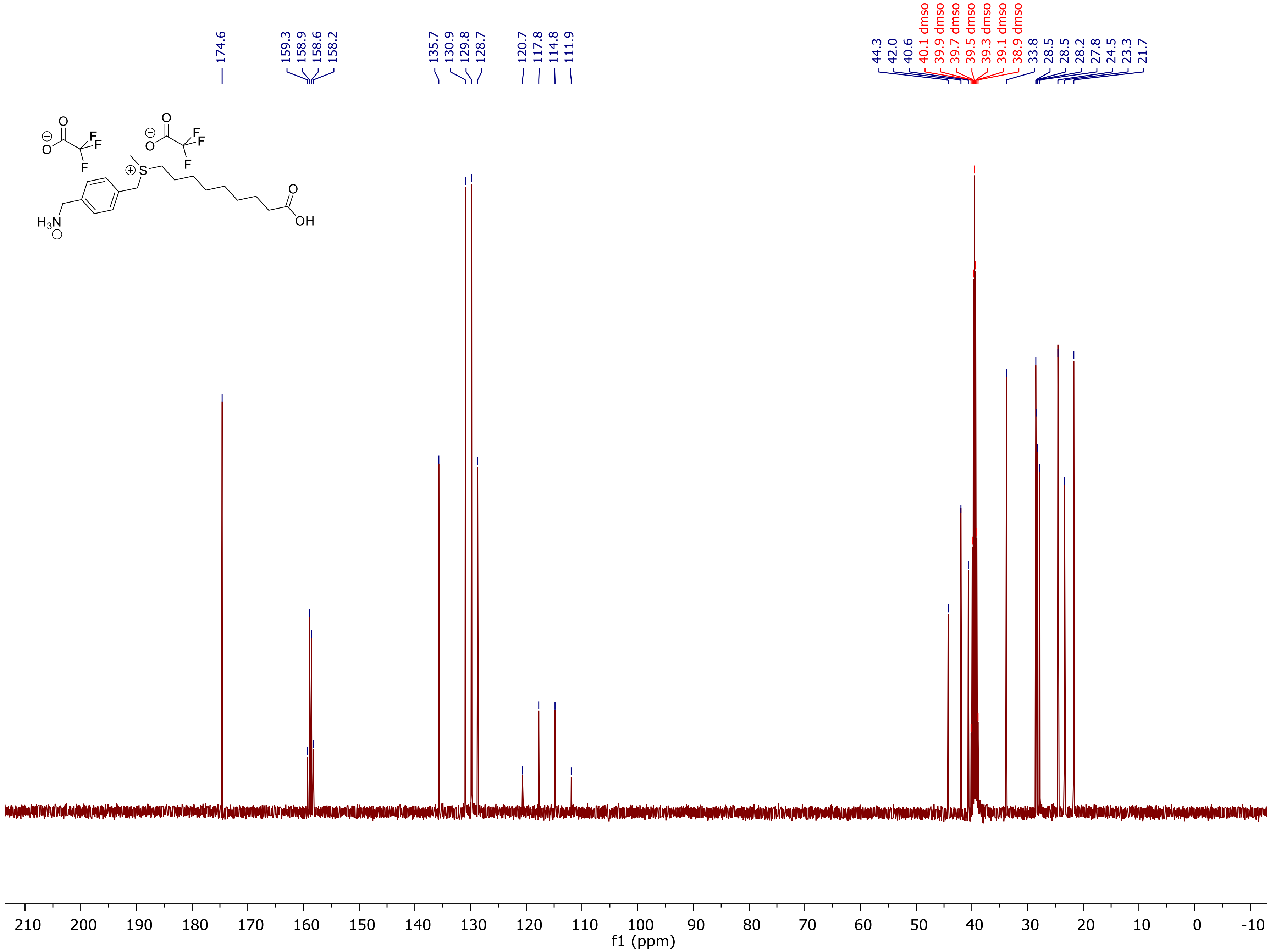

174.6
159.3
158.9
158.6
158.2
135.7
130.9
129.8
128.7
120.7
117.8
114.8
111.9
44.3
42.0
40.6
40.1 dmso
39.9 dmso
39.7 dmso
39.5 dmso
39.3 dmso
39.1 dmso
38.9 dmso
33.8
28.5
28.5
28.2
27.8
24.5
23.3
21.7
O
F
F
F
O
F
F
F
S
O
OH
H_3N
210
200
190
180
170
160
150
140
130
120
110
100
90
80
70
60
50
40
30
20
10
0
-10
f1 (ppm)

^{1}H NMR of **18:**

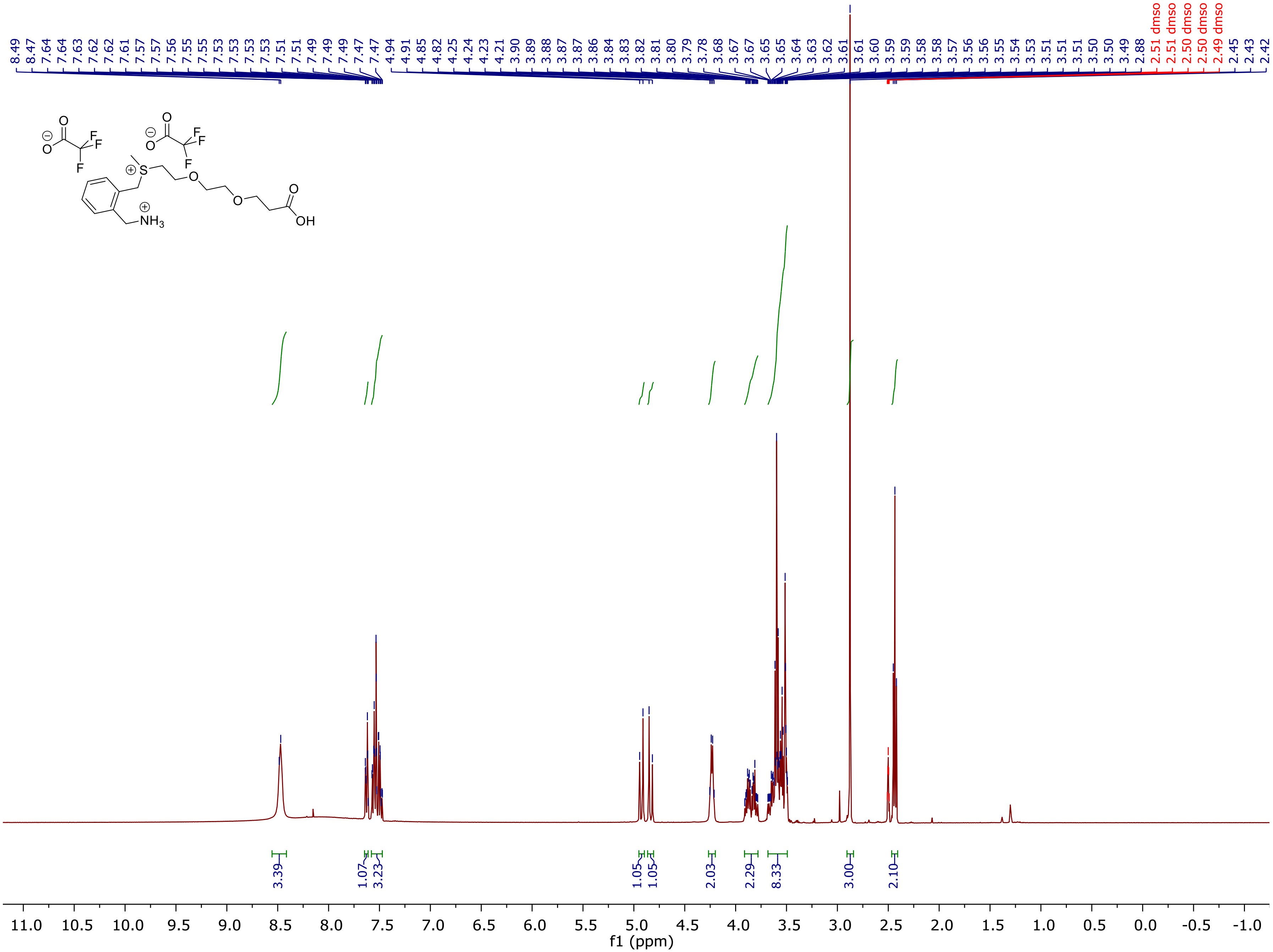

8.49 8.47 7.64 7.64 7.63 7.62 7.62 7.61 7.57 7.57 7.56 7.55 7.55 7.53 7.53 7.53 7.53 7.51 7.51 7.49 7.49 7.47 7.47 4.94 4.91 4.85 4.82 4.25 4.24 4.23 4.21 3.90 3.89 3.88 3.87 3.87 3.86 3.84 3.83 3.82 3.81 3.80 3.79 3.78 3.68 3.67 3.67 3.65 3.65 3.64 3.63 3.62 3.61 3.61 3.60 3.59 3.59 3.58 3.58 3.57 3.56 3.56 3.55 3.54 3.53 3.51 3.51 3.51 3.50 3.49 2.88
2.51 dmso
2.51 dmso
2.50 dmso
2.50 dmso
2.49 dmso
2.45 2.43 2.42
O
F
F
F
O
F
F
F
S
NH3
O
O
O
OH
3.39
1.07
3.23
1.05
1.05
2.03
2.29
8.33
3.00
2.10
11.0 10.5 10.0 9.5 9.0 8.5 8.0 7.5 7.0 6.5 6.0 5.5 5.0 4.5 4.0 3.5 3.0 2.5 2.0 1.5 1.0 0.5 0.0 -0.5 -1.0
f1 (ppm)

^{13}C NMR of **18:**

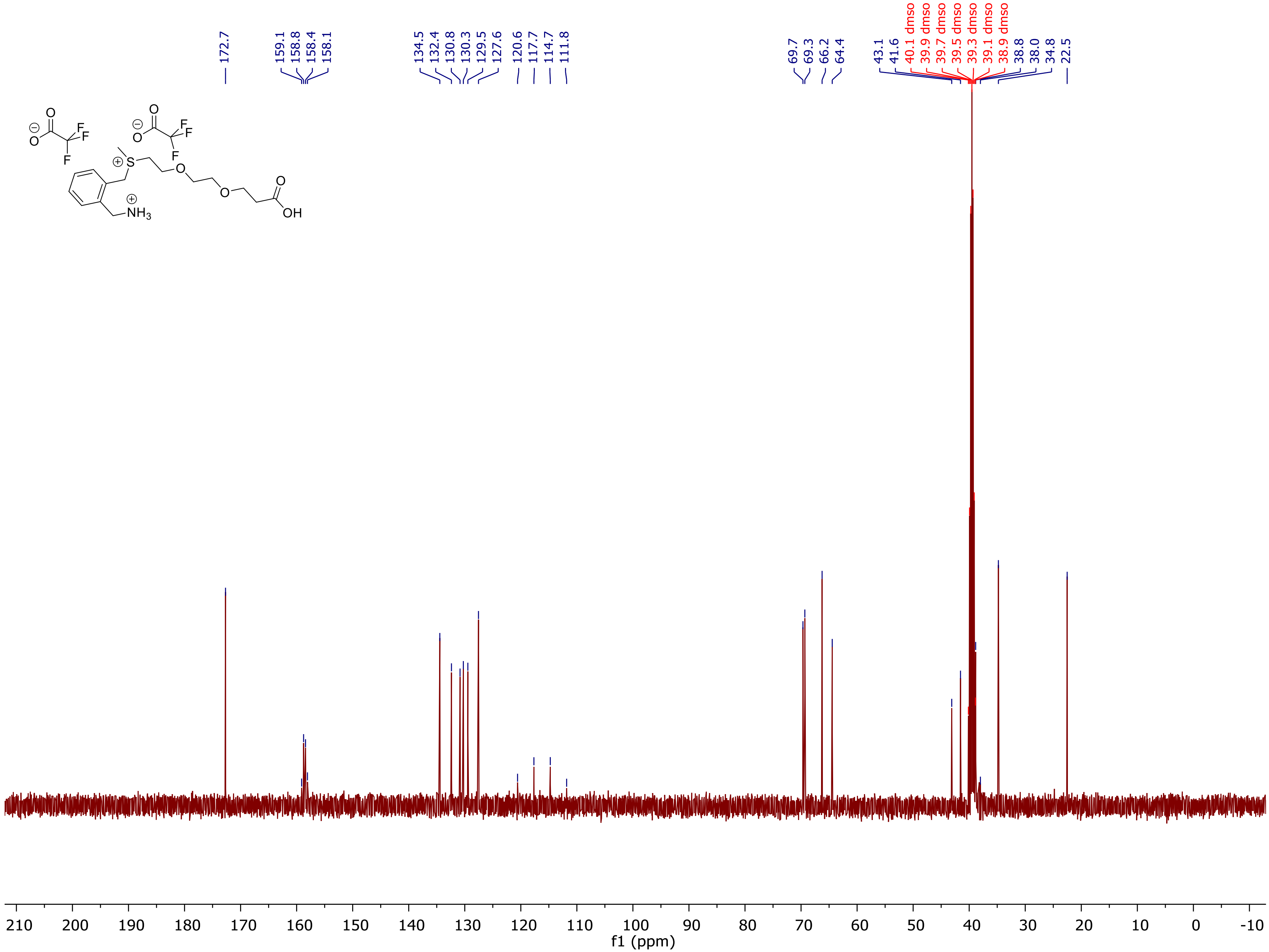
172.7
159.1
158.8
158.4
158.1
134.5
132.4
130.8
130.3
129.5
127.6
120.6
117.7
114.7
111.8
69.7
69.3
66.2
64.4
43.1
41.6
40.1 dmso
39.9 dmso
39.7 dmso
39.5 dmso
39.3 dmso
39.1 dmso
38.9 dmso
38.8
38.0
34.8
22.5
f1 (ppm)

^{1}H NMR of **19:**

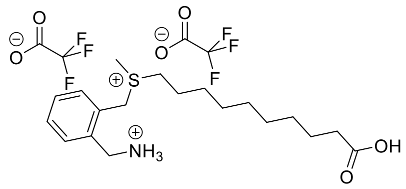
O
F
F
F
O
O
F
F
F
O
S
NH3
OH
O

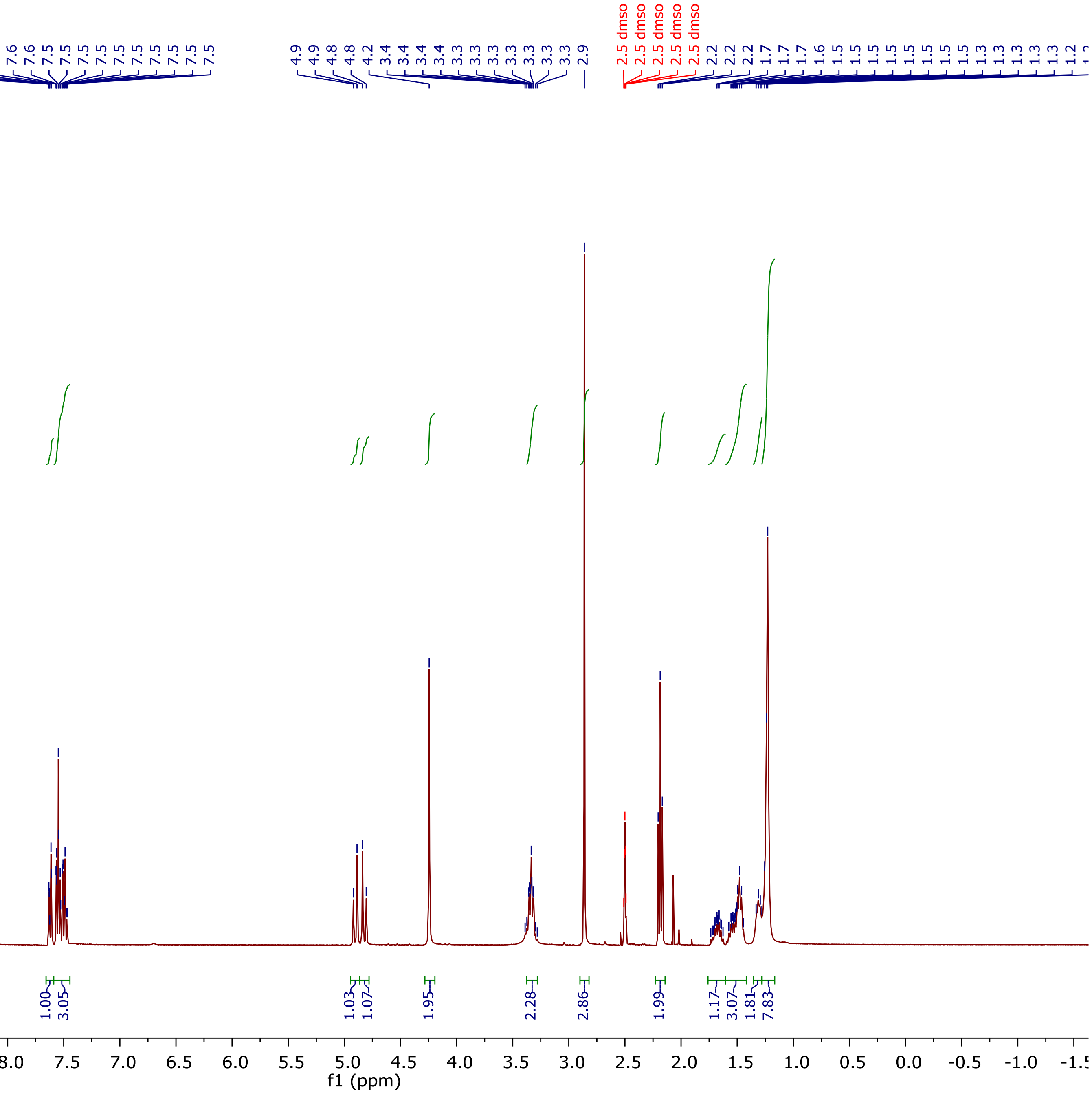
2.5 dmso
1.00
3.05
1.03
1.07
1.95
2.28
2.86
1.99
1.17
3.07
1.81
7.83
11.0
10.5
10.0
9.5
9.0
8.5
8.0
7.5
7.0
6.5
6.0
5.5
5.0
4.5
4.0
3.5
3.0
2.5
2.0
1.5
1.0
0.5
0.0
-0.5
-1.0
f1 (ppm)

^{13}C NMR of **19:**

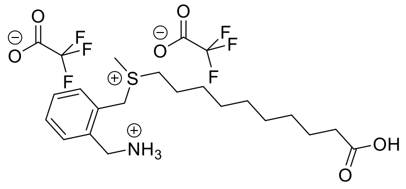
O
O
F
F
F
S
NH3
O
O
F
F
F
OH
O

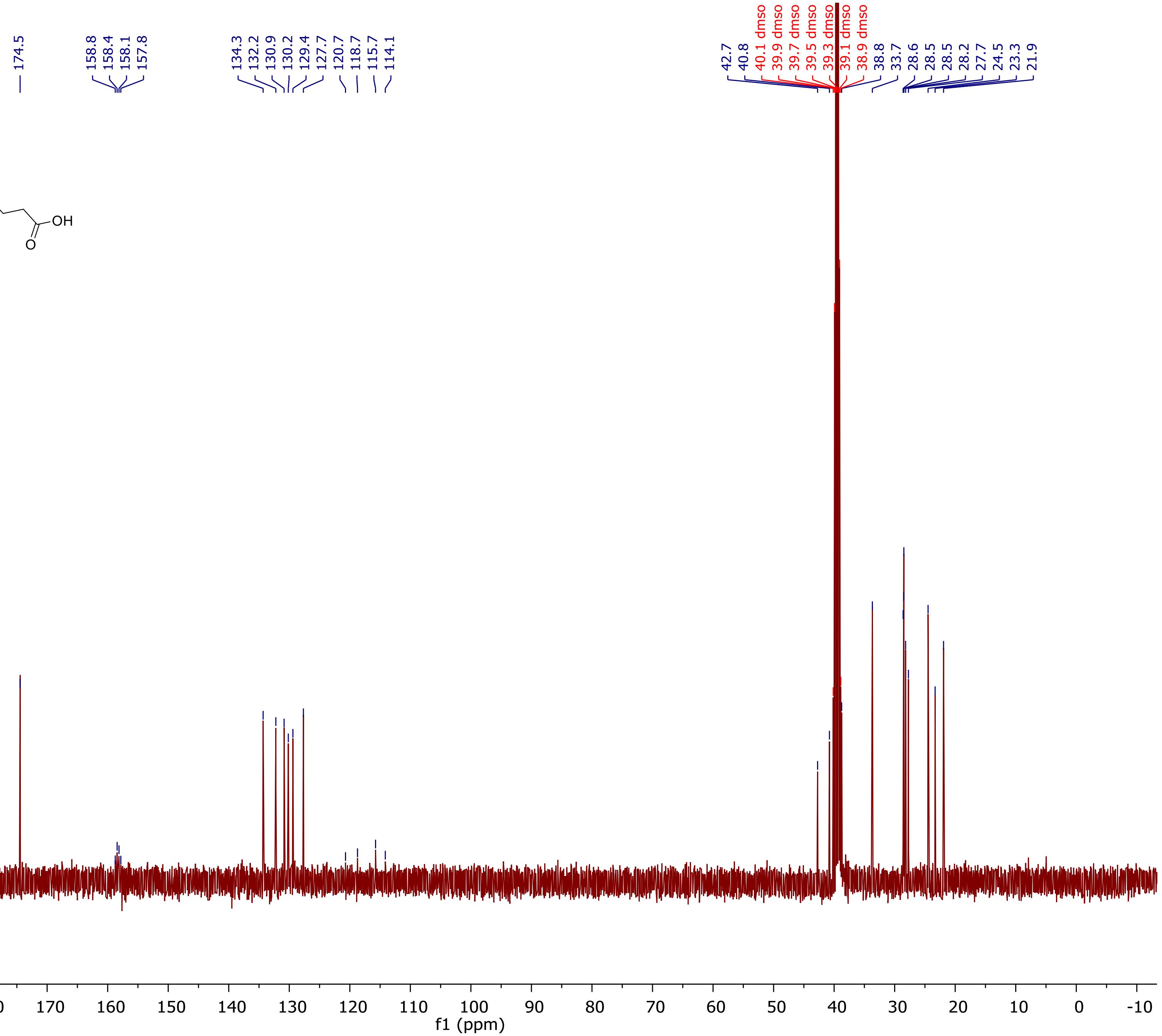
174.5
158.8
158.4
158.1
157.8
134.3
132.2
130.9
130.2
129.4
127.7
120.7
118.7
115.7
114.1
42.7
40.8
40.1 dmso
39.9 dmso
39.7 dmso
39.5 dmso
39.3 dmso
39.1 dmso
38.9 dmso
38.8
33.7
28.6
28.5
28.5
28.2
27.7
24.5
23.3
21.9
210
200
190
180
170
160
150
140
130
120
110
100
90
80
70
60
50
40
30
20
10
0
-10
f1 (ppm)

^{1}H NMR of ***CTX-NH₃-CF₃CO₂:***

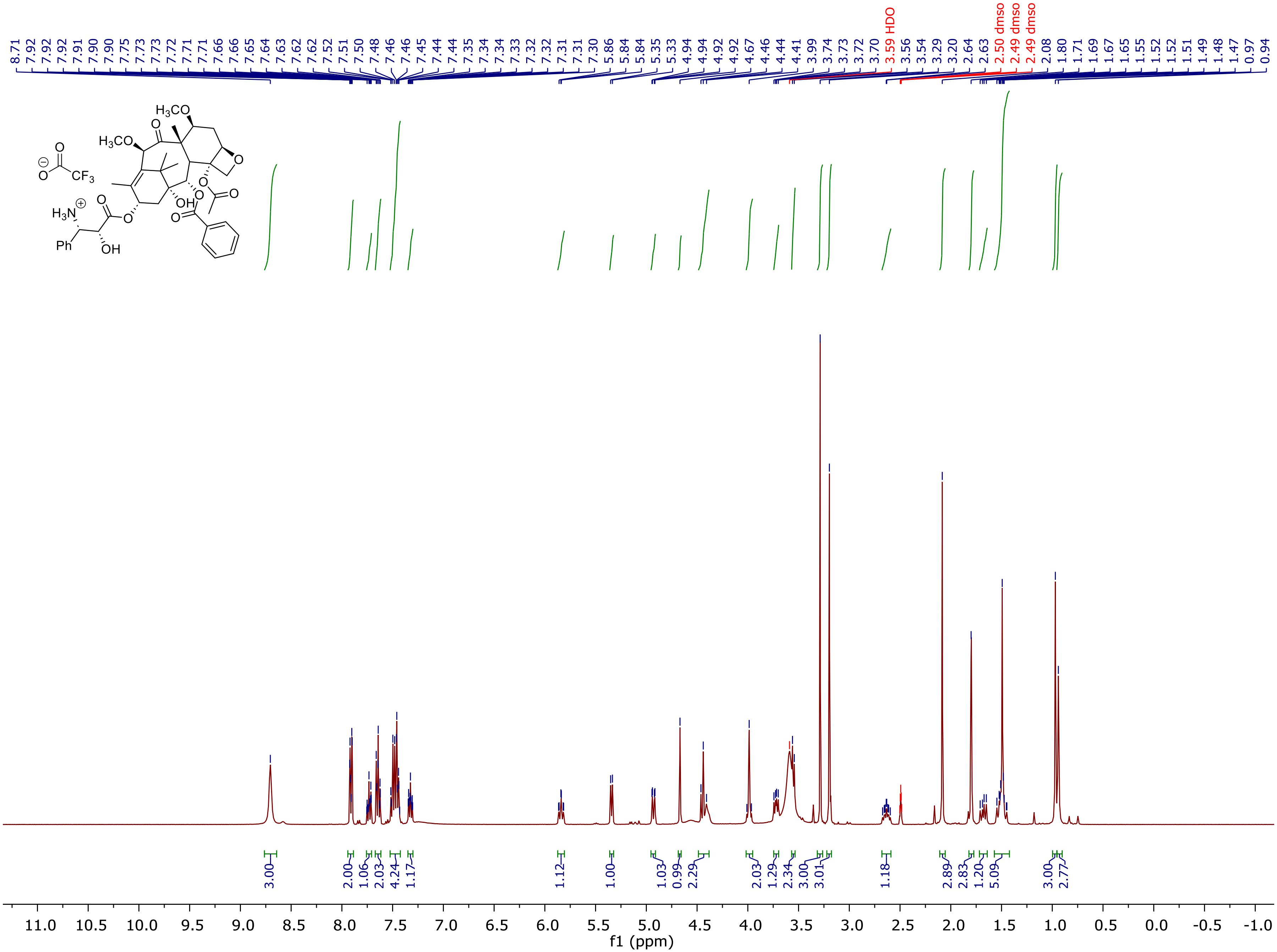

^{13}C NMR of ***CTX-NH$_3$-CF$_3$CO$_2$:***

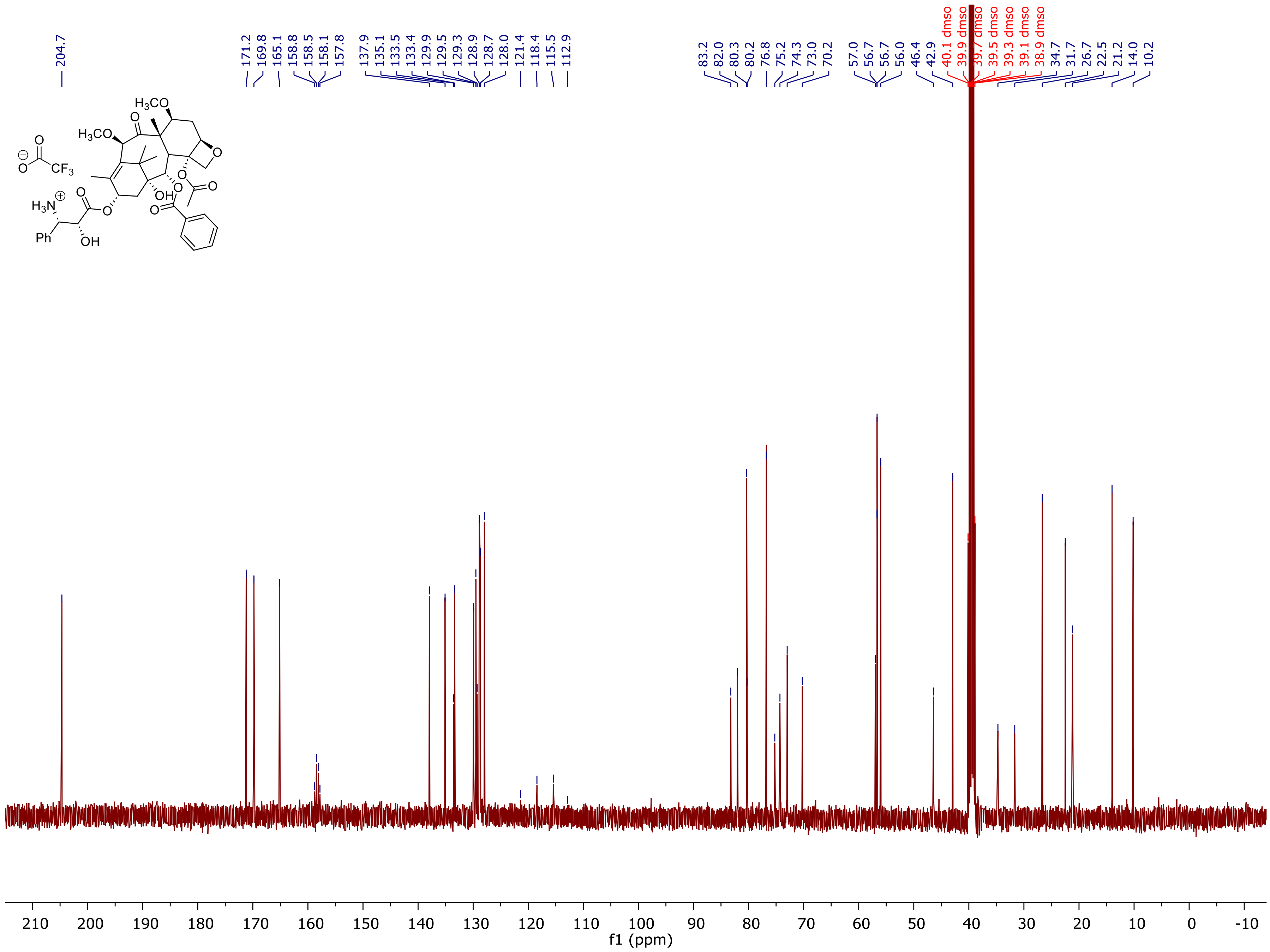

^{1}H NMR of **Probe 1:**

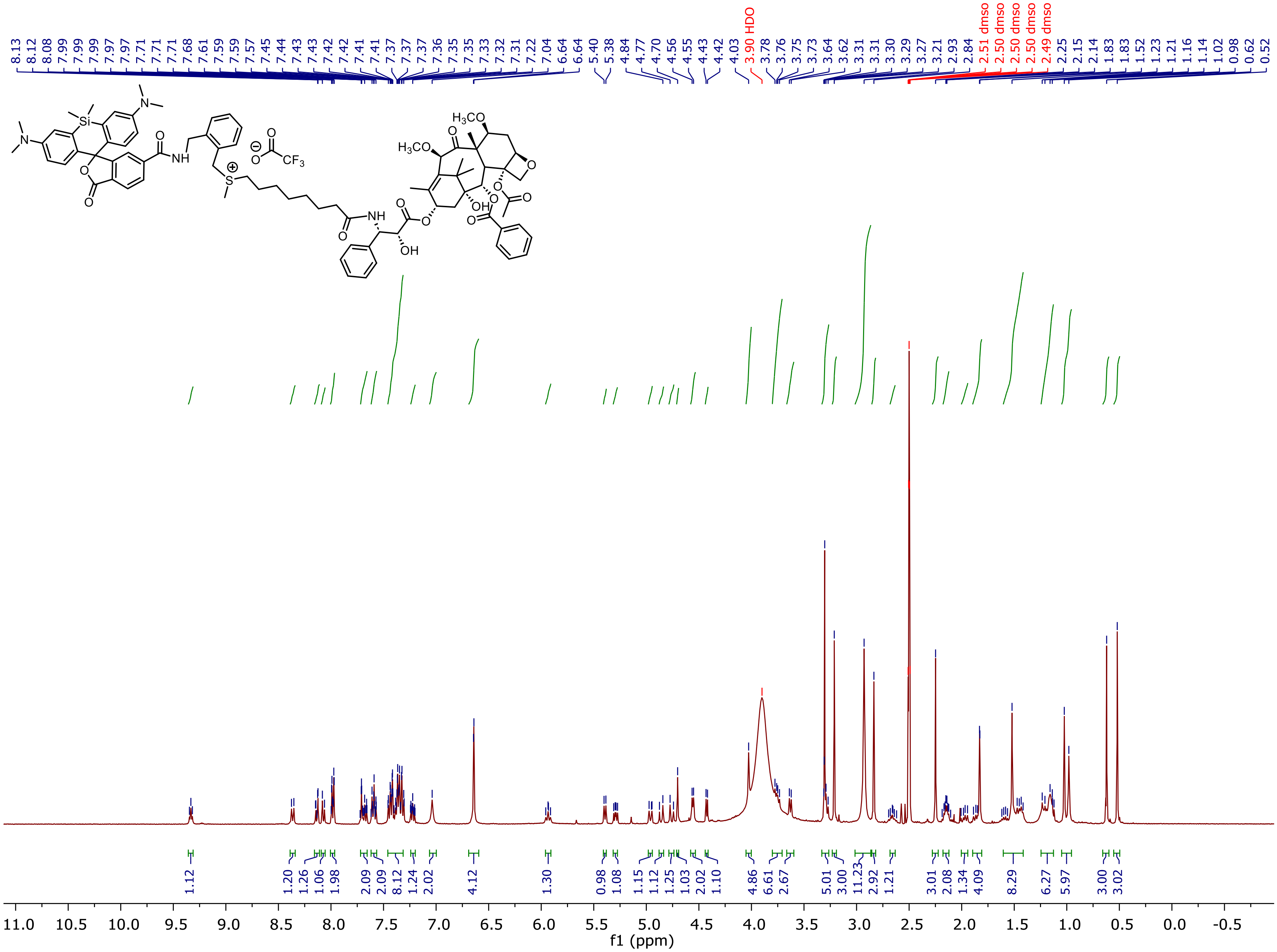

HSQC of **Probe 1:**

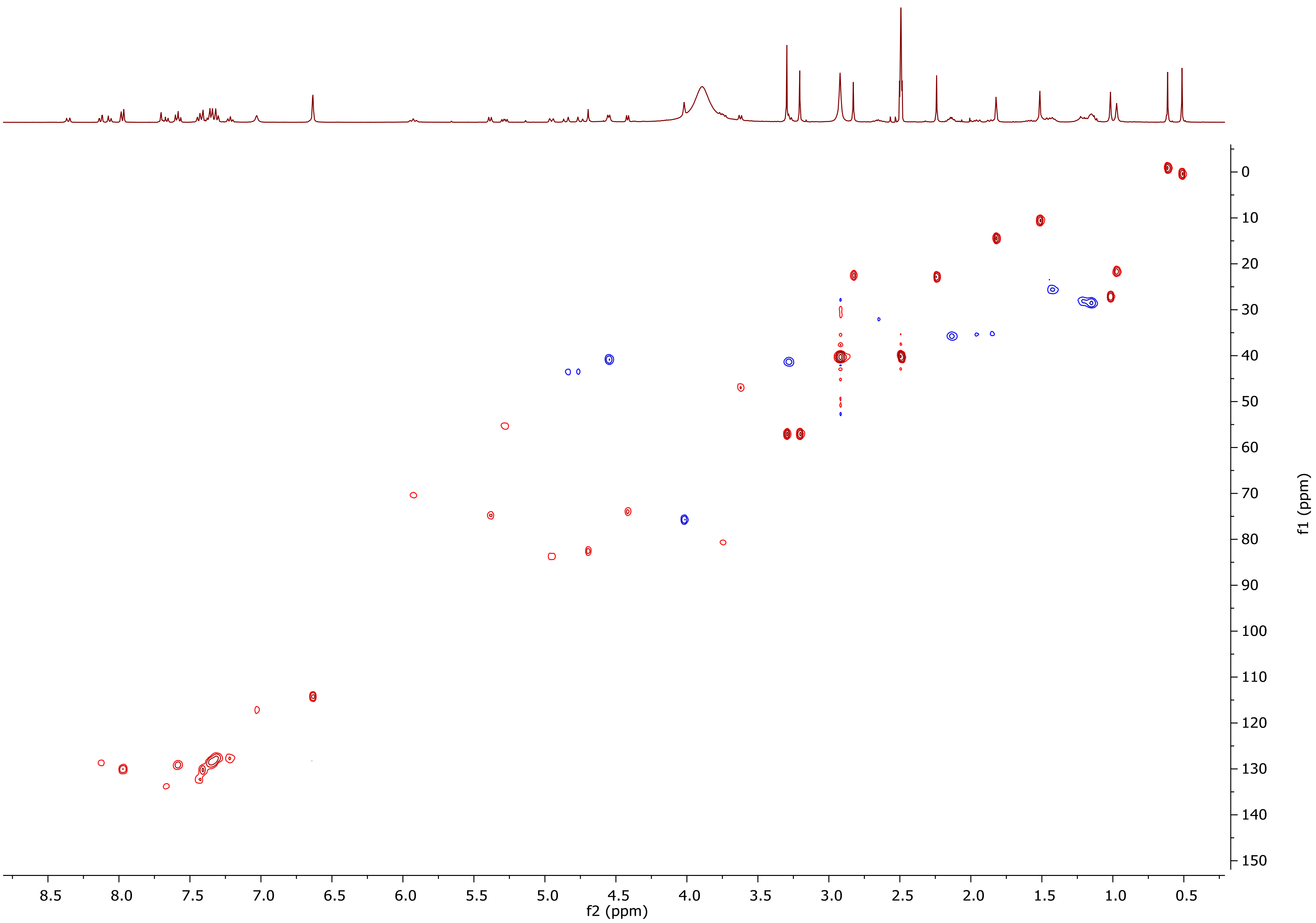

^{1}H NMR of **Probe 2 - 6-SiR-*o*-C$_9$-CTX:**

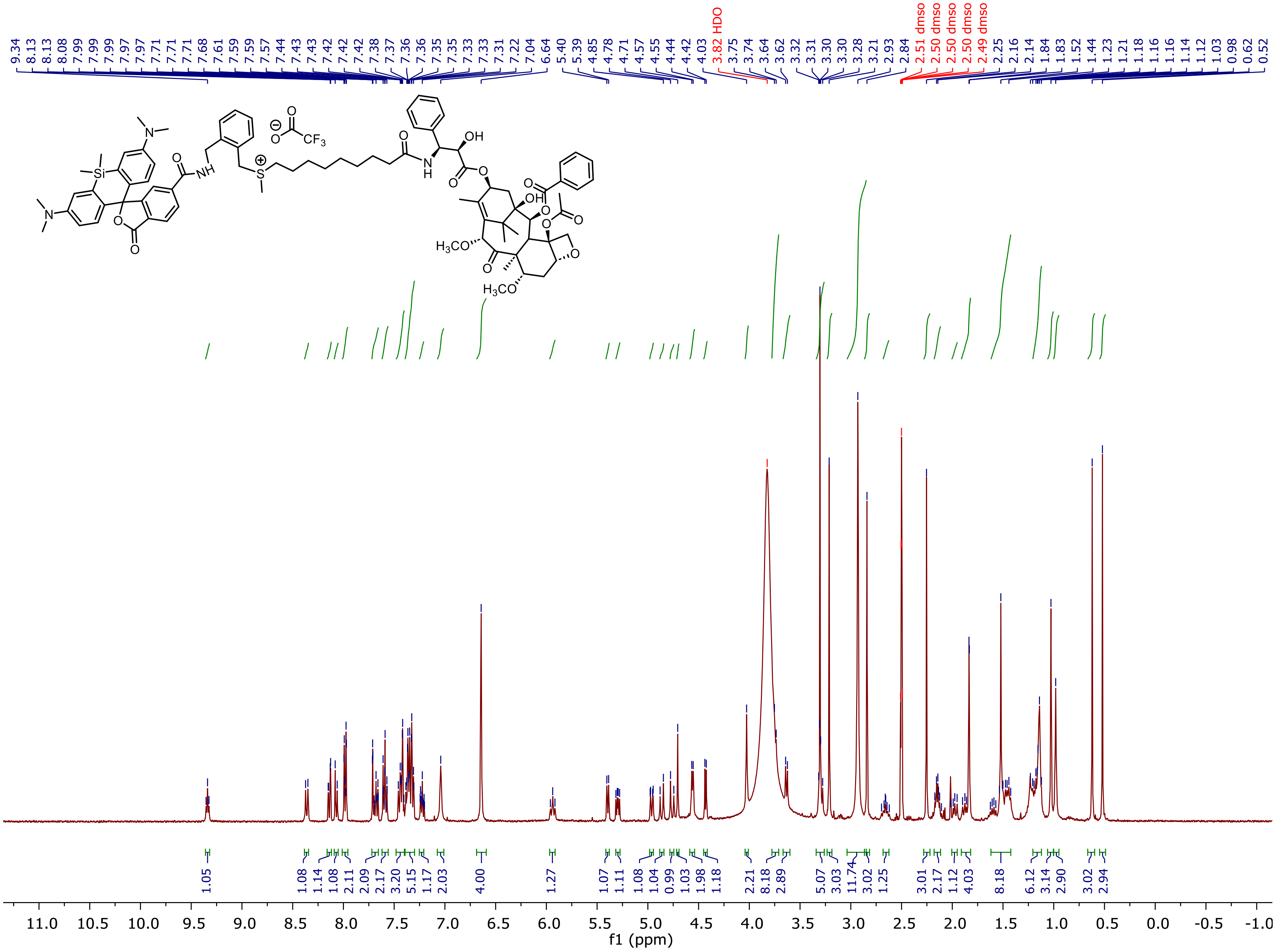

HSQC of **Probe 2 - 6-SiR-*o*-C_9-CTX:**

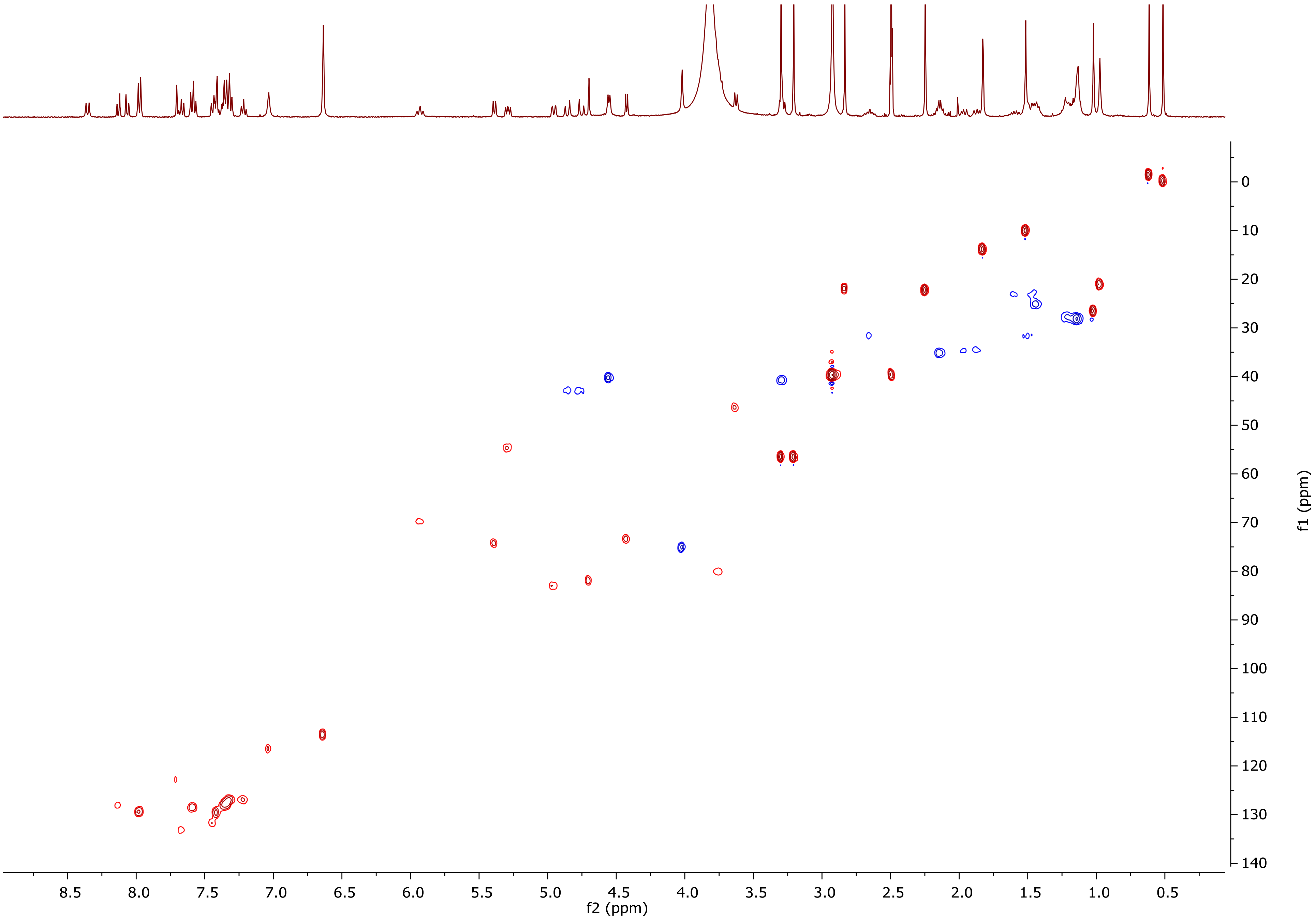

^{1}H NMR of **Probe 3:**

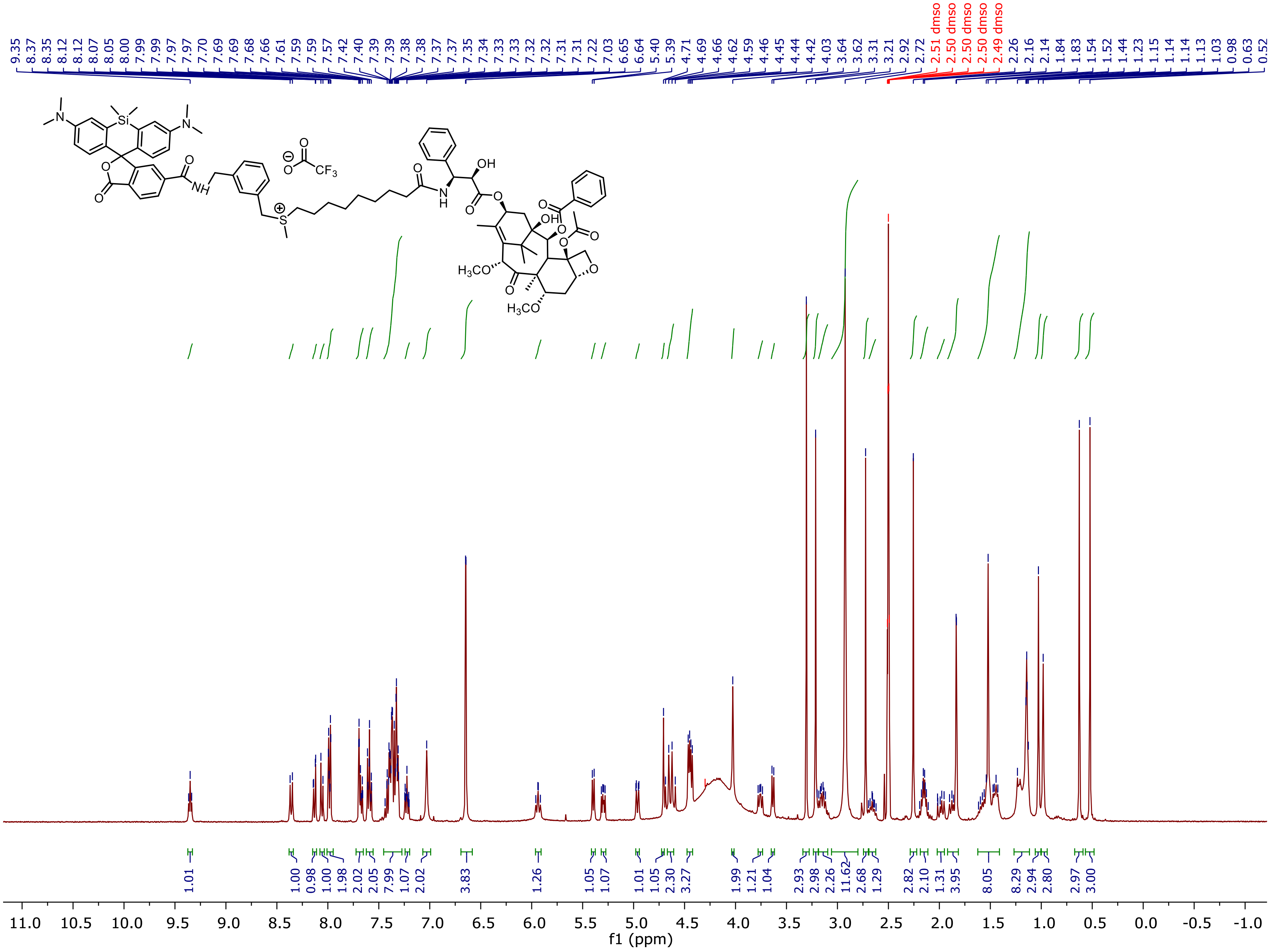

HSQC of **Probe 3:**

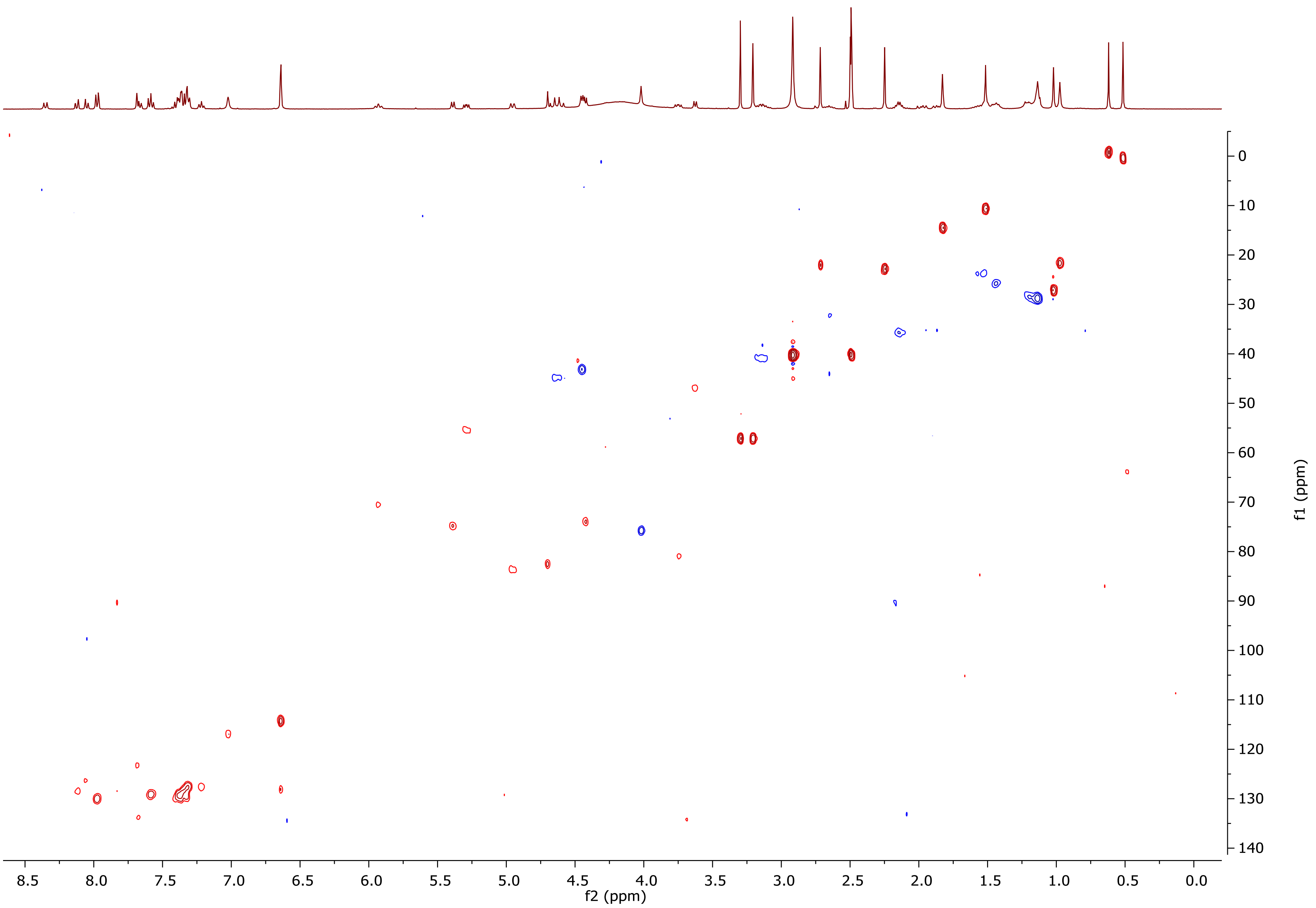

^{1}H NMR of **Probe 4:**

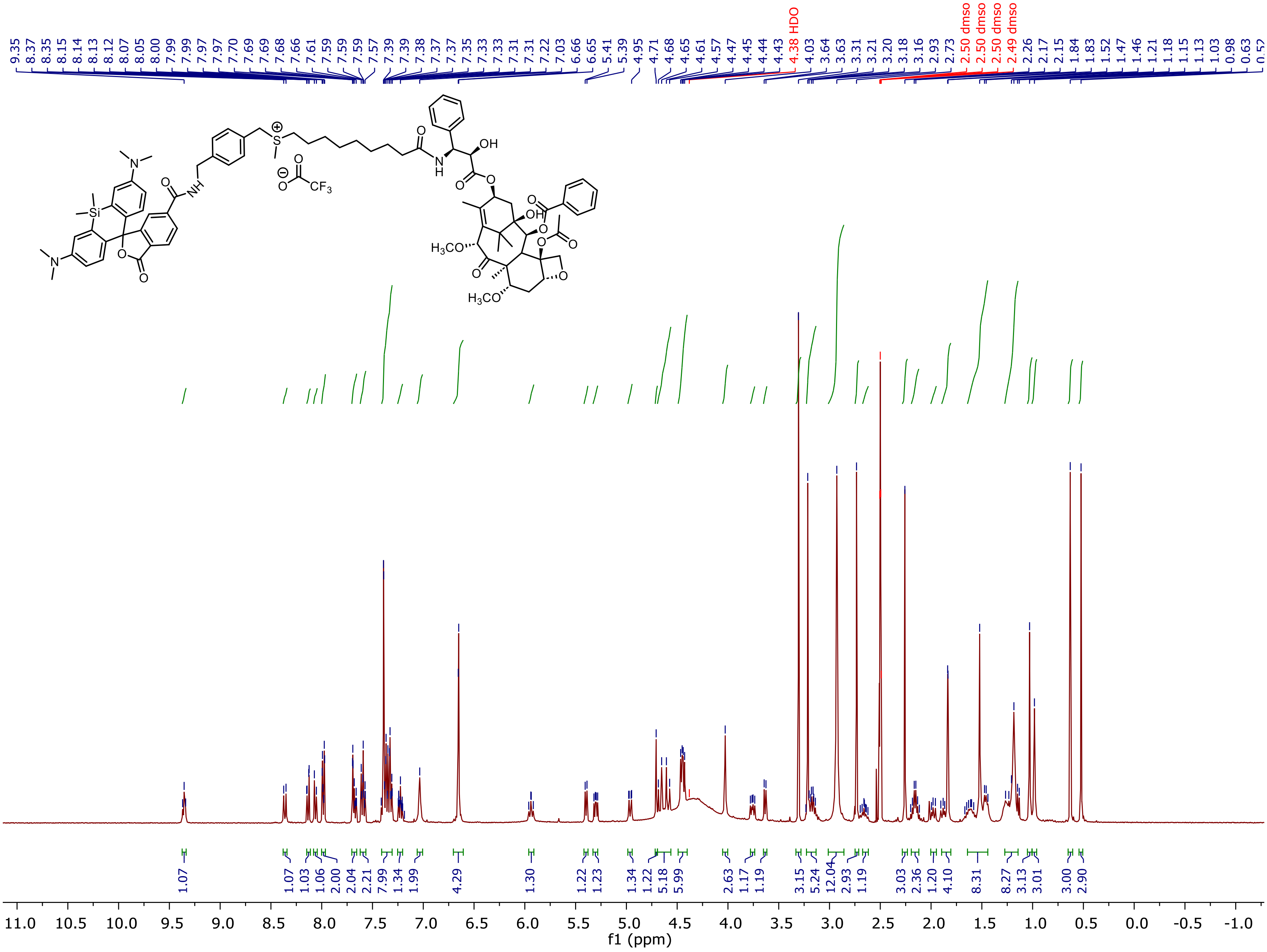

COSY of **Probe 4:**

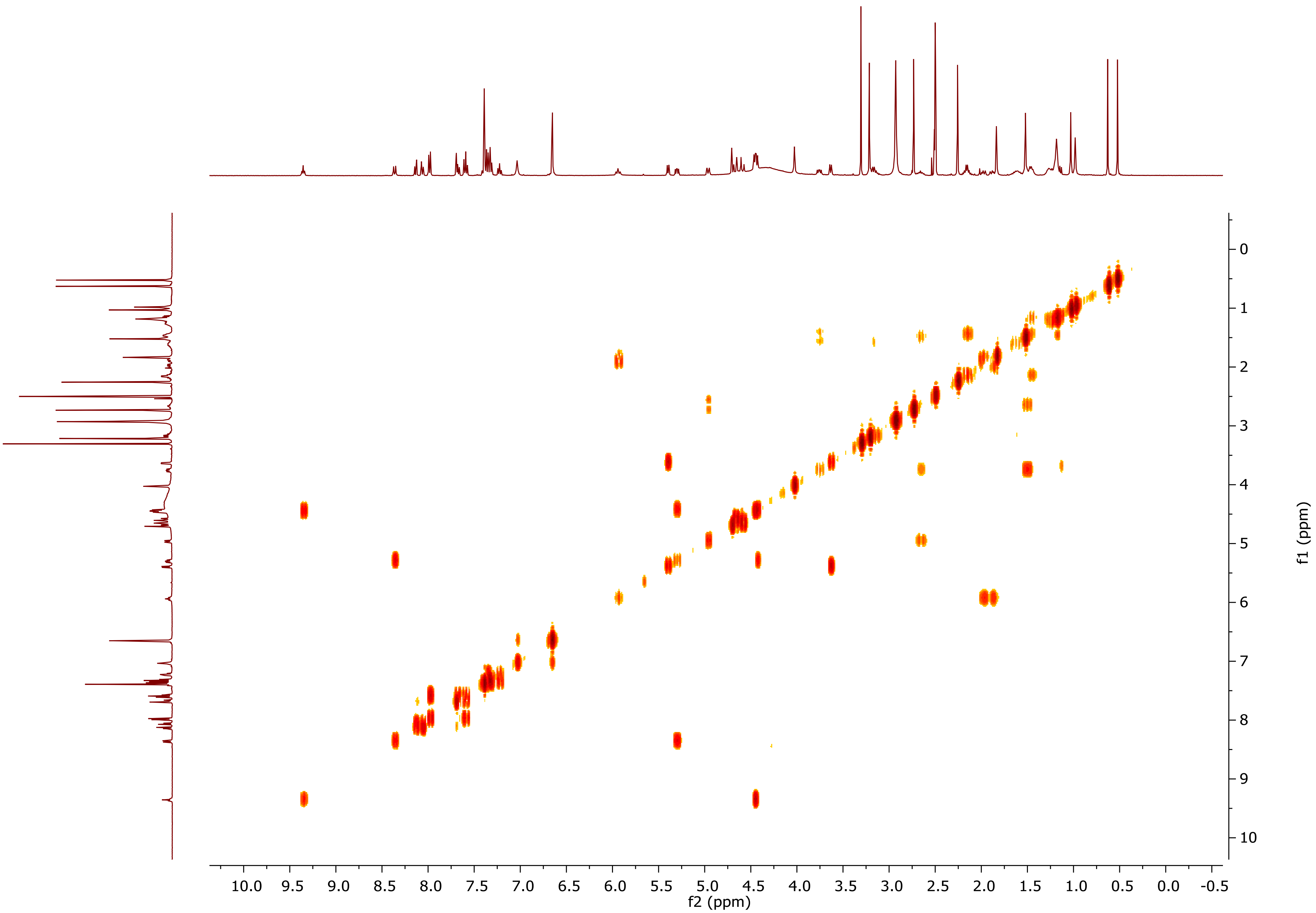

^{1}H NMR of **Probe 5:**

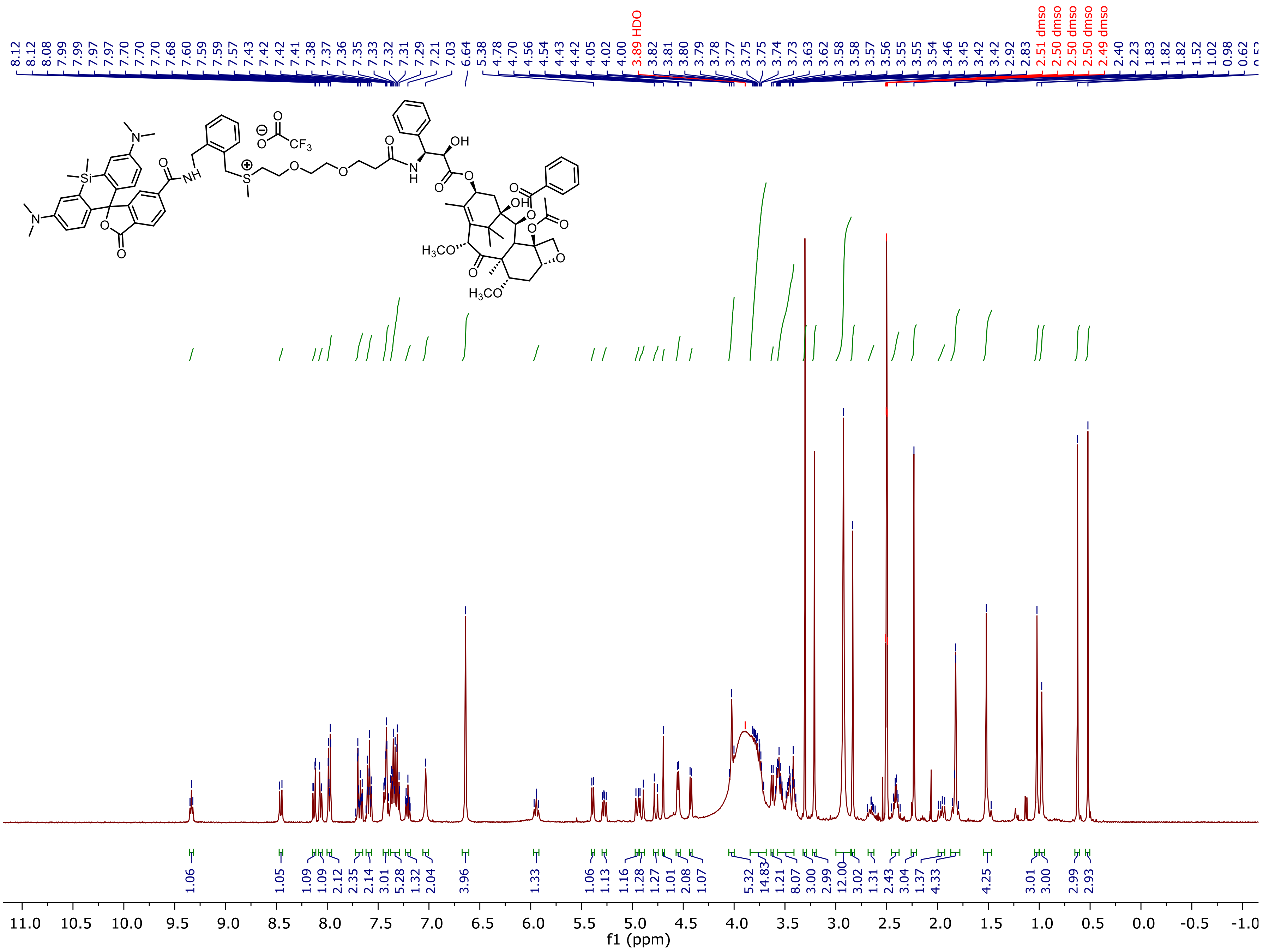

HSQC of **Probe 5:**

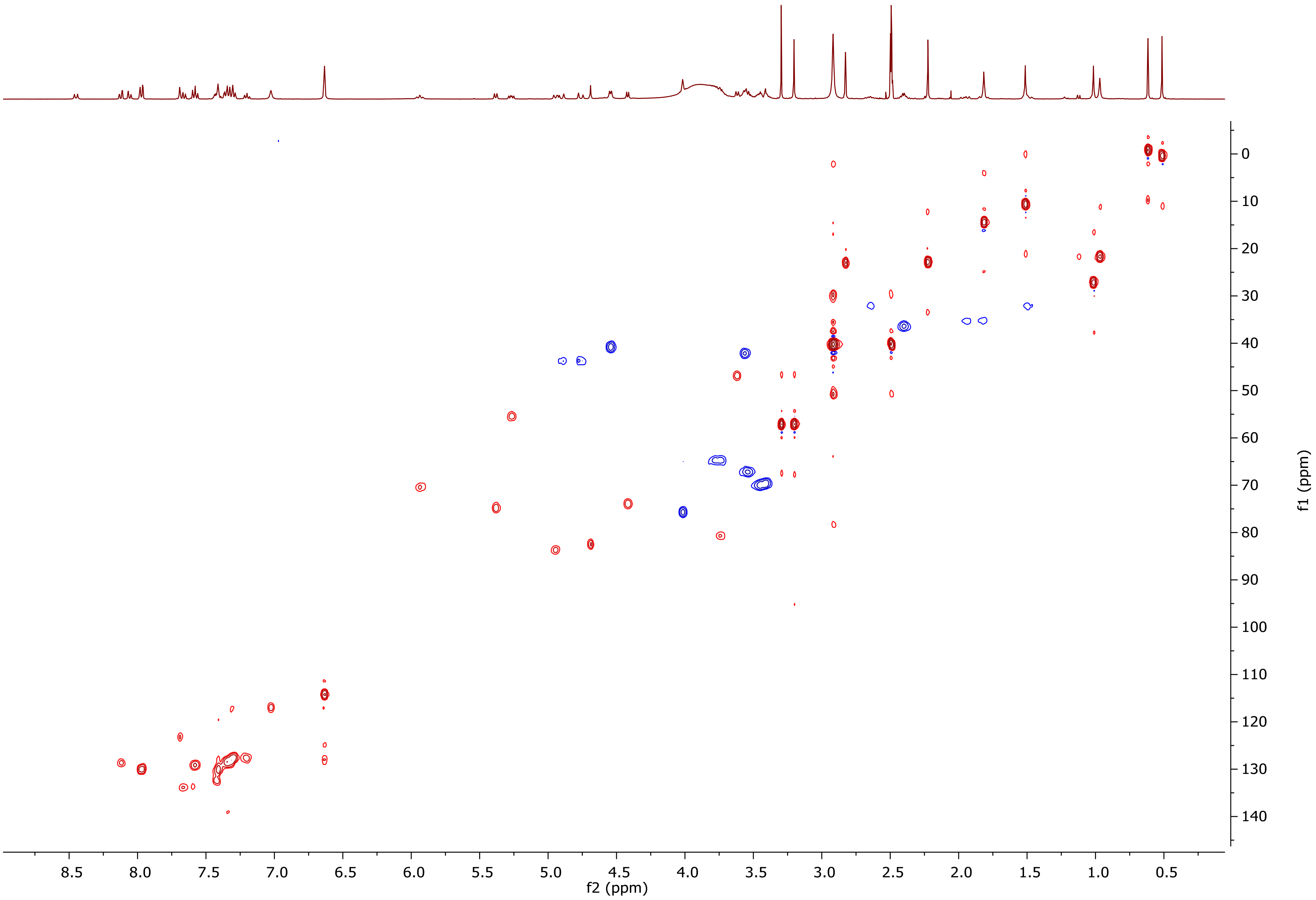

^{1}H NMR of **Probe 6:**

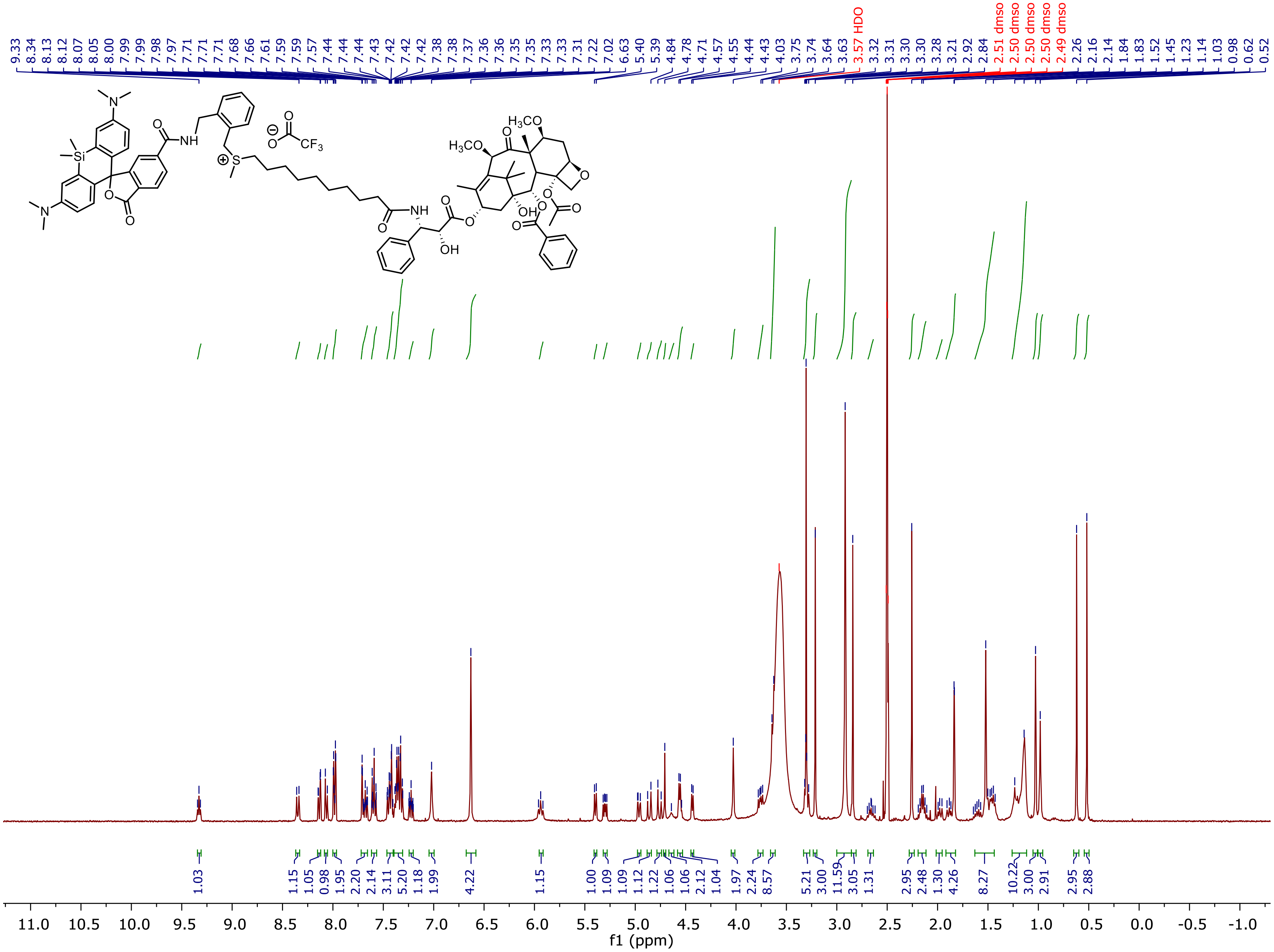

HSQC of **Probe 6:**

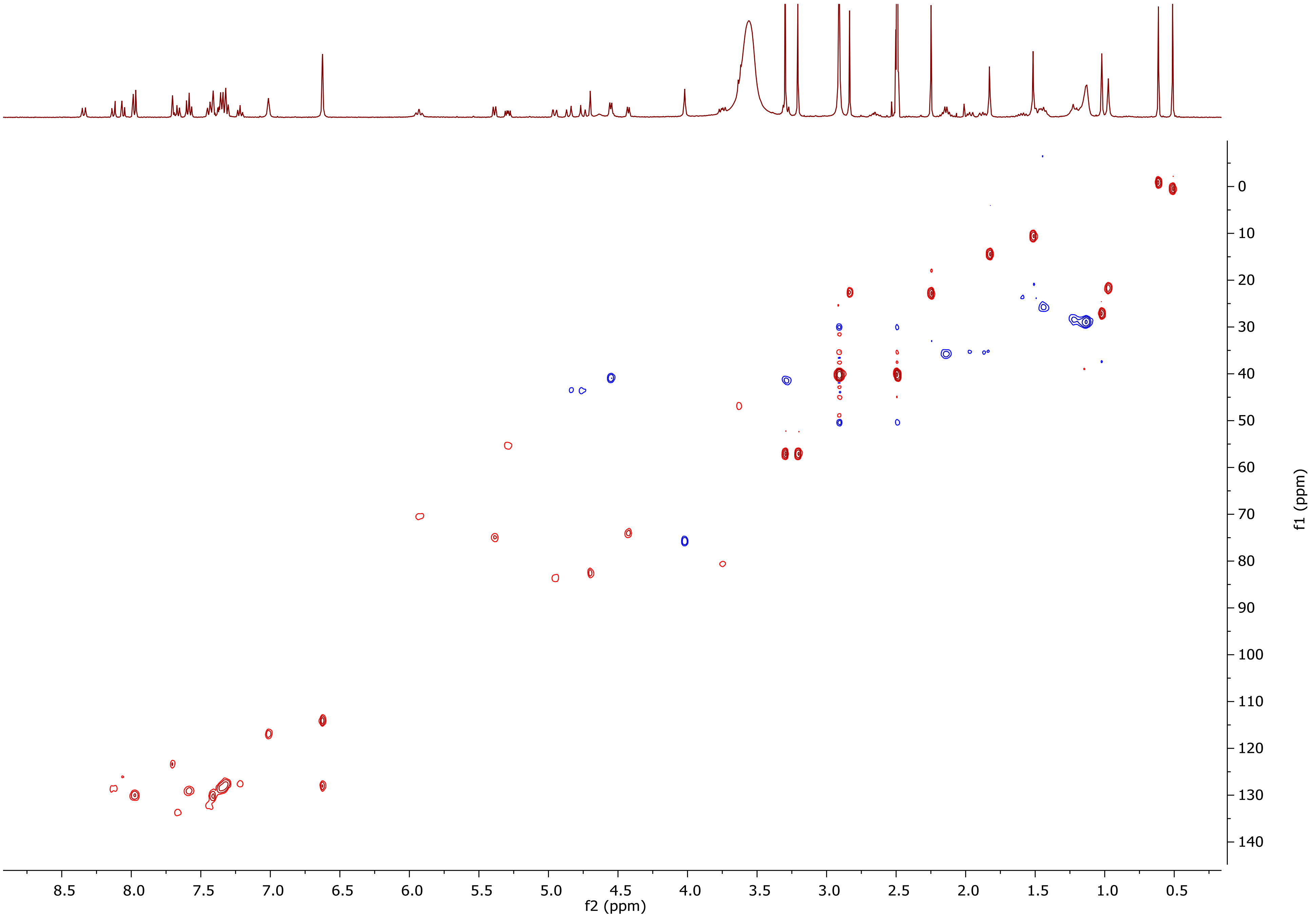

Supplementary references

1 Lukinavičius, G. *et al.* Fluorescent dyes and probes for super-resolution microscopy of microtubules and tracheoles in living cells and tissues. *Chem Sci* **9**, 3324-3334, doi:10.1039/c7sc05334g (2018).

2 Bucevičius, J., Keller-Findeisen, J., Gilat, T., Hell, S. W. & Lukinavičius, G. Rhodamine-Hoechst positional isomers for highly efficient staining of heterochromatin. *Chem Sci* **10**, 1962-1970, doi:10.1039/c8sc05082a (2019).
