# Supplementary material for: Biocompatible sulfonium-based covalent probes for endogenous tubulin fluorescence nanoscopy in live and fixed cells
Source: Nat Commun. 2025 Dec 11;16:11107. doi: 10.1038/s41467-025-67319-x (PMC12701065; doi:10.1038/s41467-025-67319-x)
Supplement: Supplementary file 3 — Description of Additional Supplementary Files [file 41467_2025_67319_MOESM3_ESM.pdf]

Supplementary Data 1.
Mass spectrometric identification and localization of the SiR-modification site. Data shown in the attached Excel file.

Supplementary Movie 1.
Labeling sites on β-tubulin. Structure of β-tubulin (from pig – PDB: 5SYF) with Taxol. The main labeling site is highlighted in blue (Cys 356), and the minor labeling site in red (Cys 12).

Supplementary Movie 2.
STED timelapse of living dermal fibroblasts stained with 6-SiR-o-C9-CTX. Live human dermal fibroblasts incubated with 6-SiR-o-C9-CTX (1 μM in OptiMEM) for 2h, washed three times with HBSS and imaged in DMEM+. A 500 sec timelapse was acquired (one frame per every 25 sec) with Abberior Facility Line. Scale bar = 20 μm
